# The InBIO Barcoding Initiative Database: DNA barcodes of Portuguese moths

**DOI:** 10.3897/BDJ.12.e117169

**Published:** 2024-05-16

**Authors:** Sónia Ferreira, Martin F. V. Corley, João Nunes, Jorge Rosete, Sasha Vasconcelos, Vanessa A. Mata, Joana Veríssimo, Teresa L Silva, Pedro Sousa, Rui Andrade, José Manuel Grosso-Silva, Catarina J. Pinho, Cátia Chaves, Filipa MS Martins, Joana Pinto, Pamela Puppo, Antonio Muñoz-Mérida, John Archer, Joana Pauperio, Pedro Beja

**Affiliations:** 1 CIBIO, Centro de Investigação em Biodiversidade e Recursos Genéticos, InBIO Laboratório Associado, Campus de Vairao, Universidade do Porto, 4485-661 Vairao, Vila do Conde, Portugal CIBIO, Centro de Investigação em Biodiversidade e Recursos Genéticos, InBIO Laboratório Associado, Campus de Vairao, Universidade do Porto, 4485-661 Vairao Vila do Conde Portugal; 2 BIOPOLIS Program in Genomics, Biodiversity and Land Planning, CIBIO, Campus de Vairao, 4485-661 Vairao, Vila do Conde, Portugal BIOPOLIS Program in Genomics, Biodiversity and Land Planning, CIBIO, Campus de Vairao, 4485-661 Vairao Vila do Conde Portugal; 3 EBM, Estação Biológica de Mértola, Praça Luís de Camões, Mértola, Portugal EBM, Estação Biológica de Mértola, Praça Luís de Camões Mértola Portugal; 4 Pucketty Farm Cottage, Faringdon, Oxfordshire SN7 8JP, U.K, Oxfordshire, United Kingdom Pucketty Farm Cottage, Faringdon, Oxfordshire SN7 8JP, U.K Oxfordshire United Kingdom; 5 Rua Eduardo Joaquim Reis Figueira, 1104 RC AR, 4440-647, Valongo, Portugal Rua Eduardo Joaquim Reis Figueira, 1104 RC AR, 4440-647 Valongo Portugal; 6 Urbanização Lourisol, Rua Manuel Cerqueira Nobrega, Lote 16, 2. frente, P-3105-165 Louriçal, Pombal, Portugal Urbanização Lourisol, Rua Manuel Cerqueira Nobrega, Lote 16, 2. frente, P-3105-165 Louriçal Pombal Portugal; 7 CIBIO, Centro de Investigação em Biodiversidade e Recursos Genéticos, InBIO Laboratório Associado, Instituto Superior de Agronomia, Universidade de Lisboa, Lisboa, Portugal CIBIO, Centro de Investigação em Biodiversidade e Recursos Genéticos, InBIO Laboratório Associado, Instituto Superior de Agronomia, Universidade de Lisboa Lisboa Portugal; 8 Department of Ecology, Swedish University of Agricultural Sciences, PO Box 7044, 750 07, Uppsala, Sweden Department of Ecology, Swedish University of Agricultural Sciences, PO Box 7044, 750 07 Uppsala Sweden; 9 Departamento de Biologia, Faculdade de Ciências, Universidade do Porto, 4169-007, Porto, Portugal Departamento de Biologia, Faculdade de Ciências, Universidade do Porto, 4169-007 Porto Portugal; 10 International Union for Conservation of Nature (IUCN), Species Survival Commission (SSC), Spider and Scorpion Specialist Group, Gland, Switzerland International Union for Conservation of Nature (IUCN), Species Survival Commission (SSC), Spider and Scorpion Specialist Group Gland Switzerland; 11 Rua Calouste Gulbenkian 237 4H3, 4050-145, Porto, Portugal Rua Calouste Gulbenkian 237 4H3, 4050-145 Porto Portugal; 12 Museu de História Natural e da Ciência da Universidade do Porto, Porto, Portugal Museu de História Natural e da Ciência da Universidade do Porto Porto Portugal; 13 Marshall University, Department of Biological Sciences, Huntington, West Virginia, United States of America Marshall University, Department of Biological Sciences Huntington, West Virginia United States of America; 14 CIBIO, Centro de Investigação em Biodiversidade e Recursos Genéticos, InBIO Laboratório Associado, Campus de Vairão, Universidade do Porto,, 4485–661 Vairão, Vila do Conde, Portugal CIBIO, Centro de Investigação em Biodiversidade e Recursos Genéticos, InBIO Laboratório Associado, Campus de Vairão, Universidade do Porto, 4485–661 Vairão, Vila do Conde Portugal; 15 European Molecular Biology Laboratory, European Bioinformatics Institute, Hinxton, Cambridge, United Kingdom European Molecular Biology Laboratory, European Bioinformatics Institute Hinxton, Cambridge United Kingdom

**Keywords:** Lepidoptera, occurrence records, species distributions, continental Portugal, DNA barcode, cytochrome c oxidase subunit I (COI)

## Abstract

**Background:**

The InBIO Barcoding Initiative (IBI) Dataset - DS-IBILP08 contains records of 2350 specimens of moths (Lepidoptera species that do not belong to the superfamily Papilionoidea). All specimens have been morphologically identified to species or subspecies level and represent 1158 species in total. The species of this dataset correspond to about 42% of mainland Portuguese Lepidoptera species. All specimens were collected in mainland Portugal between 2001 and 2022. All DNA extracts and over 96% of the specimens are deposited in the IBI collection at CIBIO, Research Center in Biodiversity and Genetic Resources.

**New information:**

The authors enabled "The InBIO Barcoding Initiative Database: DNA barcodes of Portuguese moths" in order to release the majority of data of DNA barcodes of Portuguese moths within the InBIO Barcoding Initiative. This dataset increases the knowledge on the DNA barcodes of 1158 species from Portugal belonging to 51 families. There is an increase in DNA barcodes of 205% in Portuguese specimens publicly available. The dataset includes 61 new Barcode Index Numbers. All specimens have their DNA barcodes publicly accessible through BOLD online database and the distribution data can be accessed through the Global Biodiversity Information Facility (GBIF).

## Introduction

The Portuguese fauna of Lepidoptera is relatively rich with 2775 species recorded so far (Corley et al. 2023). Of these, over 85 species have been described only in this century, with an increasing number of researchers contributing to the knowledge of the country's Lepidopteran fauna. During this period, 17 newly-endemic species have been described (*Phalacropterixfritschi* Hättenschwiler, 2003; *Coleophoralusitanica* Baldizzone & Corley, 2004; *Scrobipalpacorleyi* Huemer & Karsholt, 2010; *Infurcitineacorleyi* Gaedike, 2011; *Dahlicaestrela* (Arnscheid, 2012); *Lobesiaarzilae* Trematerra, 2014; *Willibaldianaculatrae* Trematerra, 2014; *Denisiapiresi* Corley, 2014; *Filatimaalgarbiella* Corley, 2014; *Arogaeatoni* Corley & Goodey, 2014; *Ekboarmiaminiaria* Skou, Stüning & Sihvonen, 2016; *Chrysoclistasoniae* Corley, 2017; *Megacraspedusoccidentellus* Huemer & Karsholt, 2018; *Afriberinasalemae* Skou & Sihvonen, 2019; *Mondeguinaatlanticella* Corley & Rosete, 2020; *Heterogyniscynetis* de Freina, Monasterio, Escobés, Hinojosa & Vila, 2020; *Epermenialusitanica* Gaedike, 2022) (Corley 2015, Skou et al. 2017, Corley 2017, Huemer and Karsholt 2018, Müller et al. 2019, Corley et al. 2020, De Freina et al. 2021, Gaedike 2022) and others are known to exist (Corley, unpublished data). Since 2006, additions have been published on a yearly basis in the series "New and interesting Portuguese Lepidoptera records" (e.g. Corley et al. (2018), Corley et al. (2019b)). In the last two decades, over 500 species have been added and an annotated checklist was published in 2015 (Corley 2015). In 2021, a distribution dataset with over 50,000 records referring to specimens of 68 families, 925 genera and 2311 species/subspecies has been released in GBIF (Corley and Afonso 2021). Additionally, in 2021, a new citizen-science project created the Portuguese Moth Recording Scheme called "Rede de Estações de Borboletas Nocturnas" (REBN). This project promotes public participation in the production of faunistic data. It released a first dataset that consists of a collection of personal observations by the participants (Nunes et al. 2023) and the records produced will continue to be released at regular intervals.

In parallel with the efforts to produce and make available reliable data on the taxonomy and chorology of Portuguese moths, the IBI was created - the InBIO Barcoding Initiative, a DNA barcoding initiative by the Research Network in Biodiversity and Evolutionary Biology - InBIO as a result of the paucity of genetic data on Portuguese biodiversity. The InBIO Barcoding Initiative (IBI) makes use of High-Throughput Sequencing technologies to construct a reference collection of morphologically identified Portuguese specimens and respective DNA barcodes (e.g. Rebelo et al. 2020, Ferreira et al. 2021, Oliveira et al. 2021, Pauperio et al. 2023, Rosa et al. 2023). DNA barcoding offers a rapid, cost-effective alternative tool for both the identification of described species and the discovery of new ones (Hebert et al. 2003, Savolainen et al. 2005, Mitchell 2008, our papers). In fact, a few new species have seen their distinctness confirmed by DNA barcodes leading to their description including *Ypsolopharhinolophi* Corley, 2019 that was found to be part of the diet of horseshoe bats in a study using DNA metabarcoding (e.g. Martin and Ferreira (2019), Corley et al. (2019a), Corley et al. (2020)). DNA barcoding provides a powerful tool by using a short fragment of DNA to assign any organism to a species in a rapid and automated way (Hebert, Cywinska, Ball and DeWaard 2003). The number of DNA barcode reference sequences available in public databases has increased remarkably and DNA barcoding has been broadly adopted (e.g. Hebert et al. 2003,Mortágua et al. 2019, Azevedo et al. 2020, da Silva et al. 2020, Fais et al. 2020, Mata et al. 2020, Velarde‐Garcéz et al. 2023). However, for many groups and geographical regions, these databases are still very incomplete, which limits the general application of DNA barcoding in biodiversity research (Elbrecht et al. 2017). DNA barcodes of Lepidoptera of Iberian Peninsula are being progressivelly documented in recent years (e.g. Ortiz et al. 2017, Ortiz et al. 2023) and the present work represents the first project to generate DNA barcodes for Portuguese moths at a faunal level and, thus, represents a major step in documenting the genetic diversity of the Portuguese moth fauna.

## General description

### Purpose

This dataset aims to provide a contribution to the knowledge on DNA barcodes of Portuguese moths. Such a library should facilitate DNA-based identification of species for both traditional molecular studies and DNA metabarcoding studies and constitute a valuable resource for taxonomic and ecological research on Lepidoptera, with the focus on the Portuguese fauna.

### Additional information

**Figure 3.**
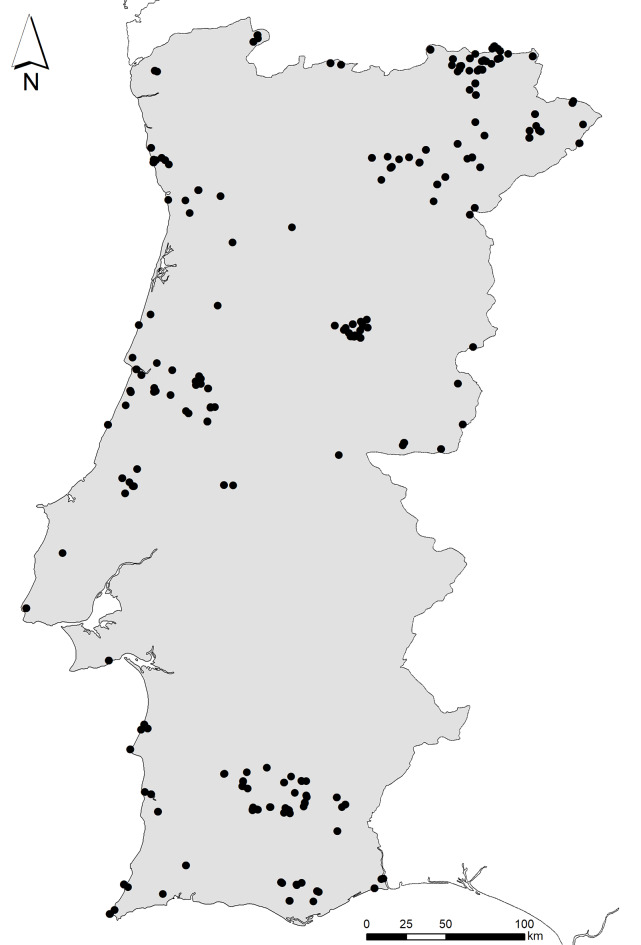
Map of the localities where DNA barcoded Lepidoptera samples were collected in continental Portugal.

Figs 1, 2 illustrate examples of the diversity of species that are part of the dataset of distribution data and DNA barcodes of Portuguese moths. All sequences of cytochrome c oxidase I (COI) DNA barcodes are 658 base pairs (bp) long, except for one with 422 bp (Fig. 3, Suppl. material 1). Sequences are distributed in 1199 Barcode Index Numbers (BINs), 63 being unique to this dataset. Average nucleotide composition of the sequences is 39.5% thymine (T), 15.5% cytosine (C), 30.5% adenine (A) and 14.6% guanine (G), for a total GC content of 30.1% for the COI barcode fragment analysed. Genetic p-distances ranged from 0.00% between the pair *Pleurotahonorella* and *Pleurotaplanella* as previously found with other specimens in BIN BOLD:AEC9855; and 18.5% between *Tineatrinotella* and *Tineamurariella*. Intraspecific genetic p distances ranged from 0.00% to 11.1% in *Pleurotabicostella* group. BOLD Systems retrieved BINs to all specimens in the dataset (Ratnasingham and Hebert 2013). Only 4% of the species with more than one specimen (n = 670) had sequences assigned to two BINs (n = 31). Two species in our dataset present up to three BINs, namely *Ephestiawelseriella* (BOLD:ABW1548, BOLD:AEC9178 and BOLD:AEC9179) and *Cydiafagiglandana* (BOLD:AAC5023, BOLD:ACS3074 and BOLD:AEB8145). Multiple BINs are relatively frequent in Lepidoptera species with higher percentages found in studies with higher number of specimens per species and with larger geographic scope. For example, Huemer et al. (2020) and Dincă et al. (2021) found that 20% of species of European Gelechiidae and 12.2% species of European butterflies exhibit deep splits, while studies of more limited geographical scope found 6% of species in Bavarian Geometrids (Hausmann et al. 2011) or even no deep splits in the case of Maltese islands Vella et al. (2022). The dataset includes 10 shared BINs between taxa possibly in need of taxonomical revision:

BOLD:AAA7740 (*Yponomeutacagnagella* and *Yponomeutaevonymella*); BOLD:AAA9515 (*Chloroclystamiata* and *Chloroclystasiterata*); BOLD:AAD0839 (*Macrothylaciadigramma* and *Macrothylaciarubi*); BOLD:AAB4833 (*Oligiastrigilis* and *Oligiaversicolor*); BOLD:AAD6780 (*Cryphiaalgae* and *Cryphiapallida*); BOLD:AAF0005 (*Lozotaeniodescupressana* and *Lozotaeniodesformosana*); BOLD:ABV4113 (*Cleonymiadiffluens* and *Cleonymiayvanii*); BOLD:ACE8354 (*Euxoaoranaria* and *Euxoatritici*); BOLD:ACY5987 (*Pleurotaandalusica* and *Pleurotaericella*); BOLD:AEC9855 (*Pleurotahonorella* and *Pleurotaplanella*).

From the 2775 species belonging to 77 families recorded from continental Portugal (Corley et al. 2023), 235 species belonging to 40 families remain without DNA barcodes and 26 families have not a single DNA barcoded species (Suppl. material 6). Of these, 103 species have already specimens registered in BOLD SYSTEMS lacking DNA barcodes, while specimens of the remaining 132 species are still needed.

## Project description

### Title

The name "The InBIO Barcoding Initiative Database: DNA barcodes of Portuguese moths" refers to the first data release of DNA barcodes and distribution data of Portuguese moths within the InBIO Barcoding Initiative.

### Personnel

Pedro Beja (project coordinator), Sónia Ferreira (taxonomist and IBI manager), Martin F.V. Corley (taxonomist, lepidopterist), Cátia Chaves (project technician), Filipa M.S. Martins (molecular biologist), Vanessa A. Mata (molecular biologist), Antonio Muñoz-Mérida (bioinformatician), John Archer (bioinformatician), Joana Paupério (molecular biologist), Catarina J. Pinho (project technician), Joana C. Pinto (project technician), Pamela Puppo (molecular biologist), Teresa L Silva (molecular biologist), Pedro Sousa (project technician), Sasha Vasconcelos (contributor), Joana Veríssimo (molecular biologist), all affiliated to CIBIO-InBIO, University of Porto, José Manuel Grosso-Silva (entomologist), affiliated to the MHNC-UP, University of Porto and Rui Andrade (entomologist), João Nunes (lepidopterologist), Jorge Rosete (lepidopterist), independent researchers.

### Study area description

Continental Portugal (Fig. 3).

### Design description

Lepidoptera specimens were collected in the field, morphologically identified and DNA barcoded.

### Funding

The present work was funded by National Funds through FCT-Fundação para a Ciência e a Tecnologia in the scope of the project LA/P/0048/2020. InBIO Barcoding Initiative was funded by the European Union’s Horizon 2020 Research and Innovation Programme under grant agreement No 668981 and by the project PORBIOTA – Portuguese E-Infrastructure for Information and Research on Biodiversity (POCI-01-0145- FEDER-022127), supported by Operational Thematic Program for Competitiveness and Internationalization (POCI), under the PORTUGAL 2020 Partnership Agreement, through the European Regional Development Fund (FEDER). The work was partially Funded by Horizon Europe under the Biodiversity, Circular Economy and Environment call (REA.B.3); co-funded by the Swiss State Secretariat for Education, Research and Innovation (SERI) under contract number 22.00173; and by the UK Research and Innovation under the Department for Business, Energy and Industrial Strategy’s Horizon Europe Guarantee Scheme. The fieldwork benefitted from EDP Biodiversity Chair, the project “Promoção dos serviços deecossistemas no Parque Natural Regional do Vale do Tua: Controlo de Pragas Agrícolas eFlorestais por Morcegos” funded by the Agência de Desenvolvimento Regional do Vale doTua and includes research conducted at the Long Term Research Site of Baixo Sabor (LTER_EU_PT_002). SF and VM were funded by the FCT through the programme ‘Stimulus of Scientific Employment, Individual Support—3rd Edition’ (https://doi.org/10.54499/2020.03526.CEECIND/CP1601/CT0010; https://doi.org/10.54499/2020.02547.CEECIND/CP1601/CT0006). CJP, JV and FMSM by PhD grants (SFRH/BD/145851/2019; SFRH/BD/133159/2017; SFRH/BD/104703/2014) funded by FCT.

## Sampling methods

### Study extent

Continental Portugal (Fig. 3).

### Sampling description

Specimens were collected during field expeditions throughout continental Portugal, from 2001 to 2020. They were captured at night using light traps, the latter with UV LEDs, mixed light or mercury vapour lamps or during the day by direct search. All specimens were observed in the field and, in most cases, they could be readily identified to species level by an experienced taxonomist (Martin Corley). Such specimens were preserved in 96% ethanol and stored at the InBIO Barcoding Initiative reference collection (Vairão, Portugal), where they can be re-examined and genitalia dissected, if needed. Specimens that could not be identified in the field (n = 84) were pinned and dried for subsequent examination in the laboratory. They were then stored at the Research Collection of Martin Corley or the Private Collections of Jorge Rosete or J.M. Grosso-Silva.

DNA extraction was performed using either the 96-Well Plate Animal Genomic DNA Mini-Preps Kit (Bio Basic, Ontario, Canada) or the QIAamp DNA Micro Kit (Qiagen, Germany) which is designed to extract higher concentrations of genetic material from samples with small amounts of DNA. Amplification was performed using two different primer pairs that amplify partially overlapping fragments (LC + BH) of the 658 bp barcoding region of the COI mitochondrial gene. We used the primers FwhF1 (Vamos et al. 2017) + C_R (Shokralla et al. 2015) for LC and BF3 (Elbrecht et al. 2019) + BR2 (Elbrecht and Leese 2017) for BH amplification, all modified with 5’ adaptors sequences to be compatible with a two-step protocol. PCRs were performed in 10 μl reactions, containing 5 μl of Multiplex PCR Master Mix (Qiagen, Germany), 0.3 μl of each 10 mM primer and 1-2 μl of DNA, with the remaining volume in water. PCR cycling conditions consisted of an initial denaturation at 95ºC for 15 min, followed by 45 cycles of denaturation at 95ºC for 30 sec, annealing at 45ºC and 50ºC, respectively, for 45 sec and extension at 72ºC for 45 sec and a final elongation step at 60ºC for 10 min.

Successful amplification was validated through 2% agarose gel electrophoresis stained with GelRed (Biotium, USA) and samples selected for sequencing proceeded for a second-round PCR where Illumina P5 and P7 adapters with custom 7 bp long barcodes were attached to each first PCR product. The second PCR was performed in a volume of 10 μl, including 5 μl of KAPA HiFi PCR Kit (KAPA Biosystems, Cape Town, South Africa), 0.5 μl of each 10 mM indexing primer and 2 μl of diluted first PCR product (usually 1:4). PCR cycling conditions were as follows: initial denaturation at 95ºC for 3 min, with 8-10 cycles (adjusted to sample quality) of denaturation at 95ºC for 30 sec, annealing at 50ºC for 60 sec and extension at 72ºC for 45 sec and a final elongation step at 60ºC for 10 min. The amplicons were purified using AMPure XP beads (Beckman Coulter, U.S.A.) and quantified using NanoDrop 1000 (Thermo Scientific, U.S.A.). Clean PCR products were then pooled equimolarly per fragment. Each pool was quantified with KAPA Library Quantification Kit Illumina Platforms (KAPA Biosystems, Cape Town, South Africa) and the 2200 Tapestation System (Agilent Technologies, California, USA) was used for fragment length analysis prior to sequencing (Paupério et al. 2018). DNA sequencing was done at CIBIO facilities on an Illumina MiSeq benchtop system, using V2 MiSeq sequencing kits (2 x 250 bp) (Illumina, California, U.S.A.).

Illumina sequencing reads were processed using OBITools (Boyer et al. 2015) and VSEARCH (Rognes et al. 2016). Briefly, paired-end reads were aligned, collapsed into exact sequence variants, filtered by length, denoised and checked for chimeras. The resulting sequences from both LC and BH fragments of each sample were further assembled using CAP3 (Huang and Madan 1999) to produce a single 658 bp contig per sample.

### Quality control

All DNA barcodes sequences were compared against the BOLD database and the 99 top hits were inspected in order to detect possible issues due to contaminations or misidentifications.

### Step description

1. Specimens were collected in 234 different localities. Fieldwork was carried out between 2001 and 2022.

2. Selected specimens were pinned and dried and are preserved in three private collections. Otherwise, specimens collected as tissue samples were stored in 96% ethanol in the IBI collection at CIBIO, Research Center in Biodiversity and Genetic Resources (Vairão, Portugal).

3. All specimens were morphologically identified and DNA barcoded. To sequence the 658 bp COI DNA barcode fragment, one leg was removed from each individual, DNA was extracted and then amplified. All DNA extracts were deposited in the IBI collection.

4. All sequences in the dataset were submitted to BOLD and GenBank databases and, to each sequenced specimen, the morphological identification was contrasted with the results of the BLAST of the newly-generated DNA barcodes in the BOLD Identification Engine.

## Geographic coverage

### Description

Continental Portugal

### Coordinates

36.960 and 42.124 Latitude; -9.467 and -6.229 Longitude.

## Taxonomic coverage

### Description

**Figure 4.**
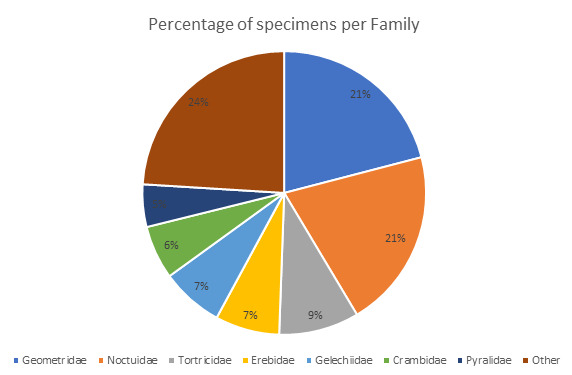
Distribution of specimens, in percentage, per moth family present in the dataset. Families representing less than 3% of the total specimens are represented together in the respective graph.

**Figure 5.**
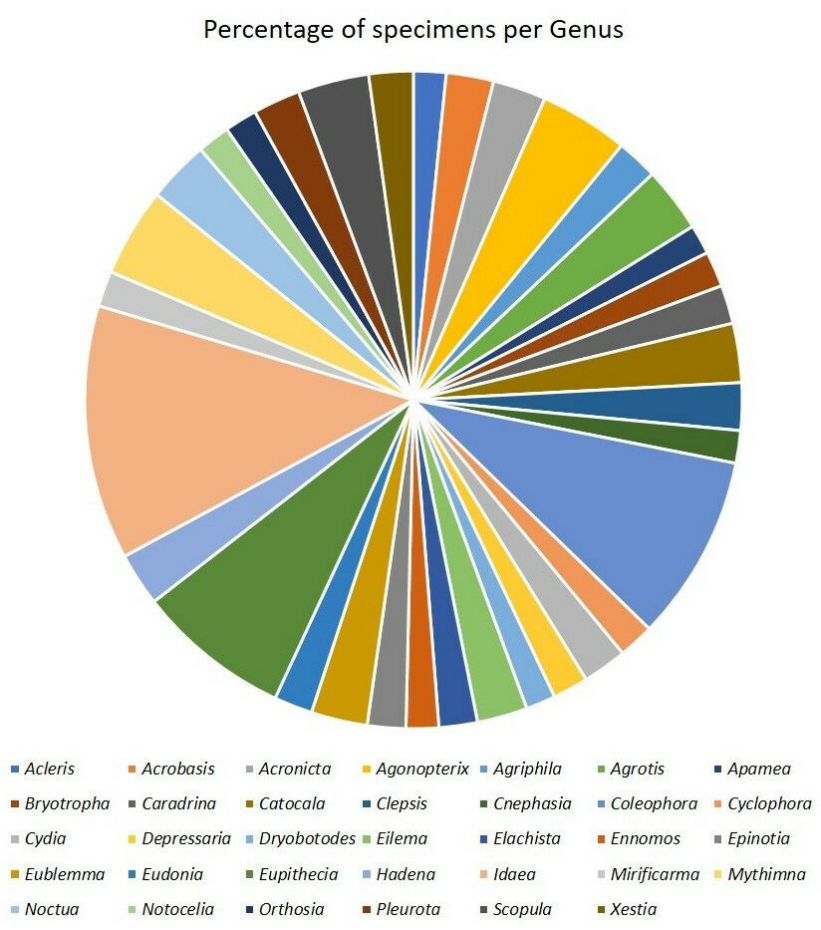
Distribution of specimens, in percentage, per moth genus present in the dataset. Genera represented by less than 0.4% (of total specimens) are not represented in the graph and correspond to 71% of genera in the dataset.

This dataset is composed of data relating to 2364 Lepidoptera specimens. All specimens were determined to species level. Overall, 1170 species are represented in the dataset. These species belong to 51 families and 598 genera (Suppl. material 1). The dataset is represented mostly by seven families that include more than 75% of the specimens, of which Noctuidae, Geometridae and Tortricidae account for more than 20% of each of them (Fig. 4). Twenty-six families known to be present in Portugal are not represented in the dataset. The *Idaea* genus accounts for 4% of the total collected specimens and five other genera accounts for 1 - 3% (Fig. 5).

## Temporal coverage

### Notes

The sampled material was collected in the period from 2001 to 2020.

## Collection data

### Collection name

InBIO Barcoding Initiative

### Collection identifier

4ec2b246-f5fa-4b90-9a8d-ddafc2a3f970

### Specimen preservation method

"Dry", "Alcohol”

### Curatorial unit

Dry voucher - 1- 84, Voucher tube - 1 to 2286, DNA extractions - 1 to 2364

## Usage licence

### Usage licence

Creative Commons Public Domain Waiver (CC-Zero)

## Data resources

### Data package title

The InBIO Barcoding Initiative Database: contribution to the knowledge on DNA barcodes of moths (Lepidoptera)

### Resource link

http://dx.doi.org/10.5883/DS-IBILP08

### Number of data sets

1

### Data set 1.

#### Data set name

IBI-Lepidoptera 08

#### Data format

dwc, xml, tsv, fasta

#### Description

The InBIO Barcoding Initiative Database: contribution to the knowledge on DNA barcodes of Portuguese moths Lepidoptera dataset can be downloaded from the Public Data Portal of BOLD (http://www.boldsystems.org/index.php/Public_SearchTerms?query=DS-IBILP08) in different formats (data as dwc, xml or tsv and sequences as fasta files). Alternatively, BOLD users can log-in and access the dataset via the Workbench platform of BOLD. All records are also searchable within BOLD, using the search function of the database.

The version of the dataset, at the time of writing the manuscript, is included as Suppl. materials 2, 3, 4, 5 in the form of two text files with specimen data information and one fasta file containing all sequences as downloaded from BOLD.

**Data set 1.** 

Column label	Column description
processid	Unique identifier for the sample.
sampleid	Identifier for the sample being sequenced, i.e. IBI catalogue number at Cibio-InBIO,Porto University. Often identical to the "Field ID" or "Museum.
recordID	Identifier for specimen assigned in the field.
catalognum	Catalogue number.
fieldnum	Field number.
institution_storing	The full name of the institution that has physical possession of the voucher specimen.
bin_uri	Barcode Index Number system identifier.
phylum_taxID	Phylum taxonomic numeric code.
phylum_name	Phylum name.
class_taxID	Class taxonomic numeric code.
class_name	Class name.
order_taxID	Order taxonomic numeric code.
order_name	Order name.
family_taxID	Family taxonomic numeric code.
family_name	Family name.
subfamily_taxID	Subfamily taxonomic numeric code.
subfamily_name	Subfamily name.
genus_taxID	Genus taxonomic numeric code.
genus_name	Genus name.
species_taxID	Species taxonomic numeric code.
species_name	Species name.
identification_provided_by	Full name of primary individual who assigned the specimen to a taxonomic group.
identification_method	The method used to identify the specimen.
voucher_status	Status of the specimen in an accessioning process (BOLD controlled vocabulary).
tissue_type	A brief description of the type of tissue or material analysed.
collectors	The full or abbreviated names of the individuals or team responsible for collecting the sample in the field.
lifestage	The age class or life stage of the specimen at the time of sampling.
sex	The sex of the specimen.
lat	The geographical latitude (in decimal degrees) of the geographic centre of a location.
lon	The geographical longitude (in decimal degrees) of the geographic centre of a location.
elev	Elevation of sampling site (in metres above sea level).
country	The full, unabbreviated name of the country where the organism was collected.
province_state	The full, unabbreviated name of the province where the organism was collected.
region	The full, unabbreviated name of the municipality where the organism was collected.
exactsite	Additional name/text description regarding the exact location of the collection site relative to a geographic relevant landmark.

## Supplementary Material

A31654C5-80A8-5A2D-8B57-AB27850CFDCE 10.3897/BDJ.12.e117169.suppl1 Supplementary material 1 List of species that were collected and DNA barcoded within The InBIO Barcoding Initiative Database: DNA barcodes of Portuguese moths Data type occurrences Brief description List of species that were collected and DNA barcoded within this project including the sample code (IBI code), the Process ID (BOLDcode), the BIN URI (BOLD BIN) and GenBank acession number (GenBank). * Indicate species with new BINs. File: oo_960971.txt https://binary.pensoft.net/file/960971 Sónia Ferreira, Martin F. V. Corley, João Nunes, Jorge Rosete, Sasha Vasconcelos, Vanessa A. Mata, Teresa L Silva, Pedro Sousa, Rui Andrade, José Manuel Grosso Silva, Catarina J. Pinho, Cátia Chaves, Filipa MS Martins, Joana Pinto, Pamela Puppo, Antonio Muñoz-Mérida, John Archer, Joana Pauperio, Pedro Beja

42481C44-0513-51A4-8329-C0EC9988DA5F 10.3897/BDJ.12.e117169.suppl2 Supplementary material 2 IBI - Lepidoptera 08 library - Specimen details Data type Specimen data records Brief description The file includes information about all records in BOLD for the IBI - Lepidoptera 08 library. It contains collecting and identification data. The data are as downloaded from BOLD in the tsv format, without further processing. File: oo_985192.txt https://binary.pensoft.net/file/985192 Sónia Ferreira, Martin F. V. Corley, João Nunes, Jorge Rosete, Sasha Vasconcelos, Vanessa A. Mata, Teresa L Silva, Pedro Sousa, Rui Andrade, José Manuel Grosso Silva, Joana Pauperio, Pedro Beja

8562C2EF-3CA6-5085-9E1C-EEBFED3E3CB5 10.3897/BDJ.12.e117169.suppl3 Supplementary material 3 IBI - Lepidoptera 08 - Specimen details - Darwin Core Standard Data type Specimen data records in the Darwin Core Standard format Brief description The file includes information about all records in BOLD for the IBI - Lepidoptera 08 library. It contains collecting and identification data. The data are as downloaded from BOLD in the Darwin Core Standard format, without further processing. File: oo_985189.txt https://binary.pensoft.net/file/985189 Sónia Ferreira, Martin F. V. Corley, João Nunes, Jorge Rosete, Sasha Vasconcelos, Vanessa A. Mata, Teresa L Silva, Pedro Sousa, Rui Andrade, José Manuel Grosso Silva, Joana Pauperio, Pedro Beja

39222D6A-1546-5F7C-B0CF-93161B10A95E 10.3897/BDJ.12.e117169.suppl4 Supplementary material 4 IBI- Lepidoptera 08 library - DNA sequences Data type Specimen genomic data, DNA sequences Brief description COI sequences in fasta format. Each sequence is identified by the BOLDProcessID, species name, genetic marker name and GenBank accession number, all separated by a vertical bar. The data are as downloaded from BOLD. File: oo_985193.fas https://binary.pensoft.net/file/985193 Sónia Ferreira, Martin F. V. Corley, João Nunes, Jorge Rosete, Sasha Vasconcelos, Vanessa A. Mata, Teresa L Silva, Pedro Sousa, Rui Andrade, José Manuel GrossoSilva, Catarina J. Pinho, Cátia Chaves, Filipa MS Martins, Joana Pinto, Pamela Puppo, Antonio Muñoz-Mérida, John Archer, Joana Pauperio, Pedro Beja

AA8237F4-2CAA-561E-8318-7417634FA806 10.3897/BDJ.12.e117169.suppl5 Supplementary material 5 Phylogenetic tree (NJ) of all DNA barcodes used in the study generated in BOLD Systems Data type Phylogenetic tree Brief description Phylogenetic tree (NJ) of all the specimens DNA barcodes within DS-IBILP08: the IBI-Lepidoptera 08 dataset collected in continental Portugal, all of which have been morphologically identified to species level. File: oo_982594.pdf https://binary.pensoft.net/file/982594 Sónia Ferreira, Martin F. V. Corley, João Nunes, Jorge Rosete, Sasha Vasconcelos, Vanessa A. Mata, Teresa L Silva, Pedro Sousa, Rui Andrade, José Manuel Grosso Silva, Catarina J. Pinho, Cátia Chaves, Filipa MS Martins, Joana Pinto, Pamela Puppo, Antonio Muñoz-Mérida, John Archer, Joana Pauperio, Pedro Beja

9AA9F49F-96CF-52F0-BB9A-98AFD1C5D7BB 10.3897/BDJ.12.e117169.suppl6 Supplementary material 6 Specimens with occurence in continental Portugal missing DNA barcodes Data type Gap Checklist Brief description Specimens with occurrence in continental Portugal missing DNA barcodes. To each species is discriminated if the species has specimens in BOLD, but still has no DNA barcode or specimens are still needed. File: oo_961451.txt https://binary.pensoft.net/file/961451 Sónia Ferreira, Martin F. V. Corley, João Nunes, Jorge Rosete, Sasha Vasconcelos, Vanessa A. Mata, Teresa L Silva, Pedro Sousa, Rui Andrade, José Manuel Grosso Silva, Catarina J. Pinho, Cátia Chaves, Filipa MS Martins, Joana Pinto, Pamela Puppo, Antonio Muñoz-Mérida, John Archer, Joana Pauperio, Pedro Beja

## Figures and Tables

**Figure 1a.**
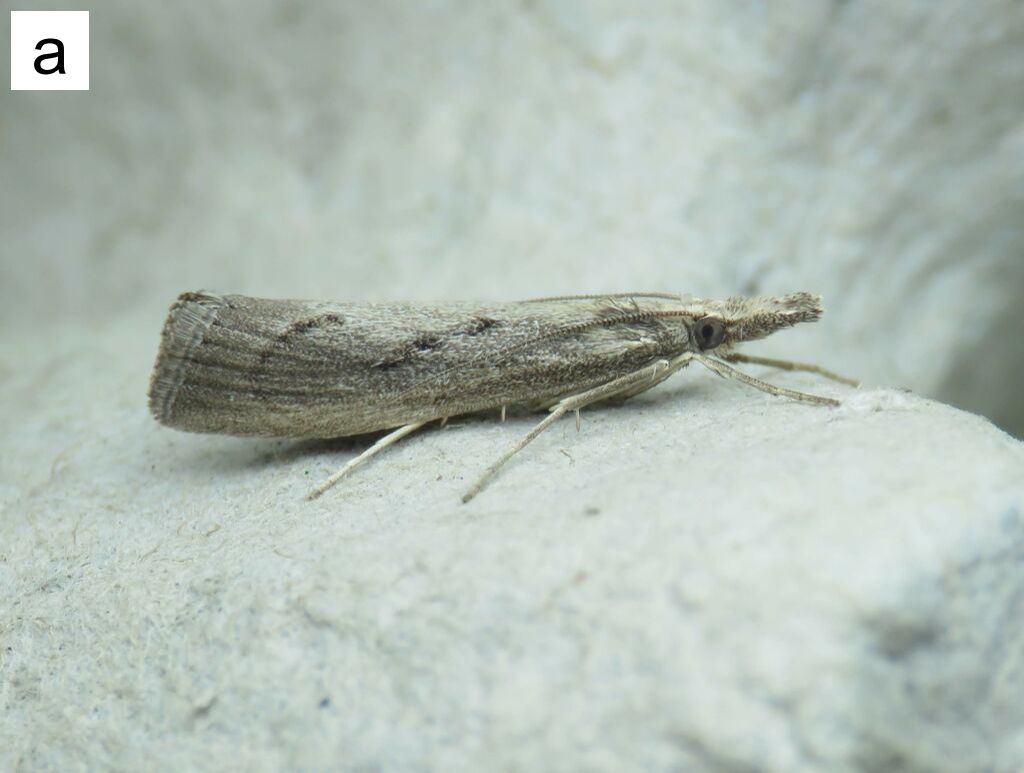
*Agriphilatersellus* Lederer, 1855, Crambidae - BIN URI BOLD:AEC8773;

**Figure 1b.**
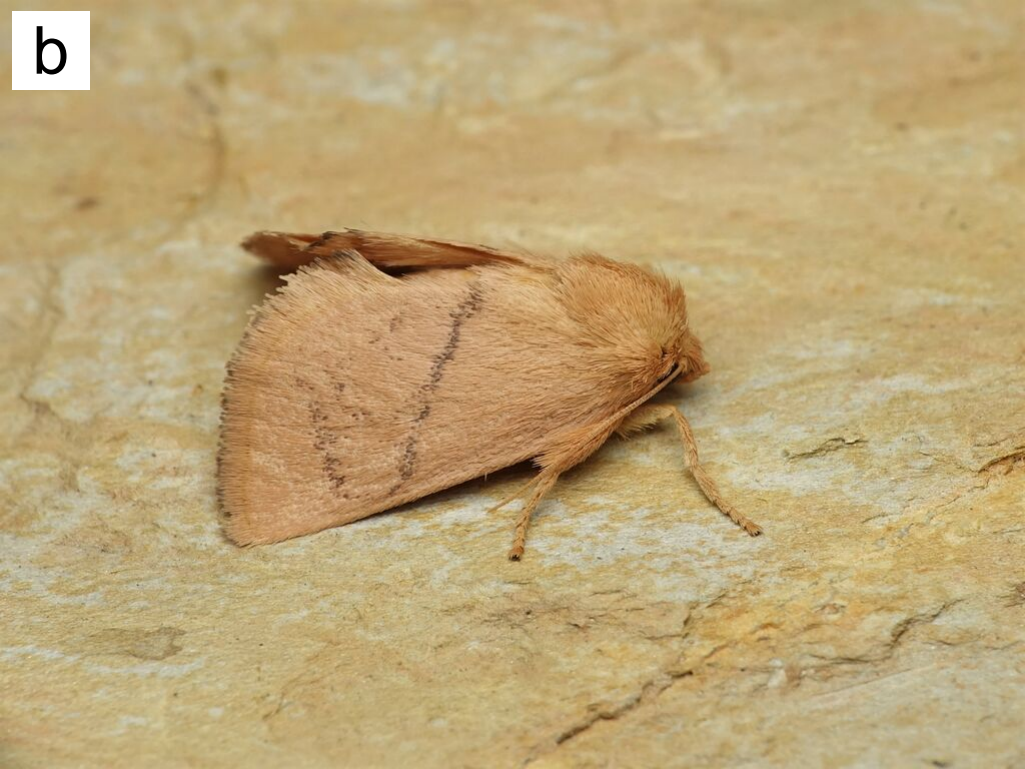
*Hoyosiacodeti* (Oberthür, 1883), Limacodidae - BIN URI BOLD:ADV3396;

**Figure 1c.**
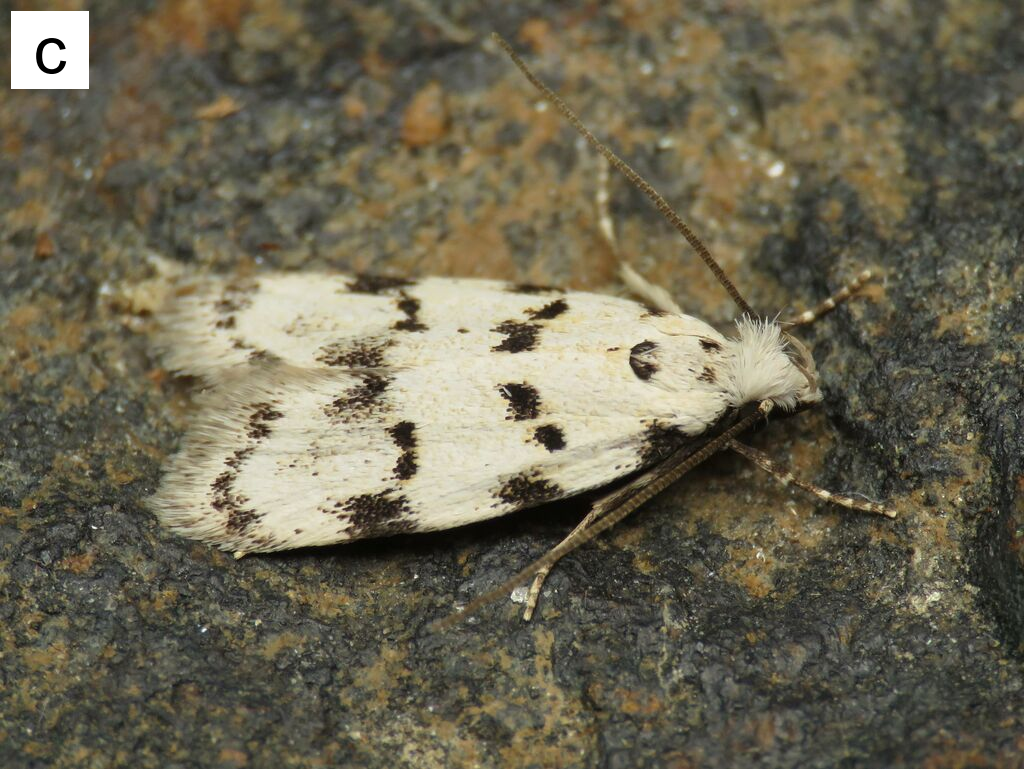
*Symmocaserrata* Gozmany, 1985, Autostichidae - BIN URI BOLD:AEO2252;

**Figure 1d.**
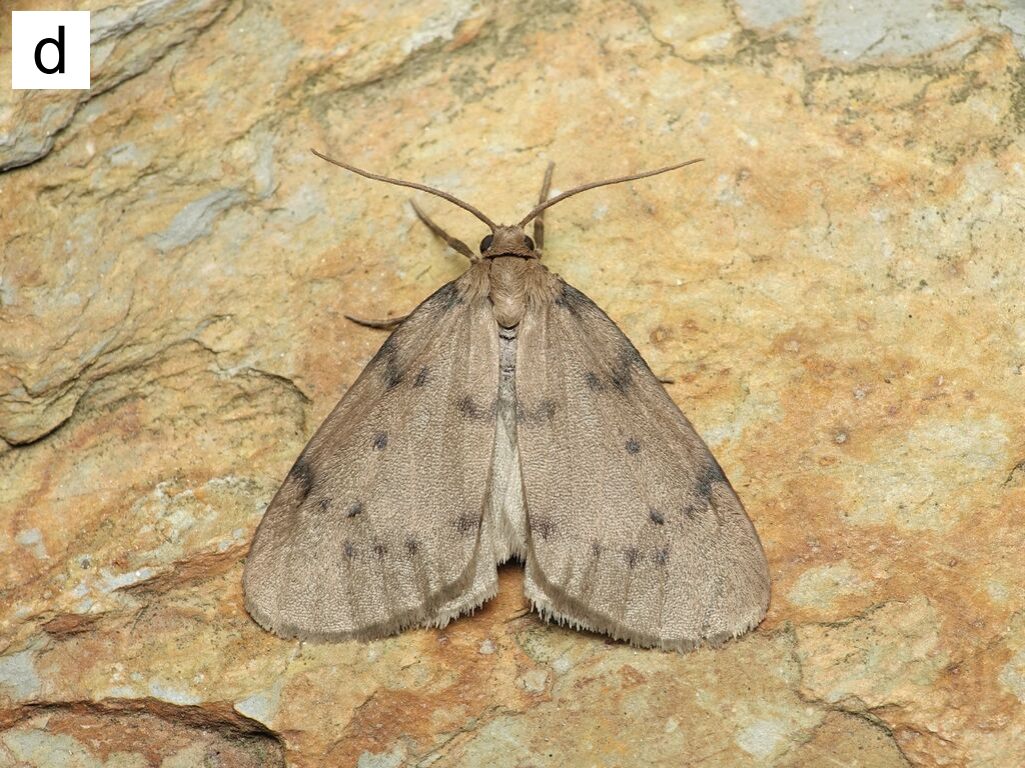
*Paidiarica* Freyer, 1855, Erebidae - BIN URI BOLD:AEC7499;

**Figure 1e.**
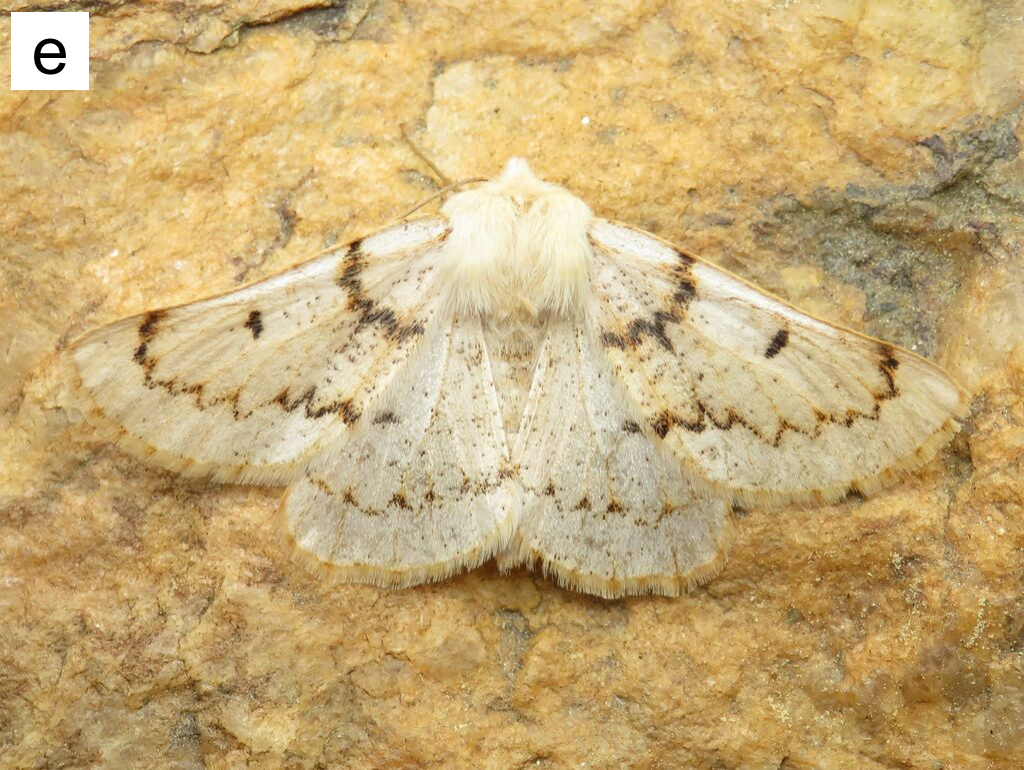
*Dysciadistinctaria* (Bang-Haas, 1910), Geometridae - BIN URI BOLD:ACF1137;

**Figure 1f.**
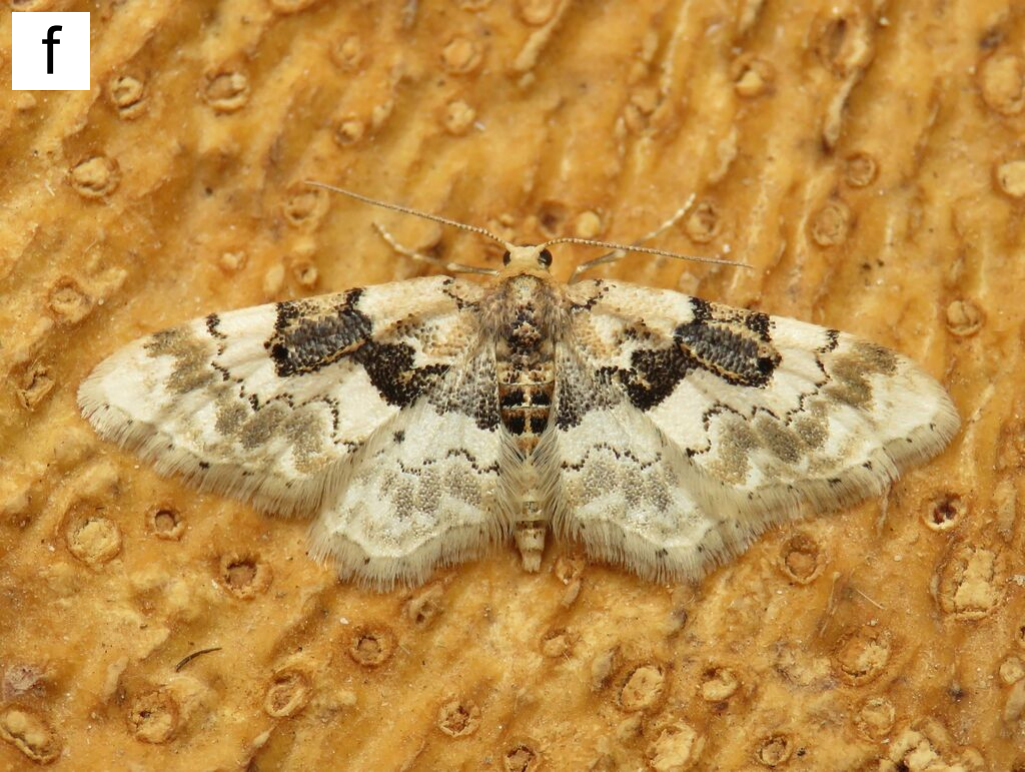
*Idaeafiguraria* (Bang-Haas, 1907), Geometridae - BIN URI BOLD:AEC6951.

**Figure 2a.**
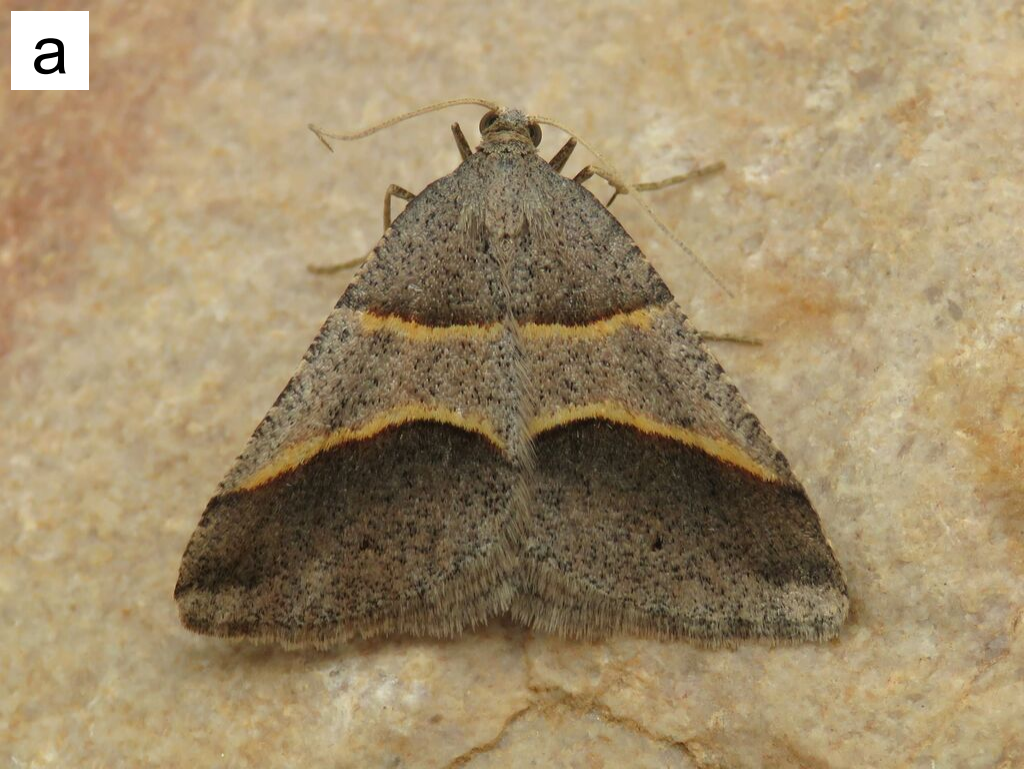
*Periguneconvergata* (Villers, 1789), Geometridae - BIN URI BOLD:AEH2380;

**Figure 2b.**
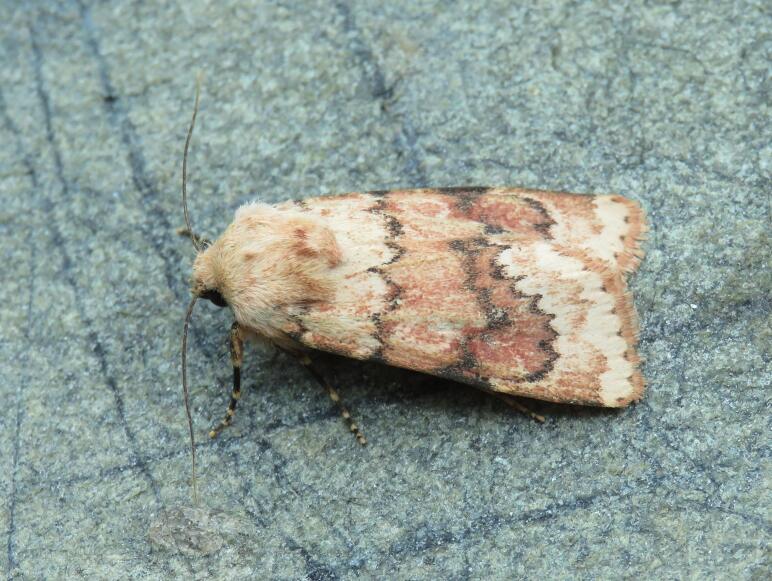
*Dichagyrisconstanti* Millière, 1860, Noctuidae - BIN URI BOLD:AED6734;

**Figure 2c.**
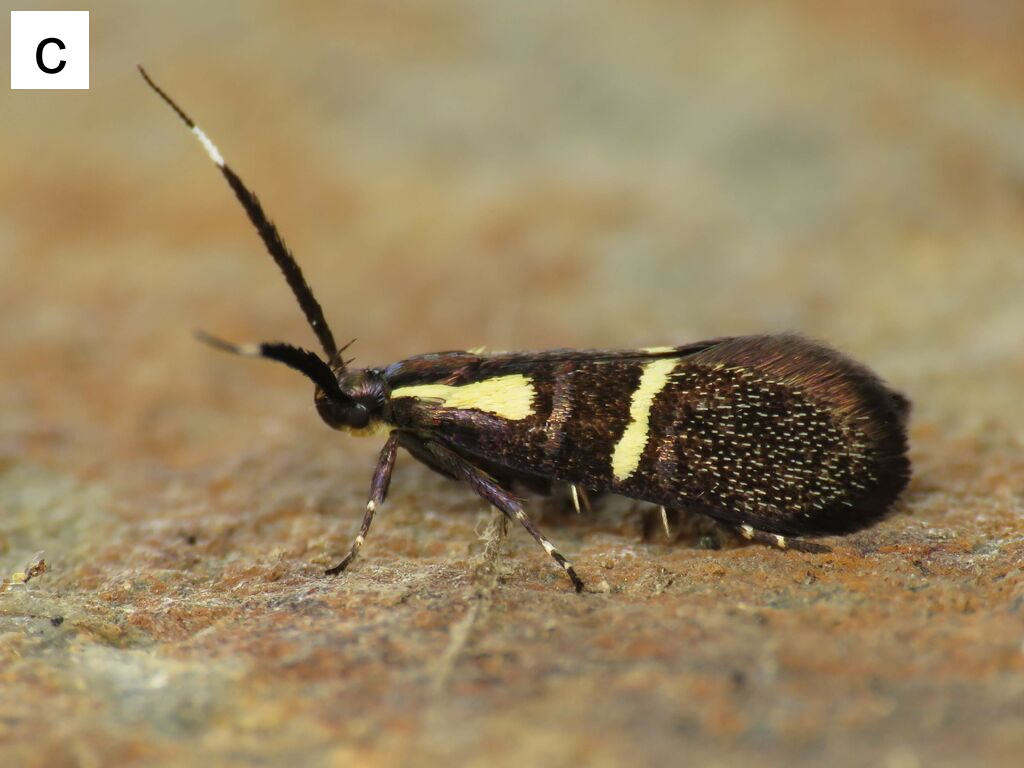
*Dasyceraoliviella* (Fabricius, 1794), Oecophoridae - BIN URI BOLD:ADT8549;

**Figure 2d.**
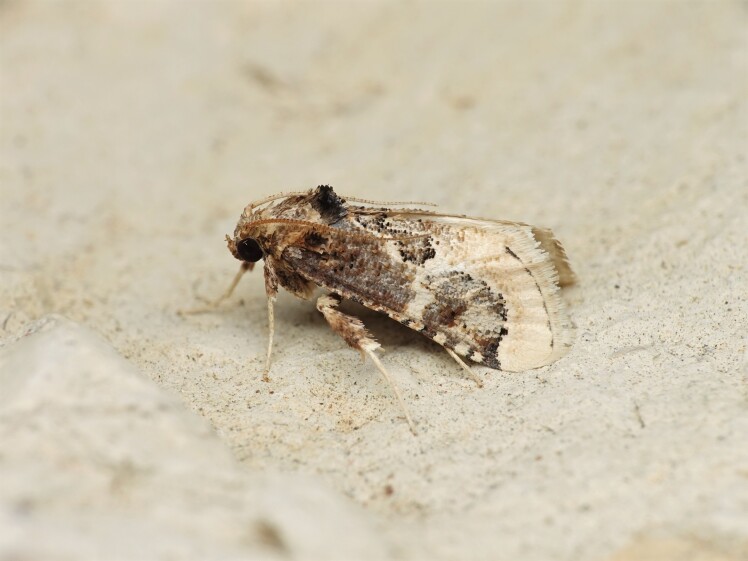
*Lorymaegregialis* (Herrich-Schäffer, 1838), Pyralidae - BIN URI BOLD:AEC8952;

**Figure 2e.**
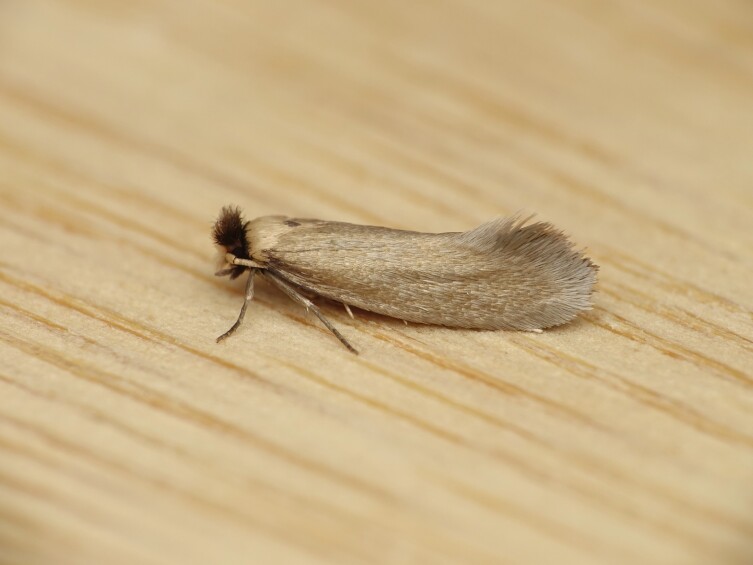
*Crassicornellaagenjoi* (Petersen, 1957), Tineidae - BIN URI BOLD:ADT5404;

**Figure 2f.**
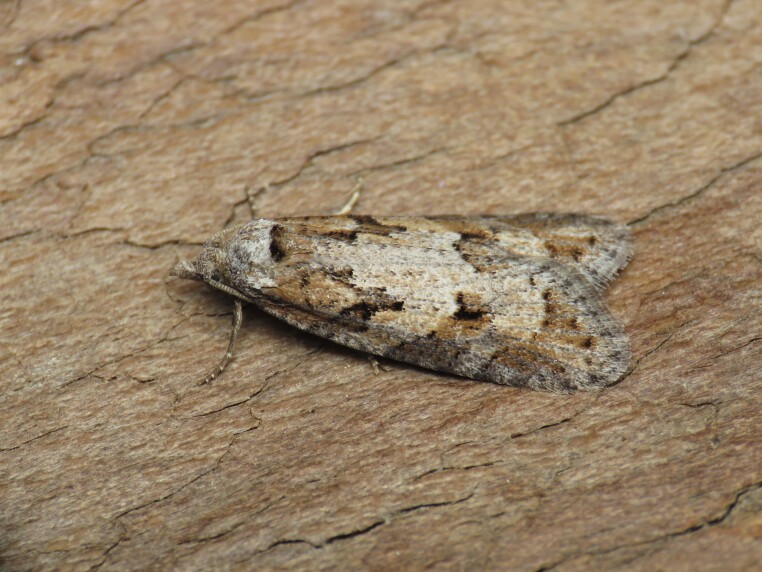
*Eananervana* (de Joannis, 1908), Tortricidae - BIN URI BOLD:ADS8408.
