# Supplementary material for: The InBIO Barcoding Initiative Database: DNA barcodes of Portuguese moths
Source: Biodivers Data J. 2024 May 16;12:e117169. doi: 10.3897/BDJ.12.e117169 (PMC11188589; doi:10.3897/BDJ.12.e117169)
Supplement: Supplementary material 5 — Phylogenetic tree (NJ) of all DNA barcodes used in the study generated in BOLD Systems [file bdj-12-e117169-s005.pdf]

BOLD TaxonID Tree

Title	:	Tree Result - DS-IBILP08 (2350 records selected)
Date	:	10-Feb-2024
Data Type	:	Nucleotide
Distance Model	:	Kimura 2 Parameter
Marker	:	COI-5P
Colourization	:	[blue]=Stop Codons [red]=Contamination or misidentification
Label	:	Sample ID
Label	:	Taxon
Label	:	Country
Label	:	Barcode Cluster (BIN)
Sequence Count	:	2350
Species count	:	1158
Genus count	:	594
Family count	:	51
Unidentified	:	0
BIN Count	:	1184

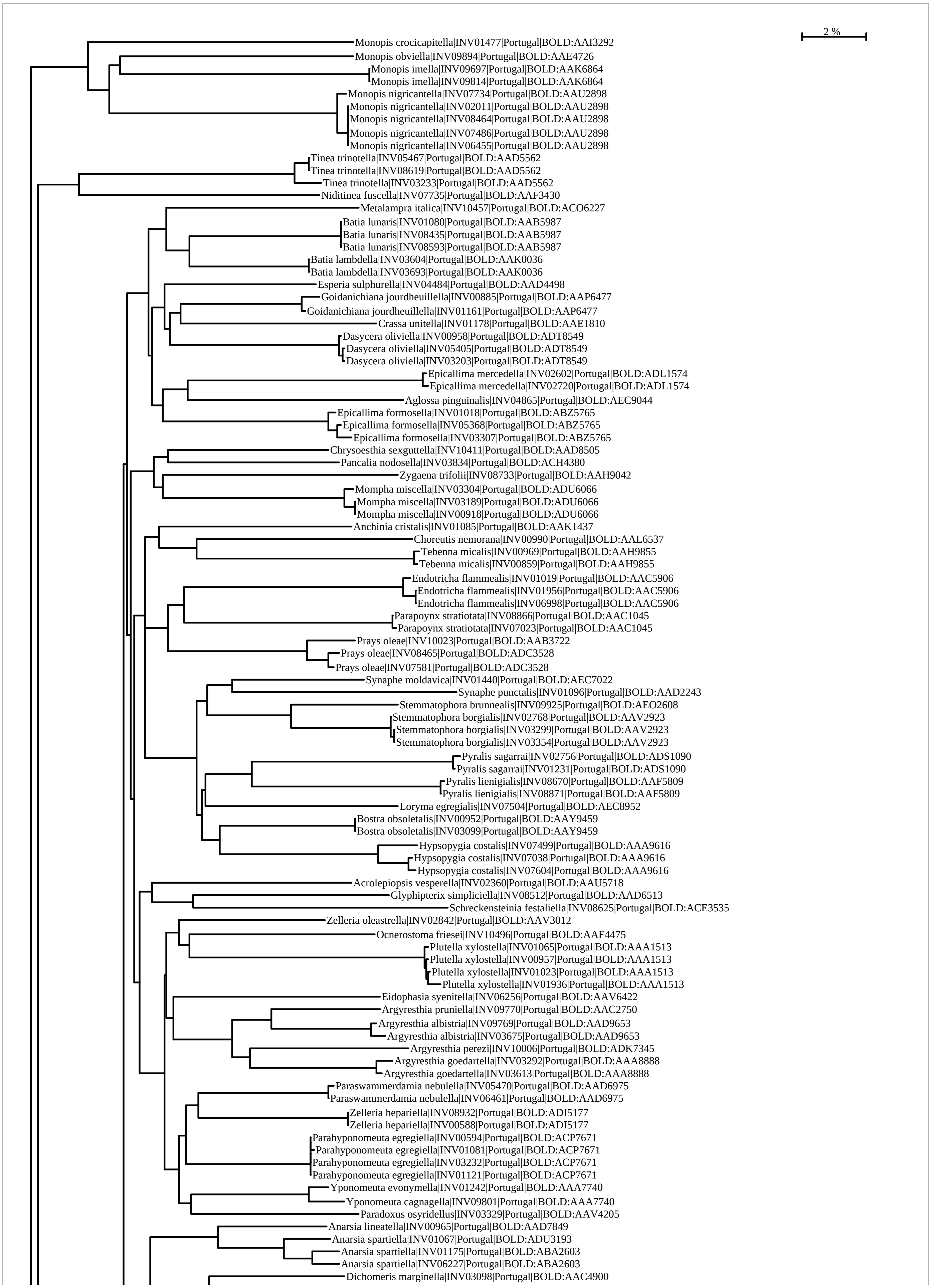
2 %
Monopis crocicapitella|INV01477|Portugal|BOLD:AAI3292
Monopis obviella|INV09894|Portugal|BOLD:AAE4726
Monopis imella|INV09697|Portugal|BOLD:AAK6864
Monopis imella|INV09814|Portugal|BOLD:AAK6864
Monopis nigricantella|INV07734|Portugal|BOLD:AAU2898
Monopis nigricantella|INV02011|Portugal|BOLD:AAU2898
Monopis nigricantella|INV08464|Portugal|BOLD:AAU2898
Monopis nigricantella|INV07486|Portugal|BOLD:AAU2898
Monopis nigricantella|INV06455|Portugal|BOLD:AAU2898
Tinea trinotella|INV05467|Portugal|BOLD:AAD5562
Tinea trinotella|INV08619|Portugal|BOLD:AAD5562
Tinea trinotella|INV03233|Portugal|BOLD:AAD5562
Niditinea fuscella|INV07735|Portugal|BOLD:AAF3430
Metalampra italica|INV10457|Portugal|BOLD:ACO6227
Batia lunaris|INV01080|Portugal|BOLD:AAB5987
Batia lunaris|INV08435|Portugal|BOLD:AAB5987
Batia lunaris|INV08593|Portugal|BOLD:AAB5987
Batia lambdella|INV03604|Portugal|BOLD:AAK0036
Batia lambdella|INV03693|Portugal|BOLD:AAK0036
Esperia sulphurella|INV04484|Portugal|BOLD:AAD4498
Goidanichiana jourdheuillella|INV00885|Portugal|BOLD:AAP6477
Goidanichiana jourdheuillella|INV01161|Portugal|BOLD:AAP6477
Crassa unitella|INV01178|Portugal|BOLD:AAE1810
Dasycera oliviella|INV00958|Portugal|BOLD:ADT8549
Dasycera oliviella|INV05405|Portugal|BOLD:ADT8549
Dasycera oliviella|INV03203|Portugal|BOLD:ADT8549
Epicallima mercedella|INV02602|Portugal|BOLD:ADL1574
Epicallima mercedella|INV02720|Portugal|BOLD:ADL1574
Aglossa pinguinalis|INV04865|Portugal|BOLD:AEC9044
Epicallima formosella|INV01018|Portugal|BOLD:ABZ5765
Epicallima formosella|INV05368|Portugal|BOLD:ABZ5765
Epicallima formosella|INV03307|Portugal|BOLD:ABZ5765
Chrysoesthia sexguttella|INV10411|Portugal|BOLD:AAD8505
Pancalia nodosella|INV03834|Portugal|BOLD:ACH4380
Zygaena trifolii|INV08733|Portugal|BOLD:AAH9042
Mompha miscella|INV03304|Portugal|BOLD:ADU6066
Mompha miscella|INV03189|Portugal|BOLD:ADU6066
Mompha miscella|INV00918|Portugal|BOLD:ADU6066
Anchinia cristalis|INV01085|Portugal|BOLD:AAK1437
Choreutis nemorana|INV00990|Portugal|BOLD:AAL6537
Tebenna micalis|INV00969|Portugal|BOLD:AAH9855
Tebenna micalis|INV00859|Portugal|BOLD:AAH9855
Endotricha flammealis|INV01019|Portugal|BOLD:AAC5906
Endotricha flammealis|INV01956|Portugal|BOLD:AAC5906
Endotricha flammealis|INV06998|Portugal|BOLD:AAC5906
Parapoynx stratiotata|INV08866|Portugal|BOLD:AAC1045
Parapoynx stratiotata|INV07023|Portugal|BOLD:AAC1045
Prays oleae|INV10023|Portugal|BOLD:AAB3722
Prays oleae|INV08465|Portugal|BOLD:ADC3528
Prays oleae|INV07581|Portugal|BOLD:ADC3528
Synaphe moldavica|INV01440|Portugal|BOLD:AEC7022
Synaphe punctalis|INV01096|Portugal|BOLD:AAD2243
Stemmatophora brunnealis|INV09925|Portugal|BOLD:AEO2608
Stemmatophora borgialis|INV02768|Portugal|BOLD:AAV2923
Stemmatophora borgialis|INV03299|Portugal|BOLD:AAV2923
Stemmatophora borgialis|INV03354|Portugal|BOLD:AAV2923
Pyralis sagarrai|INV02756|Portugal|BOLD:ADS1090
Pyralis sagarrai|INV01231|Portugal|BOLD:ADS1090
Pyralis lienigialis|INV08670|Portugal|BOLD:AAF5809
Pyralis lienigialis|INV08871|Portugal|BOLD:AAF5809
Loryma egregialis|INV07504|Portugal|BOLD:AEC8952
Bostra obsoletalis|INV00952|Portugal|BOLD:AAY9459
Bostra obsoletalis|INV03099|Portugal|BOLD:AAY9459
Hypsopygia costalis|INV07499|Portugal|BOLD:AAA9616
Hypsopygia costalis|INV07038|Portugal|BOLD:AAA9616
Hypsopygia costalis|INV07604|Portugal|BOLD:AAA9616
Acrolepiopsis vesperella|INV02360|Portugal|BOLD:AAU5718
Glyphipterix simpliciella|INV08512|Portugal|BOLD:AAD6513
Schreckensteinia festaliella|INV08625|Portugal|BOLD:ACE3535
Zelleria oleastrella|INV02842|Portugal|BOLD:AAV3012
Ocnerostoma friesei|INV10496|Portugal|BOLD:AAF4475
Plutella xylostella|INV01065|Portugal|BOLD:AAA1513
Plutella xylostella|INV00957|Portugal|BOLD:AAA1513
Plutella xylostella|INV01023|Portugal|BOLD:AAA1513
Plutella xylostella|INV01936|Portugal|BOLD:AAA1513
Eidophasia syenitella|INV06256|Portugal|BOLD:AAV6422
Argyresthia pruniella|INV09770|Portugal|BOLD:AAC2750
Argyresthia albistria|INV09769|Portugal|BOLD:AAD9653
Argyresthia albistria|INV03675|Portugal|BOLD:AAD9653
Argyresthia perezi|INV10006|Portugal|BOLD:ADK7345
Argyresthia goedartella|INV03292|Portugal|BOLD:AAA8888
Argyresthia goedartella|INV03613|Portugal|BOLD:AAA8888
Paraswammerdamia nebulella|INV05470|Portugal|BOLD:AAD6975
Paraswammerdamia nebulella|INV06461|Portugal|BOLD:AAD6975
Zelleria hepariella|INV08932|Portugal|BOLD:ADI5177
Zelleria hepariella|INV00588|Portugal|BOLD:ADI5177
Parahyponomeuta egregiella|INV00594|Portugal|BOLD:ACP7671
Parahyponomeuta egregiella|INV01081|Portugal|BOLD:ACP7671
Parahyponomeuta egregiella|INV03232|Portugal|BOLD:ACP7671
Parahyponomeuta egregiella|INV01121|Portugal|BOLD:ACP7671
Yponomeuta evonymella|INV01242|Portugal|BOLD:AAA7740
Yponomeuta cagnagella|INV09801|Portugal|BOLD:AAA7740
Paradoxus osyridellus|INV03329|Portugal|BOLD:AAV4205
Anarsia lineatella|INV00965|Portugal|BOLD:AAD7849
Anarsia spartiella|INV01067|Portugal|BOLD:ADU3193
Anarsia spartiella|INV01175|Portugal|BOLD:ABA2603
Anarsia spartiella|INV06227|Portugal|BOLD:ABA2603
Dichomeris marginella|INV03098|Portugal|BOLD:AAC4900

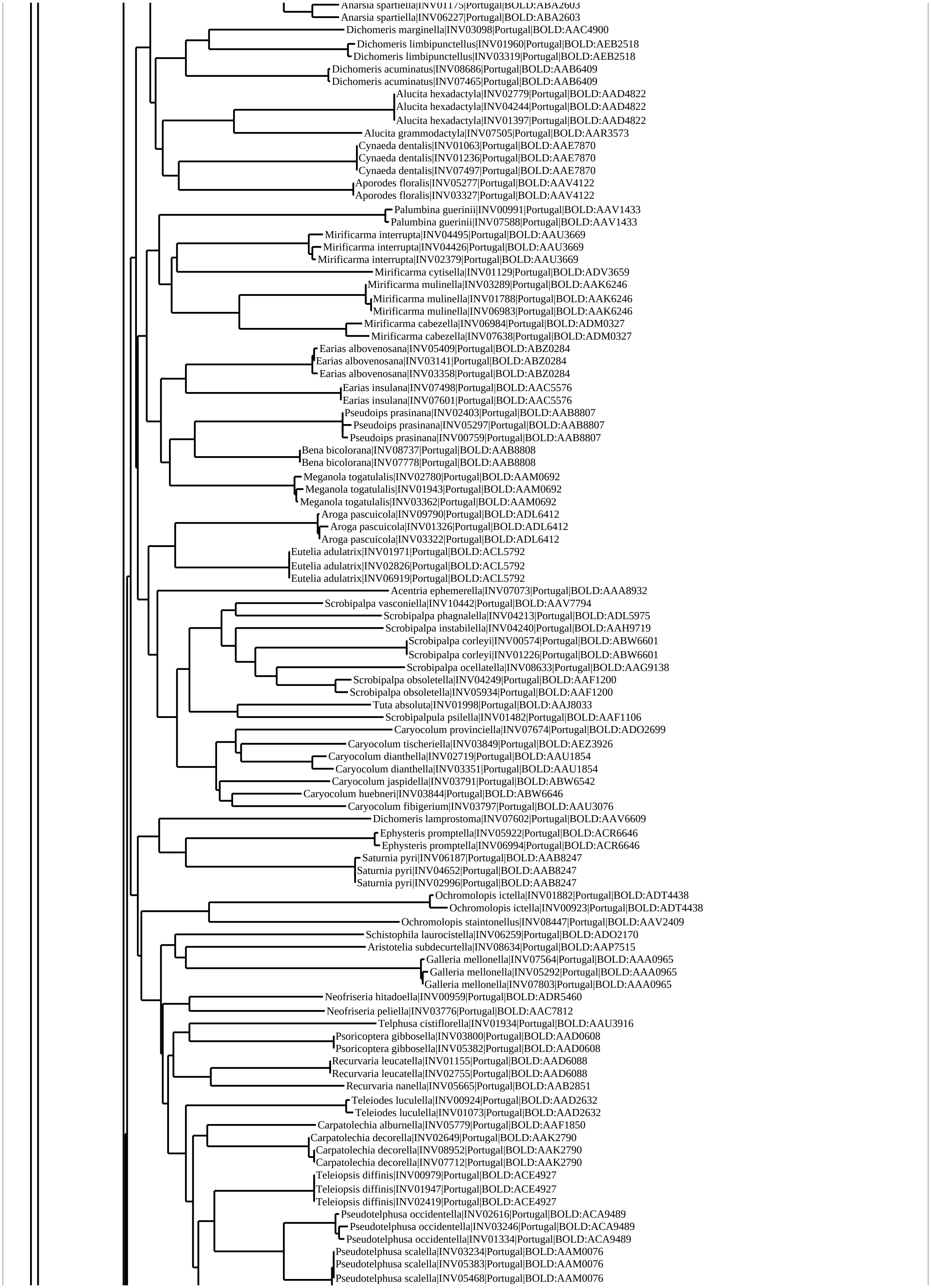
Anarsia spartiella|INV01175|Portugal|BOLD:ABA2603
Anarsia spartiella|INV06227|Portugal|BOLD:ABA2603
Dichomeris marginella|INV03098|Portugal|BOLD:AAC4900
Dichomeris limbipunctellus|INV01960|Portugal|BOLD:AEB2518
Dichomeris limbipunctellus|INV03319|Portugal|BOLD:AEB2518
Dichomeris acuminatus|INV08686|Portugal|BOLD:AAB6409
Dichomeris acuminatus|INV07465|Portugal|BOLD:AAB6409
Alucita hexadactyla|INV02779|Portugal|BOLD:AAD4822
Alucita hexadactyla|INV04244|Portugal|BOLD:AAD4822
Alucita hexadactyla|INV01397|Portugal|BOLD:AAD4822
Alucita grammodactyla|INV07505|Portugal|BOLD:AAR3573
Cynaeda dentalis|INV01063|Portugal|BOLD:AAE7870
Cynaeda dentalis|INV01236|Portugal|BOLD:AAE7870
Cynaeda dentalis|INV07497|Portugal|BOLD:AAE7870
Aporodes floralis|INV05277|Portugal|BOLD:AAV4122
Aporodes floralis|INV03327|Portugal|BOLD:AAV4122
Palumbina guerinii|INV00991|Portugal|BOLD:AAV1433
Palumbina guerinii|INV07588|Portugal|BOLD:AAV1433
Mirificarma interrupta|INV04495|Portugal|BOLD:AAU3669
Mirificarma interrupta|INV04426|Portugal|BOLD:AAU3669
Mirificarma interrupta|INV02379|Portugal|BOLD:AAU3669
Mirificarma cytisella|INV01129|Portugal|BOLD:ADV3659
Mirificarma mulinella|INV03289|Portugal|BOLD:AAK6246
Mirificarma mulinella|INV01788|Portugal|BOLD:AAK6246
Mirificarma mulinella|INV06983|Portugal|BOLD:AAK6246
Mirificarma cabezella|INV06984|Portugal|BOLD:ADM0327
Mirificarma cabezella|INV07638|Portugal|BOLD:ADM0327
Earias albovenosana|INV05409|Portugal|BOLD:ABZ0284
Earias albovenosana|INV03141|Portugal|BOLD:ABZ0284
Earias albovenosana|INV03358|Portugal|BOLD:ABZ0284
Earias insulana|INV07498|Portugal|BOLD:AAC5576
Earias insulana|INV07601|Portugal|BOLD:AAC5576
Pseudoips prasinana|INV02403|Portugal|BOLD:AAB8807
Pseudoips prasinana|INV05297|Portugal|BOLD:AAB8807
Pseudoips prasinana|INV00759|Portugal|BOLD:AAB8807
Bena bicolorana|INV08737|Portugal|BOLD:AAB8808
Bena bicolorana|INV07778|Portugal|BOLD:AAB8808
Meganola togatulalis|INV02780|Portugal|BOLD:AAM0692
Meganola togatulalis|INV01943|Portugal|BOLD:AAM0692
Meganola togatulalis|INV03362|Portugal|BOLD:AAM0692
Aroga pascuicola|INV09790|Portugal|BOLD:ADL6412
Aroga pascuicola|INV01326|Portugal|BOLD:ADL6412
Aroga pascuicola|INV03322|Portugal|BOLD:ADL6412
Eutelia adulatrix|INV01971|Portugal|BOLD:ACL5792
Eutelia adulatrix|INV02826|Portugal|BOLD:ACL5792
Eutelia adulatrix|INV06919|Portugal|BOLD:ACL5792
Acentria ephemerella|INV07073|Portugal|BOLD:AAA8932
Scrobipalpa vasconiella|INV10442|Portugal|BOLD:AAV7794
Scrobipalpa phagnalella|INV04213|Portugal|BOLD:ADL5975
Scrobipalpa instabilella|INV04240|Portugal|BOLD:AAH9719
Scrobipalpa corleyi|INV00574|Portugal|BOLD:ABW6601
Scrobipalpa corleyi|INV01226|Portugal|BOLD:ABW6601
Scrobipalpa ocellatella|INV08633|Portugal|BOLD:AAG9138
Scrobipalpa obsoletella|INV04249|Portugal|BOLD:AAF1200
Scrobipalpa obsoletella|INV05934|Portugal|BOLD:AAF1200
Tuta absoluta|INV01998|Portugal|BOLD:AAJ8033
Scrobipalpula psilella|INV01482|Portugal|BOLD:AAF1106
Caryocolum provinciella|INV07674|Portugal|BOLD:ADO2699
Caryocolum tischeriella|INV03849|Portugal|BOLD:AEZ3926
Caryocolum dianthella|INV02719|Portugal|BOLD:AAU1854
Caryocolum dianthella|INV03351|Portugal|BOLD:AAU1854
Caryocolum jaspidella|INV03791|Portugal|BOLD:ABW6542
Caryocolum huebneri|INV03844|Portugal|BOLD:ABW6646
Caryocolum fibigerium|INV03797|Portugal|BOLD:AAU3076
Dichomeris lamprostoma|INV07602|Portugal|BOLD:AAV6609
Ephysteris promptella|INV05922|Portugal|BOLD:ACR6646
Ephysteris promptella|INV06994|Portugal|BOLD:ACR6646
Saturnia pyri|INV06187|Portugal|BOLD:AAB8247
Saturnia pyri|INV04652|Portugal|BOLD:AAB8247
Saturnia pyri|INV02996|Portugal|BOLD:AAB8247
Ochromolopis ictella|INV01882|Portugal|BOLD:ADT4438
Ochromolopis ictella|INV00923|Portugal|BOLD:ADT4438
Ochromolopis staintonellus|INV08447|Portugal|BOLD:AAV2409
Schistophila laurocistella|INV06259|Portugal|BOLD:ADO2170
Aristotelia subdecurtella|INV08634|Portugal|BOLD:AAP7515
Galleria mellonella|INV07564|Portugal|BOLD:AAA0965
Galleria mellonella|INV05292|Portugal|BOLD:AAA0965
Galleria mellonella|INV07803|Portugal|BOLD:AAA0965
Neofriseria hitadoella|INV00959|Portugal|BOLD:ADR5460
Neofriseria peliella|INV03776|Portugal|BOLD:AAC7812
Telphusa cistiflorella|INV01934|Portugal|BOLD:AAU3916
Psoricoptera gibbosella|INV03800|Portugal|BOLD:AAD0608
Psoricoptera gibbosella|INV05382|Portugal|BOLD:AAD0608
Recurvaria leucatella|INV01155|Portugal|BOLD:AAD6088
Recurvaria leucatella|INV02755|Portugal|BOLD:AAD6088
Recurvaria nanella|INV05665|Portugal|BOLD:AAB2851
Teleiodes luculella|INV00924|Portugal|BOLD:AAD2632
Teleiodes luculella|INV01073|Portugal|BOLD:AAD2632
Carpatolechia alburnella|INV05779|Portugal|BOLD:AAF1850
Carpatolechia decorella|INV02649|Portugal|BOLD:AAK2790
Carpatolechia decorella|INV08952|Portugal|BOLD:AAK2790
Carpatolechia decorella|INV07712|Portugal|BOLD:AAK2790
Teleiopsis diffinis|INV00979|Portugal|BOLD:ACE4927
Teleiopsis diffinis|INV01947|Portugal|BOLD:ACE4927
Teleiopsis diffinis|INV02419|Portugal|BOLD:ACE4927
Pseudotelphusa occidentella|INV02616|Portugal|BOLD:ACA9489
Pseudotelphusa occidentella|INV03246|Portugal|BOLD:ACA9489
Pseudotelphusa occidentella|INV01334|Portugal|BOLD:ACA9489
Pseudotelphusa scalella|INV03234|Portugal|BOLD:AAM0076
Pseudotelphusa scalella|INV05383|Portugal|BOLD:AAM0076
Pseudotelphusa scalella|INV05468|Portugal|BOLD:AAM0076

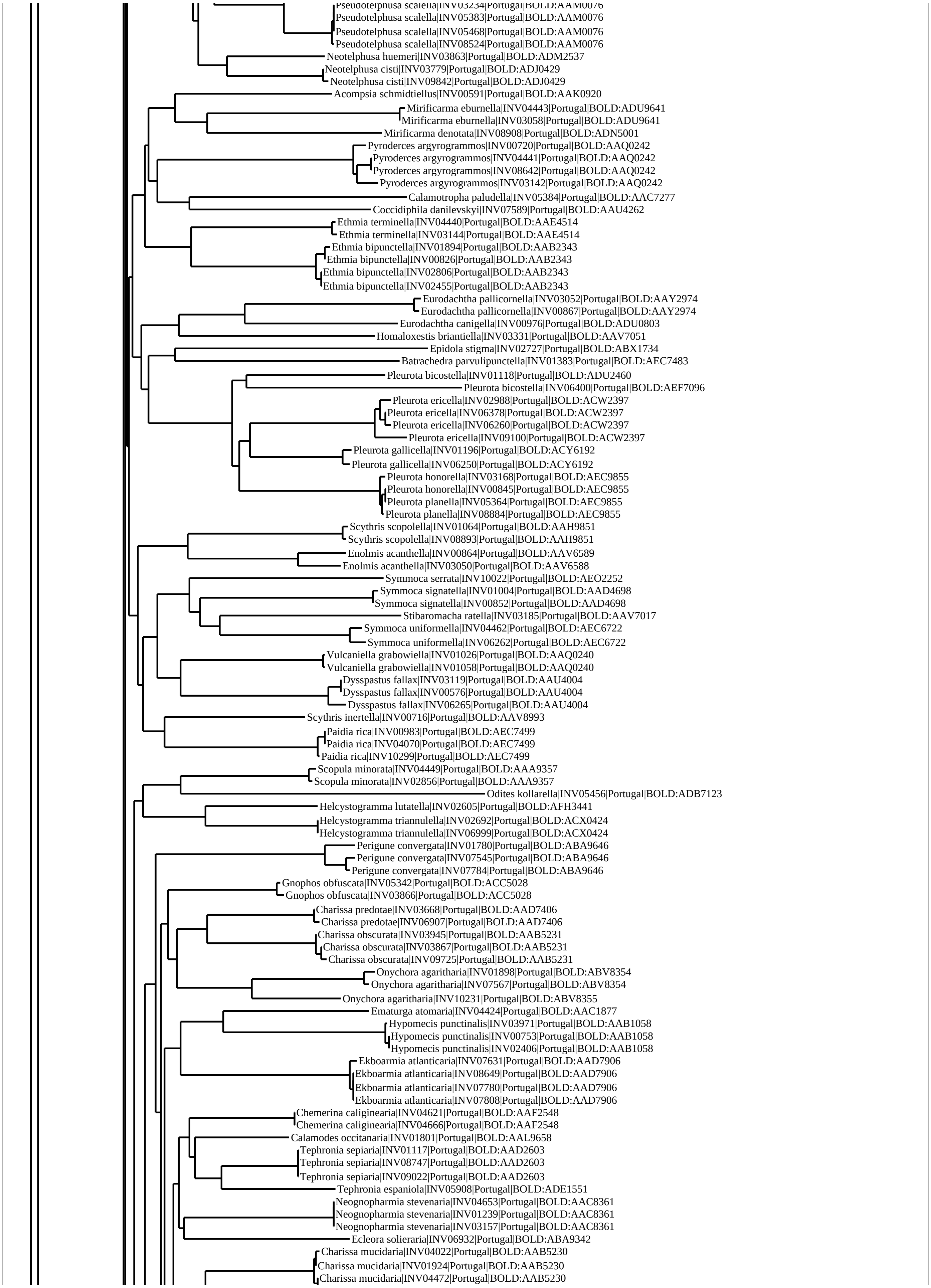
Pseudotelphusa scalella|INV03234|Portugal|BOLD:AAM0076
Pseudotelphusa scalella|INV05383|Portugal|BOLD:AAM0076
Pseudotelphusa scalella|INV05468|Portugal|BOLD:AAM0076
Pseudotelphusa scalella|INV08524|Portugal|BOLD:AAM0076
Neotelphusa huemeri|INV03863|Portugal|BOLD:ADM2537
Neotelphusa cisti|INV03779|Portugal|BOLD:ADJ0429
Neotelphusa cisti|INV09842|Portugal|BOLD:ADJ0429
Acompsia schmidtiellus|INV00591|Portugal|BOLD:AAK0920
Mirificarma eburnella|INV04443|Portugal|BOLD:ADU9641
Mirificarma eburnella|INV03058|Portugal|BOLD:ADU9641
Mirificarma denotata|INV08908|Portugal|BOLD:ADN5001
Pyroderces argyrogrammos|INV00720|Portugal|BOLD:AAQ0242
Pyroderces argyrogrammos|INV04441|Portugal|BOLD:AAQ0242
Pyroderces argyrogrammos|INV08642|Portugal|BOLD:AAQ0242
Pyroderces argyrogrammos|INV03142|Portugal|BOLD:AAQ0242
Calamotropha paludella|INV05384|Portugal|BOLD:AAC7277
Coccidiphila danilevskyi|INV07589|Portugal|BOLD:AAU4262
Ethmia terminella|INV04440|Portugal|BOLD:AAE4514
Ethmia terminella|INV03144|Portugal|BOLD:AAE4514
Ethmia bipunctella|INV01894|Portugal|BOLD:AAB2343
Ethmia bipunctella|INV00826|Portugal|BOLD:AAB2343
Ethmia bipunctella|INV02806|Portugal|BOLD:AAB2343
Ethmia bipunctella|INV02455|Portugal|BOLD:AAB2343
Eurodachtha pallicornella|INV03052|Portugal|BOLD:AAY2974
Eurodachtha pallicornella|INV00867|Portugal|BOLD:AAY2974
Eurodachtha canigella|INV00976|Portugal|BOLD:ADU0803
Homaloxestis briantiella|INV03331|Portugal|BOLD:AAV7051
Epidola stigma|INV02727|Portugal|BOLD:ABX1734
Batrachedra parvulipunctella|INV01383|Portugal|BOLD:AEC7483
Pleurota bicostella|INV01118|Portugal|BOLD:ADU2460
Pleurota bicostella|INV06400|Portugal|BOLD:AEF7096
Pleurota ericella|INV02988|Portugal|BOLD:ACW2397
Pleurota ericella|INV06378|Portugal|BOLD:ACW2397
Pleurota ericella|INV06260|Portugal|BOLD:ACW2397
Pleurota ericella|INV09100|Portugal|BOLD:ACW2397
Pleurota gallicella|INV01196|Portugal|BOLD:ACY6192
Pleurota gallicella|INV06250|Portugal|BOLD:ACY6192
Pleurota honorella|INV03168|Portugal|BOLD:AEC9855
Pleurota honorella|INV00845|Portugal|BOLD:AEC9855
Pleurota planella|INV05364|Portugal|BOLD:AEC9855
Pleurota planella|INV08884|Portugal|BOLD:AEC9855
Scythris scopolella|INV01064|Portugal|BOLD:AAH9851
Scythris scopolella|INV08893|Portugal|BOLD:AAH9851
Enolmis acanthella|INV00864|Portugal|BOLD:AAV6589
Enolmis acanthella|INV03050|Portugal|BOLD:AAV6588
Symmoca serrata|INV10022|Portugal|BOLD:AEO2252
Symmoca signatella|INV01004|Portugal|BOLD:AAD4698
Symmoca signatella|INV00852|Portugal|BOLD:AAD4698
Stibaromacha ratella|INV03185|Portugal|BOLD:AAV7017
Symmoca uniformella|INV04462|Portugal|BOLD:AEC6722
Symmoca uniformella|INV06262|Portugal|BOLD:AEC6722
Vulcaniella grabowiella|INV01026|Portugal|BOLD:AAQ0240
Vulcaniella grabowiella|INV01058|Portugal|BOLD:AAQ0240
Dysspastus fallax|INV03119|Portugal|BOLD:AAU4004
Dysspastus fallax|INV00576|Portugal|BOLD:AAU4004
Dysspastus fallax|INV06265|Portugal|BOLD:AAU4004
Scythris inertella|INV00716|Portugal|BOLD:AAV8993
Paidia rica|INV00983|Portugal|BOLD:AEC7499
Paidia rica|INV04070|Portugal|BOLD:AEC7499
Paidia rica|INV10299|Portugal|BOLD:AEC7499
Scopula minorata|INV04449|Portugal|BOLD:AAA9357
Scopula minorata|INV02856|Portugal|BOLD:AAA9357
Odites kollarella|INV05456|Portugal|BOLD:ADB7123
Helcystogramma lutatella|INV02605|Portugal|BOLD:AFH3441
Helcystogramma triannulella|INV02692|Portugal|BOLD:ACX0424
Helcystogramma triannulella|INV06999|Portugal|BOLD:ACX0424
Perigune convergata|INV01780|Portugal|BOLD:ABA9646
Perigune convergata|INV07545|Portugal|BOLD:ABA9646
Perigune convergata|INV07784|Portugal|BOLD:ABA9646
Gnophos obfuscata|INV05342|Portugal|BOLD:ACC5028
Gnophos obfuscata|INV03866|Portugal|BOLD:ACC5028
Charissa predotae|INV03668|Portugal|BOLD:AAD7406
Charissa predotae|INV06907|Portugal|BOLD:AAD7406
Charissa obscurata|INV03945|Portugal|BOLD:AAB5231
Charissa obscurata|INV03867|Portugal|BOLD:AAB5231
Charissa obscurata|INV09725|Portugal|BOLD:AAB5231
Onychora agaritharia|INV01898|Portugal|BOLD:ABV8354
Onychora agaritharia|INV07567|Portugal|BOLD:ABV8354
Onychora agaritharia|INV10231|Portugal|BOLD:ABV8355
Ematurga atomaria|INV04424|Portugal|BOLD:AAC1877
Hypomecis punctinalis|INV03971|Portugal|BOLD:AAB1058
Hypomecis punctinalis|INV00753|Portugal|BOLD:AAB1058
Hypomecis punctinalis|INV02406|Portugal|BOLD:AAB1058
Ekboarmia atlanticaria|INV07631|Portugal|BOLD:AAD7906
Ekboarmia atlanticaria|INV08649|Portugal|BOLD:AAD7906
Ekboarmia atlanticaria|INV07780|Portugal|BOLD:AAD7906
Ekboarmia atlanticaria|INV07808|Portugal|BOLD:AAD7906
Chemerina caliginearia|INV04621|Portugal|BOLD:AAF2548
Chemerina caliginearia|INV04666|Portugal|BOLD:AAF2548
Calamodes occitanaria|INV01801|Portugal|BOLD:AAL9658
Tephronia sepiaria|INV01117|Portugal|BOLD:AAD2603
Tephronia sepiaria|INV08747|Portugal|BOLD:AAD2603
Tephronia sepiaria|INV09022|Portugal|BOLD:AAD2603
Tephronia espaniola|INV05908|Portugal|BOLD:ADE1551
Neognopharmia stevenaria|INV04653|Portugal|BOLD:AAC8361
Neognopharmia stevenaria|INV01239|Portugal|BOLD:AAC8361
Neognopharmia stevenaria|INV03157|Portugal|BOLD:AAC8361
Ecleora solieraria|INV06932|Portugal|BOLD:ABA9342
Charissa mucidaria|INV04022|Portugal|BOLD:AAB5230
Charissa mucidaria|INV01924|Portugal|BOLD:AAB5230
Charissa mucidaria|INV04472|Portugal|BOLD:AAB5230

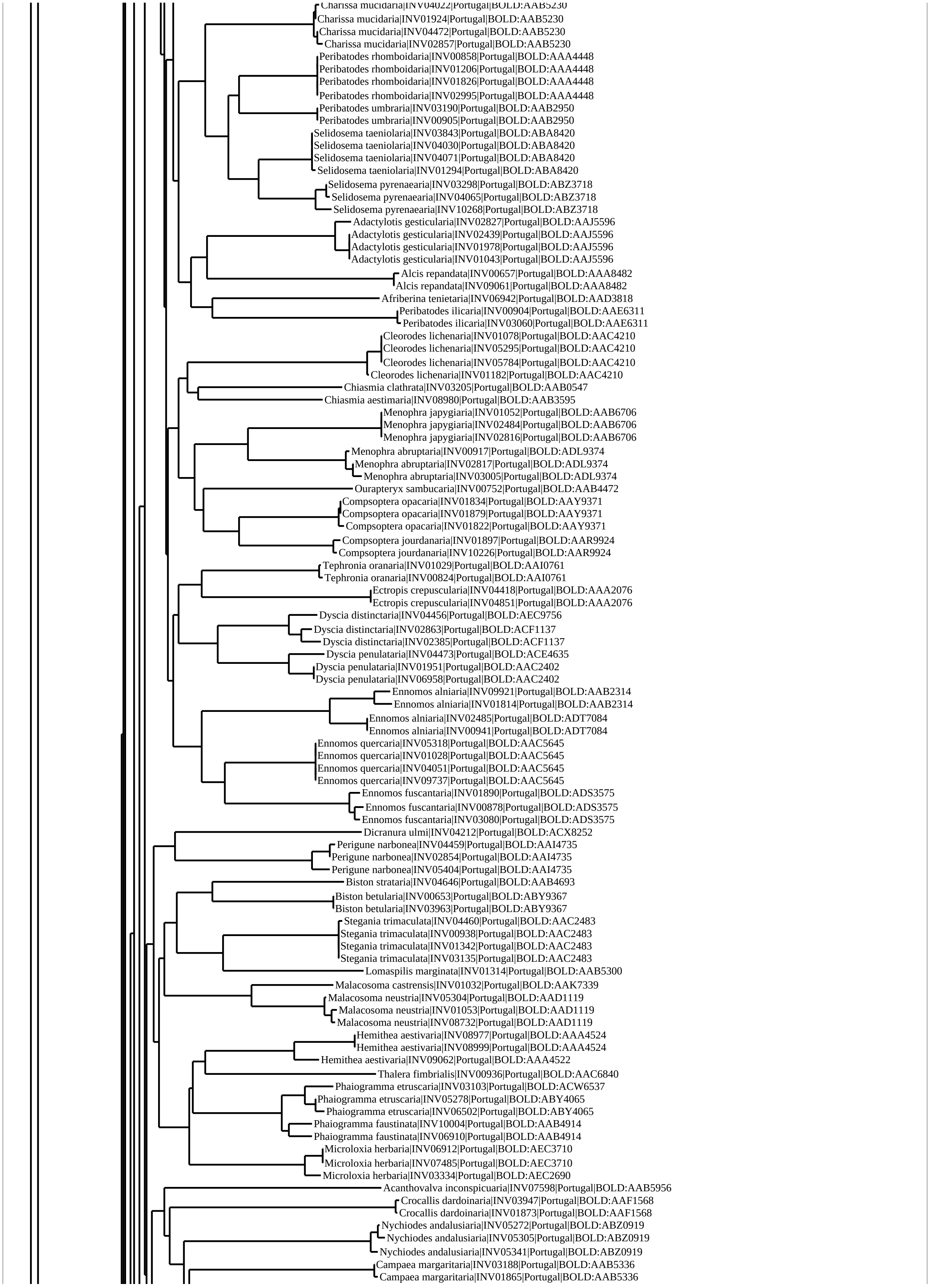
Charissa mucidaria|INV04022|Portugal|BOLD:AAB5230
Charissa mucidaria|INV01924|Portugal|BOLD:AAB5230
Charissa mucidaria|INV04472|Portugal|BOLD:AAB5230
Charissa mucidaria|INV02857|Portugal|BOLD:AAB5230
Peribatodes rhomboidaria|INV00858|Portugal|BOLD:AAA4448
Peribatodes rhomboidaria|INV01206|Portugal|BOLD:AAA4448
Peribatodes rhomboidaria|INV01826|Portugal|BOLD:AAA4448
Peribatodes rhomboidaria|INV02995|Portugal|BOLD:AAA4448
Peribatodes umbraria|INV03190|Portugal|BOLD:AAB2950
Peribatodes umbraria|INV00905|Portugal|BOLD:AAB2950
Selidosema taeniolaria|INV03843|Portugal|BOLD:ABA8420
Selidosema taeniolaria|INV04030|Portugal|BOLD:ABA8420
Selidosema taeniolaria|INV04071|Portugal|BOLD:ABA8420
Selidosema taeniolaria|INV01294|Portugal|BOLD:ABA8420
Selidosema pyrenaearia|INV03298|Portugal|BOLD:ABZ3718
Selidosema pyrenaearia|INV04065|Portugal|BOLD:ABZ3718
Selidosema pyrenaearia|INV10268|Portugal|BOLD:ABZ3718
Adactylotis gesticularia|INV02827|Portugal|BOLD:AAJ5596
Adactylotis gesticularia|INV02439|Portugal|BOLD:AAJ5596
Adactylotis gesticularia|INV01978|Portugal|BOLD:AAJ5596
Adactylotis gesticularia|INV01043|Portugal|BOLD:AAJ5596
Alcis repandata|INV00657|Portugal|BOLD:AAA8482
Alcis repandata|INV09061|Portugal|BOLD:AAA8482
Afriberina tenietaria|INV06942|Portugal|BOLD:AAD3818
Peribatodes ilicaria|INV00904|Portugal|BOLD:AAE6311
Peribatodes ilicaria|INV03060|Portugal|BOLD:AAE6311
Cleorodes lichenaria|INV01078|Portugal|BOLD:AAC4210
Cleorodes lichenaria|INV05295|Portugal|BOLD:AAC4210
Cleorodes lichenaria|INV05784|Portugal|BOLD:AAC4210
Cleorodes lichenaria|INV01182|Portugal|BOLD:AAC4210
Chiasmia clathrata|INV03205|Portugal|BOLD:AAB0547
Chiasmia aestimaria|INV08980|Portugal|BOLD:AAB3595
Menophra japygiaria|INV01052|Portugal|BOLD:AAB6706
Menophra japygiaria|INV02484|Portugal|BOLD:AAB6706
Menophra japygiaria|INV02816|Portugal|BOLD:AAB6706
Menophra abruptaria|INV00917|Portugal|BOLD:ADL9374
Menophra abruptaria|INV02817|Portugal|BOLD:ADL9374
Menophra abruptaria|INV03005|Portugal|BOLD:ADL9374
Ourapteryx sambucaria|INV00752|Portugal|BOLD:AAB4472
Compsoptera opacaria|INV01834|Portugal|BOLD:AAY9371
Compsoptera opacaria|INV01879|Portugal|BOLD:AAY9371
Compsoptera opacaria|INV01822|Portugal|BOLD:AAY9371
Compsoptera jourdanaria|INV01897|Portugal|BOLD:AAR9924
Compsoptera jourdanaria|INV10226|Portugal|BOLD:AAR9924
Tephronia oranaria|INV01029|Portugal|BOLD:AAI0761
Tephronia oranaria|INV00824|Portugal|BOLD:AAI0761
Ectropis crepuscularia|INV04418|Portugal|BOLD:AAA2076
Ectropis crepuscularia|INV04851|Portugal|BOLD:AAA2076
Dyscia distinctaria|INV04456|Portugal|BOLD:AEC9756
Dyscia distinctaria|INV02863|Portugal|BOLD:ACF1137
Dyscia distinctaria|INV02385|Portugal|BOLD:ACF1137
Dyscia penulataria|INV04473|Portugal|BOLD:ACE4635
Dyscia penulataria|INV01951|Portugal|BOLD:AAC2402
Dyscia penulataria|INV06958|Portugal|BOLD:AAC2402
Ennomos alniaria|INV09921|Portugal|BOLD:AAB2314
Ennomos alniaria|INV01814|Portugal|BOLD:AAB2314
Ennomos alniaria|INV02485|Portugal|BOLD:ADT7084
Ennomos alniaria|INV00941|Portugal|BOLD:ADT7084
Ennomos quercaria|INV05318|Portugal|BOLD:AAC5645
Ennomos quercaria|INV01028|Portugal|BOLD:AAC5645
Ennomos quercaria|INV04051|Portugal|BOLD:AAC5645
Ennomos quercaria|INV09737|Portugal|BOLD:AAC5645
Ennomos fuscantaria|INV01890|Portugal|BOLD:ADS3575
Ennomos fuscantaria|INV00878|Portugal|BOLD:ADS3575
Ennomos fuscantaria|INV03080|Portugal|BOLD:ADS3575
Dicranura ulmi|INV04212|Portugal|BOLD:ACX8252
Perigune narbonea|INV04459|Portugal|BOLD:AAI4735
Perigune narbonea|INV02854|Portugal|BOLD:AAI4735
Perigune narbonea|INV05404|Portugal|BOLD:AAI4735
Biston strataria|INV04646|Portugal|BOLD:AAB4693
Biston betularia|INV00653|Portugal|BOLD:ABY9367
Biston betularia|INV03963|Portugal|BOLD:ABY9367
Stegania trimaculata|INV04460|Portugal|BOLD:AAC2483
Stegania trimaculata|INV00938|Portugal|BOLD:AAC2483
Stegania trimaculata|INV01342|Portugal|BOLD:AAC2483
Stegania trimaculata|INV03135|Portugal|BOLD:AAC2483
Lomaspilis marginata|INV01314|Portugal|BOLD:AAB5300
Malacosoma castrensis|INV01032|Portugal|BOLD:AAK7339
Malacosoma neustria|INV05304|Portugal|BOLD:AAD1119
Malacosoma neustria|INV01053|Portugal|BOLD:AAD1119
Malacosoma neustria|INV08732|Portugal|BOLD:AAD1119
Hemithea aestivaria|INV08977|Portugal|BOLD:AAA4524
Hemithea aestivaria|INV08999|Portugal|BOLD:AAA4524
Hemithea aestivaria|INV09062|Portugal|BOLD:AAA4522
Thalera fimbrialis|INV00936|Portugal|BOLD:AAC6840
Phaiogramma etruscaria|INV03103|Portugal|BOLD:ACW6537
Phaiogramma etruscaria|INV05278|Portugal|BOLD:ABY4065
Phaiogramma etruscaria|INV06502|Portugal|BOLD:ABY4065
Phaiogramma faustinata|INV10004|Portugal|BOLD:AAB4914
Phaiogramma faustinata|INV06910|Portugal|BOLD:AAB4914
Microloxia herbaria|INV06912|Portugal|BOLD:AEC3710
Microloxia herbaria|INV07485|Portugal|BOLD:AEC3710
Microloxia herbaria|INV03334|Portugal|BOLD:AEC2690
Acanthovalva inconspicuaria|INV07598|Portugal|BOLD:AAB5956
Crocallis dardoinaria|INV03947|Portugal|BOLD:AAF1568
Crocallis dardoinaria|INV01873|Portugal|BOLD:AAF1568
Nychiodes andalusiaria|INV05272|Portugal|BOLD:ABZ0919
Nychiodes andalusiaria|INV05305|Portugal|BOLD:ABZ0919
Nychiodes andalusiaria|INV05341|Portugal|BOLD:ABZ0919
Campaea margaritaria|INV03188|Portugal|BOLD:AAB5336
Campaea margaritaria|INV01865|Portugal|BOLD:AAB5336

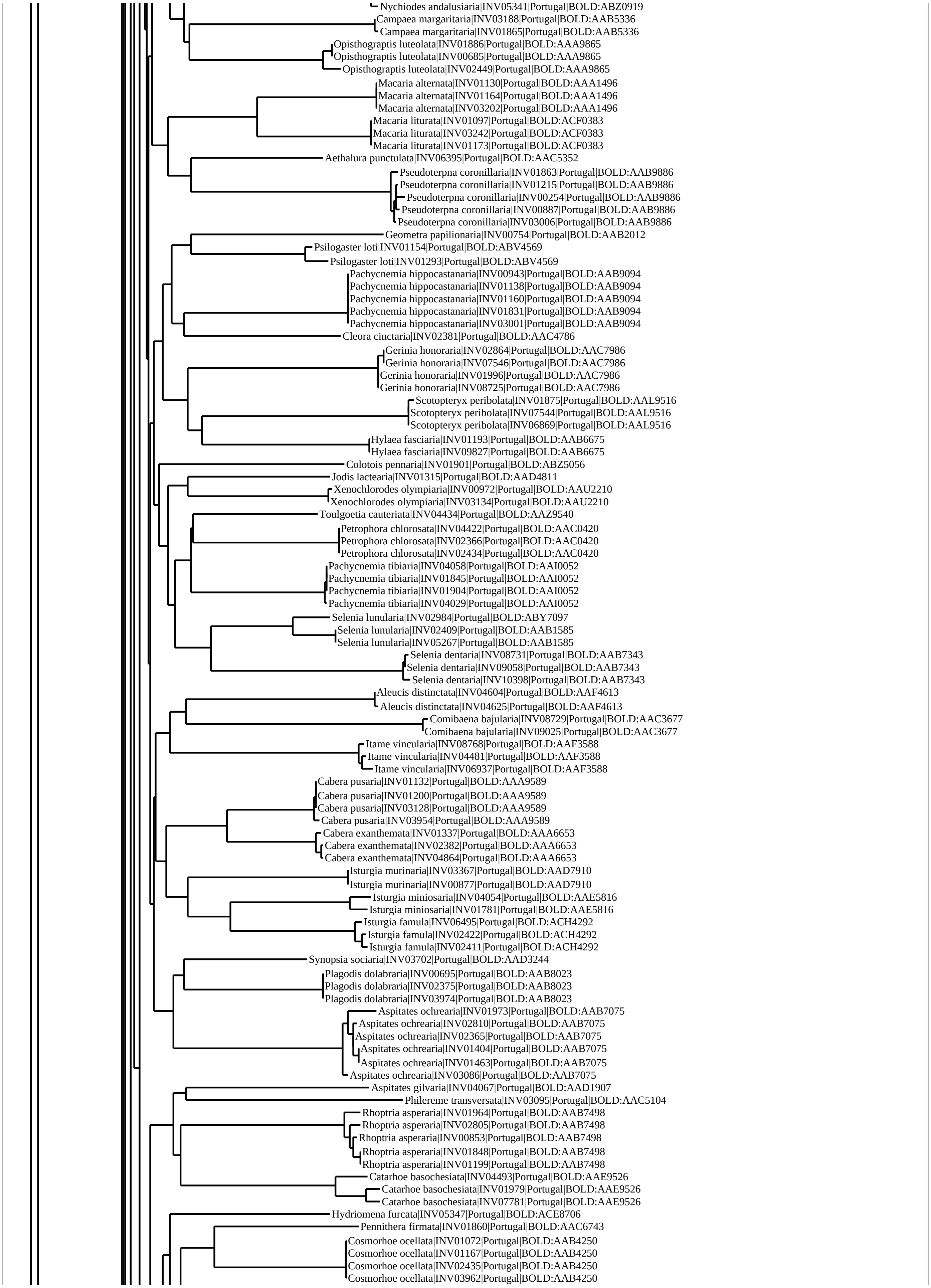
Nychiodes andalusiaria|INV05341|Portugal|BOLD:ABZ0919
Campaea margaritaria|INV03188|Portugal|BOLD:AAB5336
Campaea margaritaria|INV01865|Portugal|BOLD:AAB5336
Opisthograptis luteolata|INV01886|Portugal|BOLD:AAA9865
Opisthograptis luteolata|INV00685|Portugal|BOLD:AAA9865
Opisthograptis luteolata|INV02449|Portugal|BOLD:AAA9865
Macaria alternata|INV01130|Portugal|BOLD:AAA1496
Macaria alternata|INV01164|Portugal|BOLD:AAA1496
Macaria alternata|INV03202|Portugal|BOLD:AAA1496
Macaria liturata|INV01097|Portugal|BOLD:ACF0383
Macaria liturata|INV03242|Portugal|BOLD:ACF0383
Macaria liturata|INV01173|Portugal|BOLD:ACF0383
Aethalura punctulata|INV06395|Portugal|BOLD:AAC5352
Pseudoterpna coronillaria|INV01863|Portugal|BOLD:AAB9886
Pseudoterpna coronillaria|INV01215|Portugal|BOLD:AAB9886
Pseudoterpna coronillaria|INV00254|Portugal|BOLD:AAB9886
Pseudoterpna coronillaria|INV00887|Portugal|BOLD:AAB9886
Pseudoterpna coronillaria|INV03006|Portugal|BOLD:AAB9886
Geometra papilionaria|INV00754|Portugal|BOLD:AAB2012
Psilogaster loti|INV01154|Portugal|BOLD:ABV4569
Psilogaster loti|INV01293|Portugal|BOLD:ABV4569
Pachycnemia hippocastanaria|INV00943|Portugal|BOLD:AAB9094
Pachycnemia hippocastanaria|INV01138|Portugal|BOLD:AAB9094
Pachycnemia hippocastanaria|INV01160|Portugal|BOLD:AAB9094
Pachycnemia hippocastanaria|INV01831|Portugal|BOLD:AAB9094
Pachycnemia hippocastanaria|INV03001|Portugal|BOLD:AAB9094
Cleora cinctaria|INV02381|Portugal|BOLD:AAC4786
Gerinia honoraria|INV02864|Portugal|BOLD:AAC7986
Gerinia honoraria|INV07546|Portugal|BOLD:AAC7986
Gerinia honoraria|INV01996|Portugal|BOLD:AAC7986
Gerinia honoraria|INV08725|Portugal|BOLD:AAC7986
Scotopteryx peribolata|INV01875|Portugal|BOLD:AAL9516
Scotopteryx peribolata|INV07544|Portugal|BOLD:AAL9516
Scotopteryx peribolata|INV06869|Portugal|BOLD:AAL9516
Hylaea fasciaria|INV01193|Portugal|BOLD:AAB6675
Hylaea fasciaria|INV09827|Portugal|BOLD:AAB6675
Colotois pennaria|INV01901|Portugal|BOLD:ABZ5056
Jodis lactearia|INV01315|Portugal|BOLD:AAD4811
Xenochlorodes olympiaria|INV00972|Portugal|BOLD:AAU2210
Xenochlorodes olympiaria|INV03134|Portugal|BOLD:AAU2210
Toulgoetia cauteriata|INV04434|Portugal|BOLD:AAZ9540
Petrophora chlorosata|INV04422|Portugal|BOLD:AAC0420
Petrophora chlorosata|INV02366|Portugal|BOLD:AAC0420
Petrophora chlorosata|INV02434|Portugal|BOLD:AAC0420
Pachycnemia tibiaria|INV04058|Portugal|BOLD:AAI0052
Pachycnemia tibiaria|INV01845|Portugal|BOLD:AAI0052
Pachycnemia tibiaria|INV01904|Portugal|BOLD:AAI0052
Pachycnemia tibiaria|INV04029|Portugal|BOLD:AAI0052
Selenia lunularia|INV02984|Portugal|BOLD:ABY7097
Selenia lunularia|INV02409|Portugal|BOLD:AAB1585
Selenia lunularia|INV05267|Portugal|BOLD:AAB1585
Selenia dentaria|INV08731|Portugal|BOLD:AAB7343
Selenia dentaria|INV09058|Portugal|BOLD:AAB7343
Selenia dentaria|INV10398|Portugal|BOLD:AAB7343
Aleucis distinctata|INV04604|Portugal|BOLD:AAF4613
Aleucis distinctata|INV04625|Portugal|BOLD:AAF4613
Comibaena bajularia|INV08729|Portugal|BOLD:AAC3677
Comibaena bajularia|INV09025|Portugal|BOLD:AAC3677
Itame vincularia|INV08768|Portugal|BOLD:AAF3588
Itame vincularia|INV04481|Portugal|BOLD:AAF3588
Itame vincularia|INV06937|Portugal|BOLD:AAF3588
Cabera pusaria|INV01132|Portugal|BOLD:AAA9589
Cabera pusaria|INV01200|Portugal|BOLD:AAA9589
Cabera pusaria|INV03128|Portugal|BOLD:AAA9589
Cabera pusaria|INV03954|Portugal|BOLD:AAA9589
Cabera exanthemata|INV01337|Portugal|BOLD:AAA6653
Cabera exanthemata|INV02382|Portugal|BOLD:AAA6653
Cabera exanthemata|INV04864|Portugal|BOLD:AAA6653
Isturgia murinaria|INV03367|Portugal|BOLD:AAD7910
Isturgia murinaria|INV00877|Portugal|BOLD:AAD7910
Isturgia miniosaria|INV04054|Portugal|BOLD:AAE5816
Isturgia miniosaria|INV01781|Portugal|BOLD:AAE5816
Isturgia famula|INV06495|Portugal|BOLD:ACH4292
Isturgia famula|INV02422|Portugal|BOLD:ACH4292
Isturgia famula|INV02411|Portugal|BOLD:ACH4292
Synopsia sociaria|INV03702|Portugal|BOLD:AAD3244
Plagodis dolabraria|INV00695|Portugal|BOLD:AAB8023
Plagodis dolabraria|INV02375|Portugal|BOLD:AAB8023
Plagodis dolabraria|INV03974|Portugal|BOLD:AAB8023
Aspitates ochrearia|INV01973|Portugal|BOLD:AAB7075
Aspitates ochrearia|INV02810|Portugal|BOLD:AAB7075
Aspitates ochrearia|INV02365|Portugal|BOLD:AAB7075
Aspitates ochrearia|INV01404|Portugal|BOLD:AAB7075
Aspitates ochrearia|INV01463|Portugal|BOLD:AAB7075
Aspitates ochrearia|INV03086|Portugal|BOLD:AAB7075
Aspitates gilvaria|INV04067|Portugal|BOLD:AAD1907
Philereme transversata|INV03095|Portugal|BOLD:AAC5104
Rhoptria asperaria|INV01964|Portugal|BOLD:AAB7498
Rhoptria asperaria|INV02805|Portugal|BOLD:AAB7498
Rhoptria asperaria|INV00853|Portugal|BOLD:AAB7498
Rhoptria asperaria|INV01848|Portugal|BOLD:AAB7498
Rhoptria asperaria|INV01199|Portugal|BOLD:AAB7498
Catarhoe basochesiata|INV04493|Portugal|BOLD:AAE9526
Catarhoe basochesiata|INV01979|Portugal|BOLD:AAE9526
Catarhoe basochesiata|INV07781|Portugal|BOLD:AAE9526
Hydriomena furcata|INV05347|Portugal|BOLD:ACE8706
Pennithera firmata|INV01860|Portugal|BOLD:AAC6743
Cosmorhoe ocellata|INV01072|Portugal|BOLD:AAB4250
Cosmorhoe ocellata|INV01167|Portugal|BOLD:AAB4250
Cosmorhoe ocellata|INV02435|Portugal|BOLD:AAB4250
Cosmorhoe ocellata|INV03962|Portugal|BOLD:AAB4250

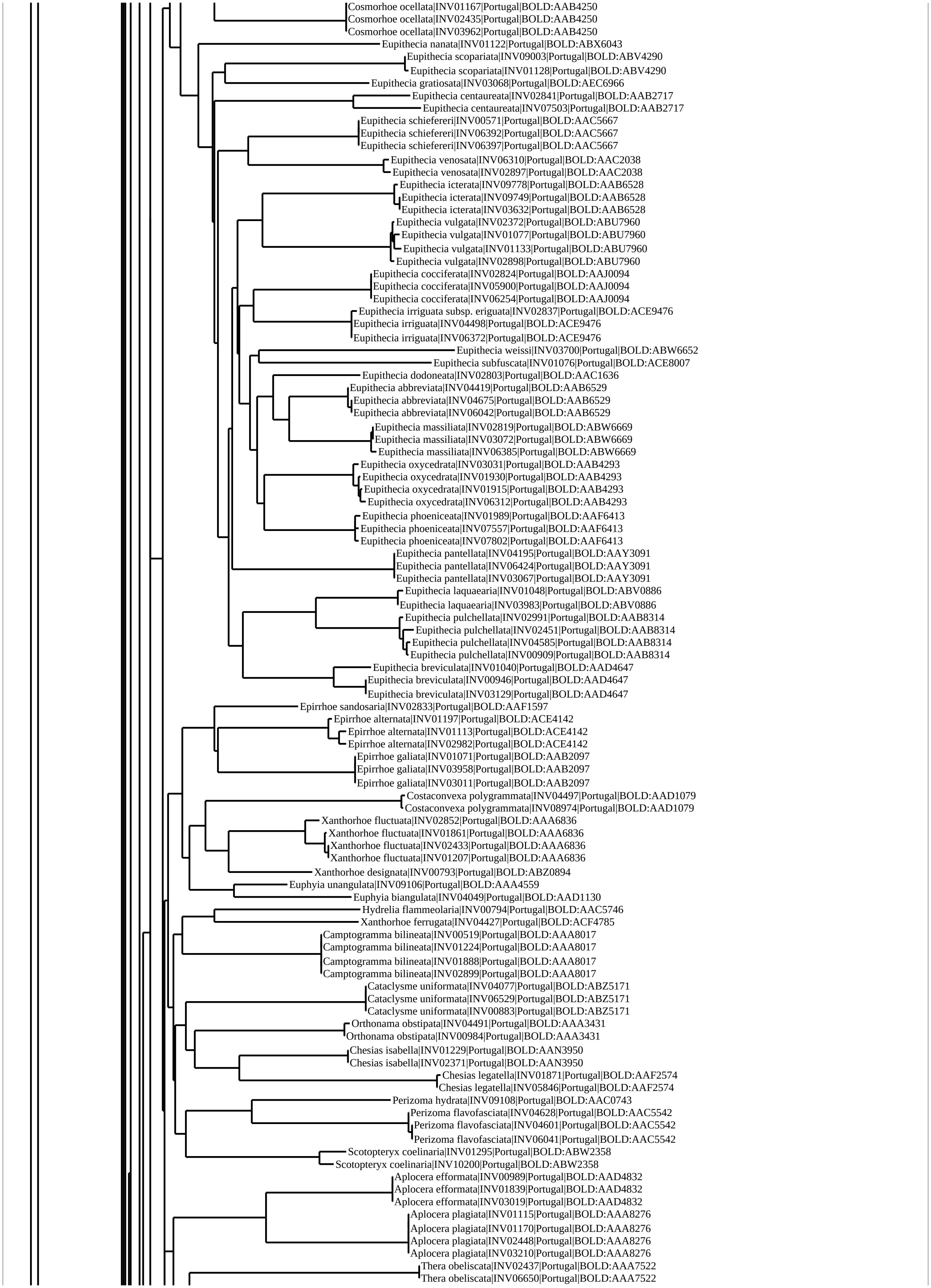
Cosmorhoe ocellata|INV01167|Portugal|BOLD:AAB4250
Cosmorhoe ocellata|INV02435|Portugal|BOLD:AAB4250
Cosmorhoe ocellata|INV03962|Portugal|BOLD:AAB4250
Eupithecia nanata|INV01122|Portugal|BOLD:ABX6043
Eupithecia scopariata|INV09003|Portugal|BOLD:ABV4290
Eupithecia scopariata|INV01128|Portugal|BOLD:ABV4290
Eupithecia gratiosata|INV03068|Portugal|BOLD:AEC6966
Eupithecia centaureata|INV02841|Portugal|BOLD:AAB2717
Eupithecia centaureata|INV07503|Portugal|BOLD:AAB2717
Eupithecia schiefereri|INV00571|Portugal|BOLD:AAC5667
Eupithecia schiefereri|INV06392|Portugal|BOLD:AAC5667
Eupithecia schiefereri|INV06397|Portugal|BOLD:AAC5667
Eupithecia venosata|INV06310|Portugal|BOLD:AAC2038
Eupithecia venosata|INV02897|Portugal|BOLD:AAC2038
Eupithecia icterata|INV09778|Portugal|BOLD:AAB6528
Eupithecia icterata|INV09749|Portugal|BOLD:AAB6528
Eupithecia icterata|INV03632|Portugal|BOLD:AAB6528
Eupithecia vulgata|INV02372|Portugal|BOLD:ABU7960
Eupithecia vulgata|INV01077|Portugal|BOLD:ABU7960
Eupithecia vulgata|INV01133|Portugal|BOLD:ABU7960
Eupithecia vulgata|INV02898|Portugal|BOLD:ABU7960
Eupithecia cocciferata|INV02824|Portugal|BOLD:AAJ0094
Eupithecia cocciferata|INV05900|Portugal|BOLD:AAJ0094
Eupithecia cocciferata|INV06254|Portugal|BOLD:AAJ0094
Eupithecia irriguata subsp. eriguata|INV02837|Portugal|BOLD:ACE9476
Eupithecia irriguata|INV04498|Portugal|BOLD:ACE9476
Eupithecia irriguata|INV06372|Portugal|BOLD:ACE9476
Eupithecia weissi|INV03700|Portugal|BOLD:ABW6652
Eupithecia subfuscata|INV01076|Portugal|BOLD:ACE8007
Eupithecia dodoneata|INV02803|Portugal|BOLD:AAC1636
Eupithecia abbreviata|INV04419|Portugal|BOLD:AAB6529
Eupithecia abbreviata|INV04675|Portugal|BOLD:AAB6529
Eupithecia abbreviata|INV06042|Portugal|BOLD:AAB6529
Eupithecia massiliata|INV02819|Portugal|BOLD:ABW6669
Eupithecia massiliata|INV03072|Portugal|BOLD:ABW6669
Eupithecia massiliata|INV06385|Portugal|BOLD:ABW6669
Eupithecia oxycedrata|INV03031|Portugal|BOLD:AAB4293
Eupithecia oxycedrata|INV01930|Portugal|BOLD:AAB4293
Eupithecia oxycedrata|INV01915|Portugal|BOLD:AAB4293
Eupithecia oxycedrata|INV06312|Portugal|BOLD:AAB4293
Eupithecia phoeniceata|INV01989|Portugal|BOLD:AAF6413
Eupithecia phoeniceata|INV07557|Portugal|BOLD:AAF6413
Eupithecia phoeniceata|INV07802|Portugal|BOLD:AAF6413
Eupithecia pantellata|INV04195|Portugal|BOLD:AAY3091
Eupithecia pantellata|INV06424|Portugal|BOLD:AAY3091
Eupithecia pantellata|INV03067|Portugal|BOLD:AAY3091
Eupithecia laquaearia|INV01048|Portugal|BOLD:ABV0886
Eupithecia laquaearia|INV03983|Portugal|BOLD:ABV0886
Eupithecia pulchellata|INV02991|Portugal|BOLD:AAB8314
Eupithecia pulchellata|INV02451|Portugal|BOLD:AAB8314
Eupithecia pulchellata|INV04585|Portugal|BOLD:AAB8314
Eupithecia pulchellata|INV00909|Portugal|BOLD:AAB8314
Eupithecia breviculata|INV01040|Portugal|BOLD:AAD4647
Eupithecia breviculata|INV00946|Portugal|BOLD:AAD4647
Eupithecia breviculata|INV03129|Portugal|BOLD:AAD4647
Epirrhoe sandosaria|INV02833|Portugal|BOLD:AAF1597
Epirrhoe alternata|INV01197|Portugal|BOLD:ACE4142
Epirrhoe alternata|INV01113|Portugal|BOLD:ACE4142
Epirrhoe alternata|INV02982|Portugal|BOLD:ACE4142
Epirrhoe galiata|INV01071|Portugal|BOLD:AAB2097
Epirrhoe galiata|INV03958|Portugal|BOLD:AAB2097
Epirrhoe galiata|INV03011|Portugal|BOLD:AAB2097
Costaconvexa polygrammata|INV04497|Portugal|BOLD:AAD1079
Costaconvexa polygrammata|INV08974|Portugal|BOLD:AAD1079
Xanthorhoe fluctuata|INV02852|Portugal|BOLD:AAA6836
Xanthorhoe fluctuata|INV01861|Portugal|BOLD:AAA6836
Xanthorhoe fluctuata|INV02433|Portugal|BOLD:AAA6836
Xanthorhoe fluctuata|INV01207|Portugal|BOLD:AAA6836
Xanthorhoe designata|INV00793|Portugal|BOLD:ABZ0894
Euphyia unangulata|INV09106|Portugal|BOLD:AAA4559
Euphyia biangulata|INV04049|Portugal|BOLD:AAD1130
Hydrelia flammeolaria|INV00794|Portugal|BOLD:AAC5746
Xanthorhoe ferrugata|INV04427|Portugal|BOLD:ACF4785
Camptogramma bilineata|INV00519|Portugal|BOLD:AAA8017
Camptogramma bilineata|INV01224|Portugal|BOLD:AAA8017
Camptogramma bilineata|INV01888|Portugal|BOLD:AAA8017
Camptogramma bilineata|INV02899|Portugal|BOLD:AAA8017
Cataclysme uniformata|INV04077|Portugal|BOLD:ABZ5171
Cataclysme uniformata|INV06529|Portugal|BOLD:ABZ5171
Cataclysme uniformata|INV00883|Portugal|BOLD:ABZ5171
Orthonama obstipata|INV04491|Portugal|BOLD:AAA3431
Orthonama obstipata|INV00984|Portugal|BOLD:AAA3431
Chesias isabella|INV01229|Portugal|BOLD:AAN3950
Chesias isabella|INV02371|Portugal|BOLD:AAN3950
Chesias legatella|INV01871|Portugal|BOLD:AAF2574
Chesias legatella|INV05846|Portugal|BOLD:AAF2574
Perizoma hydrata|INV09108|Portugal|BOLD:AAC0743
Perizoma flavofasciata|INV04628|Portugal|BOLD:AAC5542
Perizoma flavofasciata|INV04601|Portugal|BOLD:AAC5542
Perizoma flavofasciata|INV06041|Portugal|BOLD:AAC5542
Scotopteryx coelinaria|INV01295|Portugal|BOLD:ABW2358
Scotopteryx coelinaria|INV10200|Portugal|BOLD:ABW2358
Aplocera efformata|INV00989|Portugal|BOLD:AAD4832
Aplocera efformata|INV01839|Portugal|BOLD:AAD4832
Aplocera efformata|INV03019|Portugal|BOLD:AAD4832
Aplocera plagiata|INV01115|Portugal|BOLD:AAA8276
Aplocera plagiata|INV01170|Portugal|BOLD:AAA8276
Aplocera plagiata|INV02448|Portugal|BOLD:AAA8276
Aplocera plagiata|INV03210|Portugal|BOLD:AAA8276
Thera obeliscata|INV02437|Portugal|BOLD:AAA7522
Thera obeliscata|INV06650|Portugal|BOLD:AAA7522

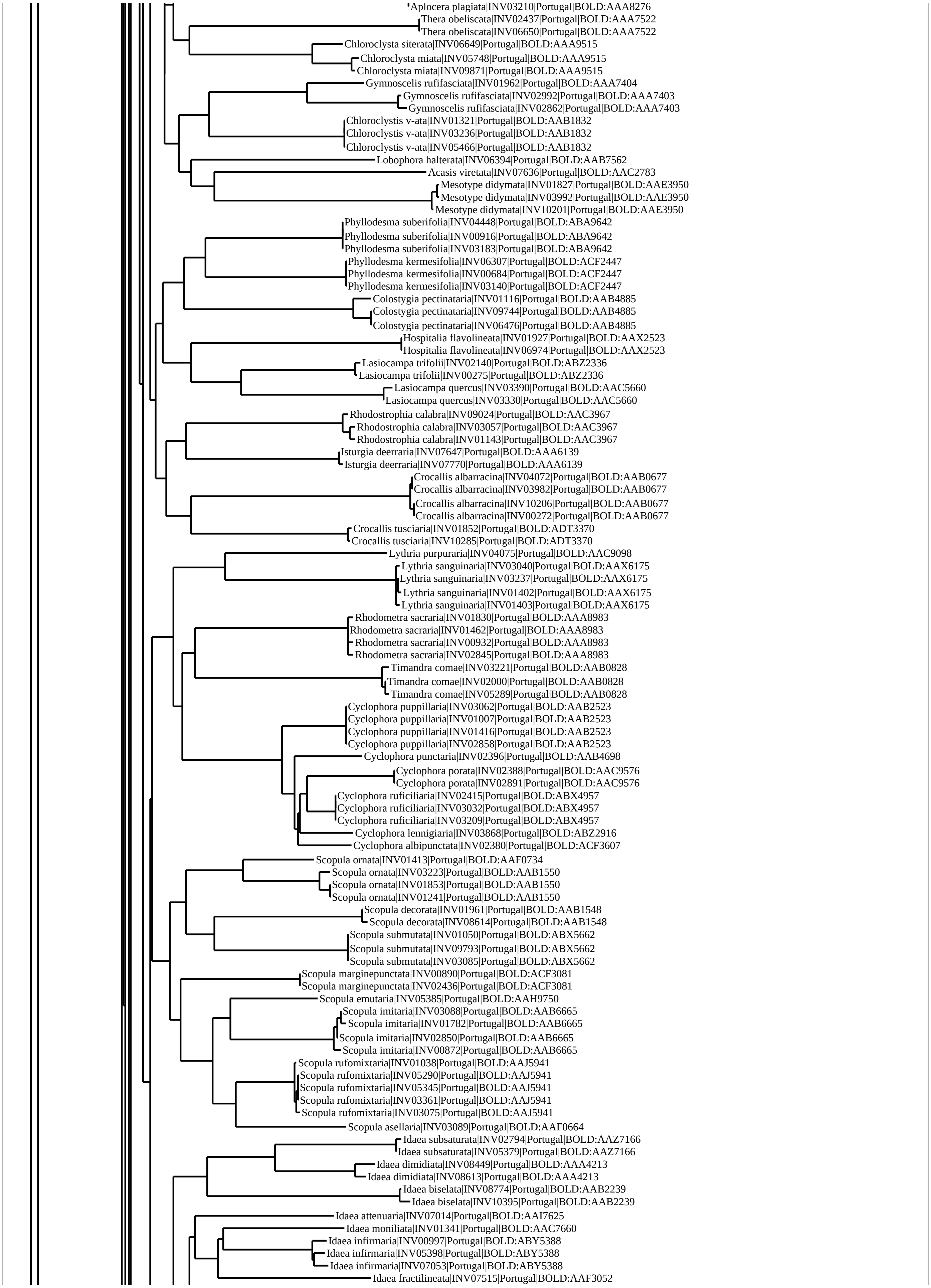

Aplocera plagiata|INV03210|Portugal|BOLD:AAA8276
Thera obeliscata|INV02437|Portugal|BOLD:AAA7522
Thera obeliscata|INV06650|Portugal|BOLD:AAA7522
Chloroclysta siterata|INV06649|Portugal|BOLD:AAA9515
Chloroclysta miata|INV05748|Portugal|BOLD:AAA9515
Chloroclysta miata|INV09871|Portugal|BOLD:AAA9515
Gymnoscelis rufifasciata|INV01962|Portugal|BOLD:AAA7404
Gymnoscelis rufifasciata|INV02992|Portugal|BOLD:AAA7403
Gymnoscelis rufifasciata|INV02862|Portugal|BOLD:AAA7403
Chloroclystis v-ata|INV01321|Portugal|BOLD:AAB1832
Chloroclystis v-ata|INV03236|Portugal|BOLD:AAB1832
Chloroclystis v-ata|INV05466|Portugal|BOLD:AAB1832
Lobophora halterata|INV06394|Portugal|BOLD:AAB7562
Acasis viretata|INV07636|Portugal|BOLD:AAC2783
Mesotype didymata|INV01827|Portugal|BOLD:AAE3950
Mesotype didymata|INV03992|Portugal|BOLD:AAE3950
Mesotype didymata|INV10201|Portugal|BOLD:AAE3950
Phyllodesma suberifolia|INV04448|Portugal|BOLD:ABA9642
Phyllodesma suberifolia|INV00916|Portugal|BOLD:ABA9642
Phyllodesma suberifolia|INV03183|Portugal|BOLD:ABA9642
Phyllodesma kermesifolia|INV06307|Portugal|BOLD:ACF2447
Phyllodesma kermesifolia|INV00684|Portugal|BOLD:ACF2447
Phyllodesma kermesifolia|INV03140|Portugal|BOLD:ACF2447
Colostygia pectinataria|INV01116|Portugal|BOLD:AAB4885
Colostygia pectinataria|INV09744|Portugal|BOLD:AAB4885
Colostygia pectinataria|INV06476|Portugal|BOLD:AAB4885
Hospitalia flavolineata|INV01927|Portugal|BOLD:AAX2523
Hospitalia flavolineata|INV06974|Portugal|BOLD:AAX2523
Lasiocampa trifolii|INV02140|Portugal|BOLD:ABZ2336
Lasiocampa trifolii|INV00275|Portugal|BOLD:ABZ2336
Lasiocampa quercus|INV03390|Portugal|BOLD:AAC5660
Lasiocampa quercus|INV03330|Portugal|BOLD:AAC5660
Rhodostrophia calabra|INV09024|Portugal|BOLD:AAC3967
Rhodostrophia calabra|INV03057|Portugal|BOLD:AAC3967
Rhodostrophia calabra|INV01143|Portugal|BOLD:AAC3967
Isturgia deerraria|INV07647|Portugal|BOLD:AAA6139
Isturgia deerraria|INV07770|Portugal|BOLD:AAA6139
Crocallis albarracina|INV04072|Portugal|BOLD:AAB0677
Crocallis albarracina|INV03982|Portugal|BOLD:AAB0677
Crocallis albarracina|INV10206|Portugal|BOLD:AAB0677
Crocallis albarracina|INV00272|Portugal|BOLD:AAB0677
Crocallis tusciaria|INV01852|Portugal|BOLD:ADT3370
Crocallis tusciaria|INV10285|Portugal|BOLD:ADT3370
Lythria purpuraria|INV04075|Portugal|BOLD:AAC9098
Lythria sanguinaria|INV03040|Portugal|BOLD:AAX6175
Lythria sanguinaria|INV03237|Portugal|BOLD:AAX6175
Lythria sanguinaria|INV01402|Portugal|BOLD:AAX6175
Lythria sanguinaria|INV01403|Portugal|BOLD:AAX6175
Rhodometra sacraria|INV01830|Portugal|BOLD:AAA8983
Rhodometra sacraria|INV01462|Portugal|BOLD:AAA8983
Rhodometra sacraria|INV00932|Portugal|BOLD:AAA8983
Rhodometra sacraria|INV02845|Portugal|BOLD:AAA8983
Timandra comae|INV03221|Portugal|BOLD:AAB0828
Timandra comae|INV02000|Portugal|BOLD:AAB0828
Timandra comae|INV05289|Portugal|BOLD:AAB0828
Cyclophora puppillaria|INV03062|Portugal|BOLD:AAB2523
Cyclophora puppillaria|INV01007|Portugal|BOLD:AAB2523
Cyclophora puppillaria|INV01416|Portugal|BOLD:AAB2523
Cyclophora puppillaria|INV02858|Portugal|BOLD:AAB2523
Cyclophora punctaria|INV02396|Portugal|BOLD:AAB4698
Cyclophora porata|INV02388|Portugal|BOLD:AAC9576
Cyclophora porata|INV02891|Portugal|BOLD:AAC9576
Cyclophora ruficiliaria|INV02415|Portugal|BOLD:ABX4957
Cyclophora ruficiliaria|INV03032|Portugal|BOLD:ABX4957
Cyclophora ruficiliaria|INV03209|Portugal|BOLD:ABX4957
Cyclophora lennigiaria|INV03868|Portugal|BOLD:ABZ2916
Cyclophora albipunctata|INV02380|Portugal|BOLD:ACF3607
Scopula ornata|INV01413|Portugal|BOLD:AAF0734
Scopula ornata|INV03223|Portugal|BOLD:AAB1550
Scopula ornata|INV01853|Portugal|BOLD:AAB1550
Scopula ornata|INV01241|Portugal|BOLD:AAB1550
Scopula decorata|INV01961|Portugal|BOLD:AAB1548
Scopula decorata|INV08614|Portugal|BOLD:AAB1548
Scopula submutata|INV01050|Portugal|BOLD:ABX5662
Scopula submutata|INV09793|Portugal|BOLD:ABX5662
Scopula submutata|INV03085|Portugal|BOLD:ABX5662
Scopula marginepunctata|INV00890|Portugal|BOLD:ACF3081
Scopula marginepunctata|INV02436|Portugal|BOLD:ACF3081
Scopula emutaria|INV05385|Portugal|BOLD:AAH9750
Scopula imitaria|INV03088|Portugal|BOLD:AAB6665
Scopula imitaria|INV01782|Portugal|BOLD:AAB6665
Scopula imitaria|INV02850|Portugal|BOLD:AAB6665
Scopula imitaria|INV00872|Portugal|BOLD:AAB6665
Scopula rufomixtaria|INV01038|Portugal|BOLD:AAJ5941
Scopula rufomixtaria|INV05290|Portugal|BOLD:AAJ5941
Scopula rufomixtaria|INV05345|Portugal|BOLD:AAJ5941
Scopula rufomixtaria|INV03361|Portugal|BOLD:AAJ5941
Scopula rufomixtaria|INV03075|Portugal|BOLD:AAJ5941
Scopula asellaria|INV03089|Portugal|BOLD:AAF0664
Idaea subsaturata|INV02794|Portugal|BOLD:AAZ7166
Idaea subsaturata|INV05379|Portugal|BOLD:AAZ7166
Idaea dimidiata|INV08449|Portugal|BOLD:AAA4213
Idaea dimidiata|INV08613|Portugal|BOLD:AAA4213
Idaea biselata|INV08774|Portugal|BOLD:AAB2239
Idaea biselata|INV10395|Portugal|BOLD:AAB2239
Idaea attenuaria|INV07014|Portugal|BOLD:AAI7625
Idaea moniliata|INV01341|Portugal|BOLD:AAC7660
Idaea infirmaria|INV00997|Portugal|BOLD:ABY5388
Idaea infirmaria|INV05398|Portugal|BOLD:ABY5388
Idaea infirmaria|INV07053|Portugal|BOLD:ABY5388
Idaea fractilineata|INV07515|Portugal|BOLD:AAF3052

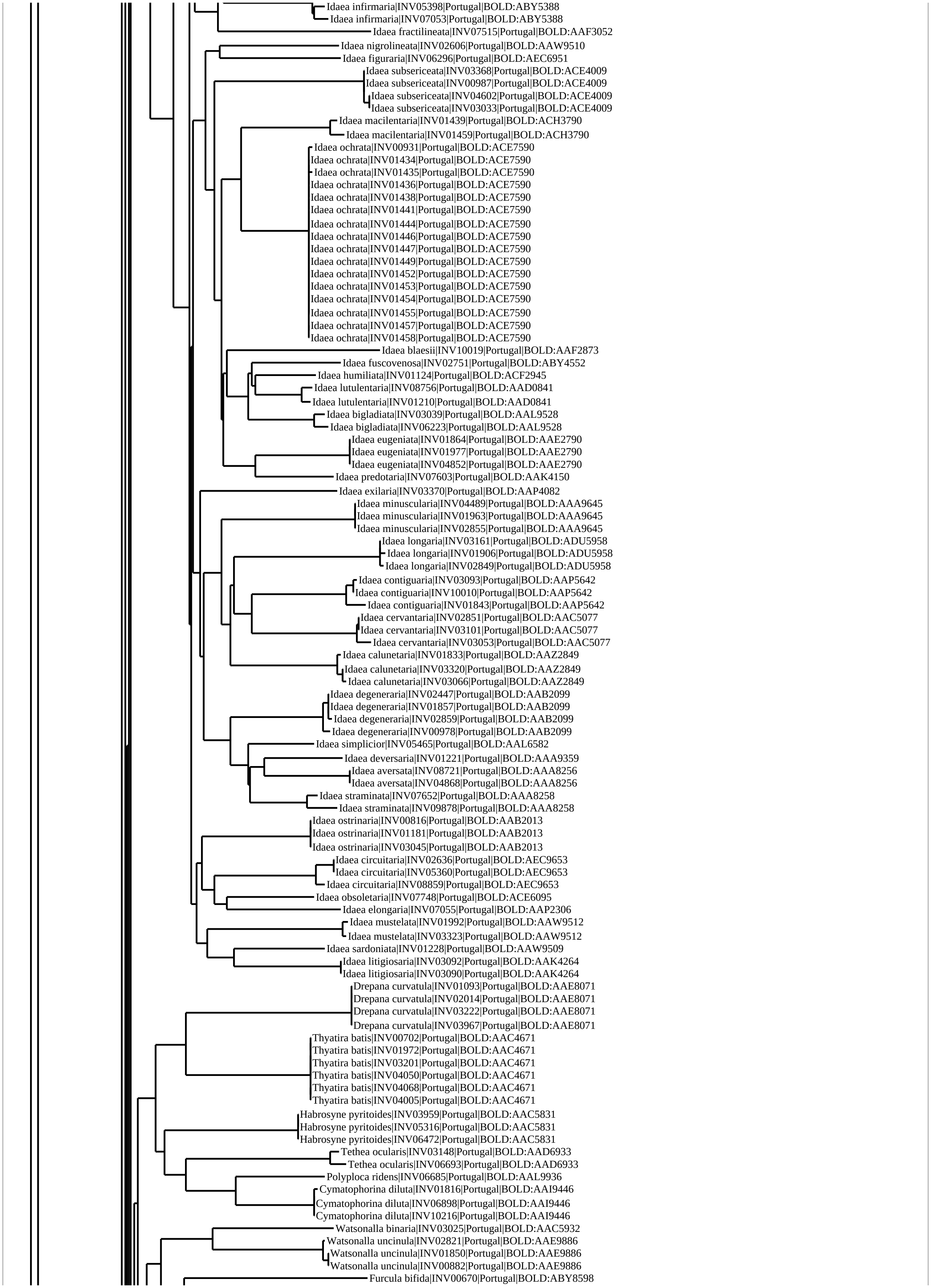
Idaea infirmaria|INV05398|Portugal|BOLD:ABY5388
Idaea infirmaria|INV07053|Portugal|BOLD:ABY5388
Idaea fractilineata|INV07515|Portugal|BOLD:AAF3052
Idaea nigrolineata|INV02606|Portugal|BOLD:AAW9510
Idaea figuraria|INV06296|Portugal|BOLD:AEC6951
Idaea subsericeata|INV03368|Portugal|BOLD:ACE4009
Idaea subsericeata|INV00987|Portugal|BOLD:ACE4009
Idaea subsericeata|INV04602|Portugal|BOLD:ACE4009
Idaea subsericeata|INV03033|Portugal|BOLD:ACE4009
Idaea macilentaria|INV01439|Portugal|BOLD:ACH3790
Idaea macilentaria|INV01459|Portugal|BOLD:ACH3790
Idaea ochrata|INV00931|Portugal|BOLD:ACE7590
Idaea ochrata|INV01434|Portugal|BOLD:ACE7590
Idaea ochrata|INV01435|Portugal|BOLD:ACE7590
Idaea ochrata|INV01436|Portugal|BOLD:ACE7590
Idaea ochrata|INV01438|Portugal|BOLD:ACE7590
Idaea ochrata|INV01441|Portugal|BOLD:ACE7590
Idaea ochrata|INV01444|Portugal|BOLD:ACE7590
Idaea ochrata|INV01446|Portugal|BOLD:ACE7590
Idaea ochrata|INV01447|Portugal|BOLD:ACE7590
Idaea ochrata|INV01449|Portugal|BOLD:ACE7590
Idaea ochrata|INV01452|Portugal|BOLD:ACE7590
Idaea ochrata|INV01453|Portugal|BOLD:ACE7590
Idaea ochrata|INV01454|Portugal|BOLD:ACE7590
Idaea ochrata|INV01455|Portugal|BOLD:ACE7590
Idaea ochrata|INV01457|Portugal|BOLD:ACE7590
Idaea ochrata|INV01458|Portugal|BOLD:ACE7590
Idaea blaesii|INV10019|Portugal|BOLD:AAF2873
Idaea fuscovenosa|INV02751|Portugal|BOLD:ABY4552
Idaea humiliata|INV01124|Portugal|BOLD:ACF2945
Idaea lutulentaria|INV08756|Portugal|BOLD:AAD0841
Idaea lutulentaria|INV01210|Portugal|BOLD:AAD0841
Idaea bigladiata|INV03039|Portugal|BOLD:AAL9528
Idaea bigladiata|INV06223|Portugal|BOLD:AAL9528
Idaea eugeniata|INV01864|Portugal|BOLD:AAE2790
Idaea eugeniata|INV01977|Portugal|BOLD:AAE2790
Idaea eugeniata|INV04852|Portugal|BOLD:AAE2790
Idaea predotaria|INV07603|Portugal|BOLD:AAK4150
Idaea exilaria|INV03370|Portugal|BOLD:AAP4082
Idaea minuscularia|INV04489|Portugal|BOLD:AAA9645
Idaea minuscularia|INV01963|Portugal|BOLD:AAA9645
Idaea minuscularia|INV02855|Portugal|BOLD:AAA9645
Idaea longaria|INV03161|Portugal|BOLD:ADU5958
Idaea longaria|INV01906|Portugal|BOLD:ADU5958
Idaea longaria|INV02849|Portugal|BOLD:ADU5958
Idaea contiguaria|INV03093|Portugal|BOLD:AAP5642
Idaea contiguaria|INV10010|Portugal|BOLD:AAP5642
Idaea contiguaria|INV01843|Portugal|BOLD:AAP5642
Idaea cervantaria|INV02851|Portugal|BOLD:AAC5077
Idaea cervantaria|INV03101|Portugal|BOLD:AAC5077
Idaea cervantaria|INV03053|Portugal|BOLD:AAC5077
Idaea calunetaria|INV01833|Portugal|BOLD:AAZ2849
Idaea calunetaria|INV03320|Portugal|BOLD:AAZ2849
Idaea calunetaria|INV03066|Portugal|BOLD:AAZ2849
Idaea degeneraria|INV02447|Portugal|BOLD:AAB2099
Idaea degeneraria|INV01857|Portugal|BOLD:AAB2099
Idaea degeneraria|INV02859|Portugal|BOLD:AAB2099
Idaea degeneraria|INV00978|Portugal|BOLD:AAB2099
Idaea simplicior|INV05465|Portugal|BOLD:AAL6582
Idaea deversaria|INV01221|Portugal|BOLD:AAA9359
Idaea aversata|INV08721|Portugal|BOLD:AAA8256
Idaea aversata|INV04868|Portugal|BOLD:AAA8256
Idaea straminata|INV07652|Portugal|BOLD:AAA8258
Idaea straminata|INV09878|Portugal|BOLD:AAA8258
Idaea ostrinaria|INV00816|Portugal|BOLD:AAB2013
Idaea ostrinaria|INV01181|Portugal|BOLD:AAB2013
Idaea ostrinaria|INV03045|Portugal|BOLD:AAB2013
Idaea circuitaria|INV02636|Portugal|BOLD:AEC9653
Idaea circuitaria|INV05360|Portugal|BOLD:AEC9653
Idaea circuitaria|INV08859|Portugal|BOLD:AEC9653
Idaea obsoletaria|INV07748|Portugal|BOLD:ACE6095
Idaea elongaria|INV07055|Portugal|BOLD:AAP2306
Idaea mustelata|INV01992|Portugal|BOLD:AAW9512
Idaea mustelata|INV03323|Portugal|BOLD:AAW9512
Idaea sardoniata|INV01228|Portugal|BOLD:AAW9509
Idaea litigiosaria|INV03092|Portugal|BOLD:AAK4264
Idaea litigiosaria|INV03090|Portugal|BOLD:AAK4264
Drepana curvatula|INV01093|Portugal|BOLD:AAE8071
Drepana curvatula|INV02014|Portugal|BOLD:AAE8071
Drepana curvatula|INV03222|Portugal|BOLD:AAE8071
Drepana curvatula|INV03967|Portugal|BOLD:AAE8071
Thyatira batis|INV00702|Portugal|BOLD:AAC4671
Thyatira batis|INV01972|Portugal|BOLD:AAC4671
Thyatira batis|INV03201|Portugal|BOLD:AAC4671
Thyatira batis|INV04050|Portugal|BOLD:AAC4671
Thyatira batis|INV04068|Portugal|BOLD:AAC4671
Thyatira batis|INV04005|Portugal|BOLD:AAC4671
Habrosyne pyritoides|INV03959|Portugal|BOLD:AAC5831
Habrosyne pyritoides|INV05316|Portugal|BOLD:AAC5831
Habrosyne pyritoides|INV06472|Portugal|BOLD:AAC5831
Tethea ocularis|INV03148|Portugal|BOLD:AAD6933
Tethea ocularis|INV06693|Portugal|BOLD:AAD6933
Polyploca ridens|INV06685|Portugal|BOLD:AAL9936
Cymatophorina diluta|INV01816|Portugal|BOLD:AAI9446
Cymatophorina diluta|INV06898|Portugal|BOLD:AAI9446
Cymatophorina diluta|INV10216|Portugal|BOLD:AAI9446
Watsonalla binaria|INV03025|Portugal|BOLD:AAC5932
Watsonalla uncinula|INV02821|Portugal|BOLD:AAE9886
Watsonalla uncinula|INV01850|Portugal|BOLD:AAE9886
Watsonalla uncinula|INV00882|Portugal|BOLD:AAE9886
Furcula bifida|INV00670|Portugal|BOLD:ABY8598

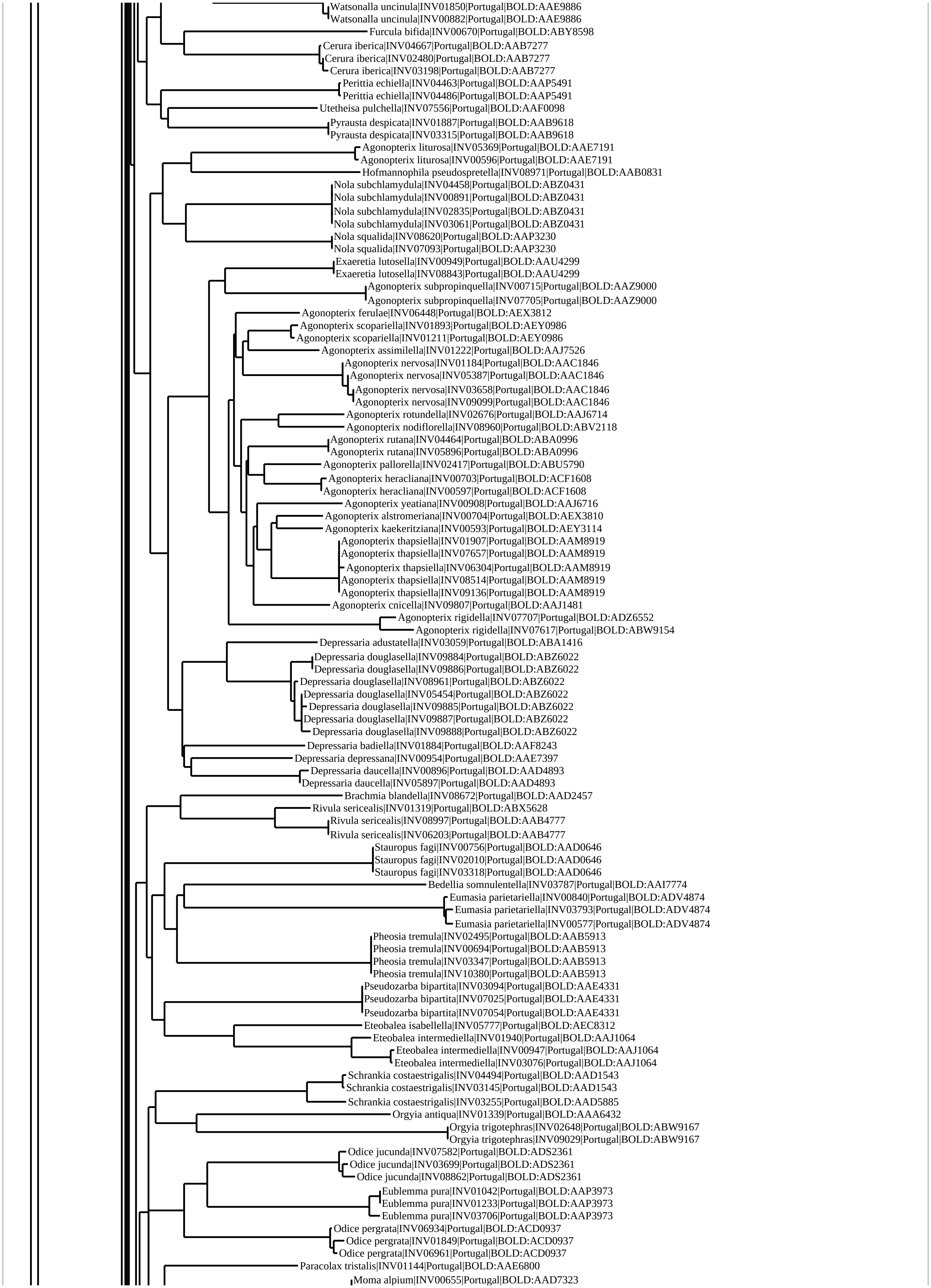
Watsonalla uncinula|INV01850|Portugal|BOLD:AAE9886
Watsonalla uncinula|INV00882|Portugal|BOLD:AAE9886
Furcula bifida|INV00670|Portugal|BOLD:ABY8598
Cerura iberica|INV04667|Portugal|BOLD:AAB7277
Cerura iberica|INV02480|Portugal|BOLD:AAB7277
Cerura iberica|INV03198|Portugal|BOLD:AAB7277
Perittia echiella|INV04463|Portugal|BOLD:AAP5491
Perittia echiella|INV04486|Portugal|BOLD:AAP5491
Utetheisa pulchella|INV07556|Portugal|BOLD:AAF0098
Pyrausta despicata|INV01887|Portugal|BOLD:AAB9618
Pyrausta despicata|INV03315|Portugal|BOLD:AAB9618
Agonopterix liturosa|INV05369|Portugal|BOLD:AAE7191
Agonopterix liturosa|INV00596|Portugal|BOLD:AAE7191
Hofmannophila pseudospretella|INV08971|Portugal|BOLD:AAB0831
Nola subchlamydula|INV04458|Portugal|BOLD:ABZ0431
Nola subchlamydula|INV00891|Portugal|BOLD:ABZ0431
Nola subchlamydula|INV02835|Portugal|BOLD:ABZ0431
Nola subchlamydula|INV03061|Portugal|BOLD:ABZ0431
Nola squalida|INV08620|Portugal|BOLD:AAP3230
Nola squalida|INV07093|Portugal|BOLD:AAP3230
Exaeretia lutosella|INV00949|Portugal|BOLD:AAU4299
Exaeretia lutosella|INV08843|Portugal|BOLD:AAU4299
Agonopterix subpropinquella|INV00715|Portugal|BOLD:AAZ9000
Agonopterix subpropinquella|INV07705|Portugal|BOLD:AAZ9000
Agonopterix ferulae|INV06448|Portugal|BOLD:AEX3812
Agonopterix scopariella|INV01893|Portugal|BOLD:AEY0986
Agonopterix scopariella|INV01211|Portugal|BOLD:AEY0986
Agonopterix assimilella|INV01222|Portugal|BOLD:AAJ7526
Agonopterix nervosa|INV01184|Portugal|BOLD:AAC1846
Agonopterix nervosa|INV05387|Portugal|BOLD:AAC1846
Agonopterix nervosa|INV03658|Portugal|BOLD:AAC1846
Agonopterix nervosa|INV09099|Portugal|BOLD:AAC1846
Agonopterix rotundella|INV02676|Portugal|BOLD:AAJ6714
Agonopterix nodiflorella|INV08960|Portugal|BOLD:ABV2118
Agonopterix rutana|INV04464|Portugal|BOLD:ABA0996
Agonopterix rutana|INV05896|Portugal|BOLD:ABA0996
Agonopterix pallorella|INV02417|Portugal|BOLD:ABU5790
Agonopterix heracliana|INV00703|Portugal|BOLD:ACF1608
Agonopterix heracliana|INV00597|Portugal|BOLD:ACF1608
Agonopterix yeatiana|INV00908|Portugal|BOLD:AAJ6716
Agonopterix alstromeriana|INV00704|Portugal|BOLD:AEX3810
Agonopterix kaekeritziana|INV00593|Portugal|BOLD:AEY3114
Agonopterix thapsiella|INV01907|Portugal|BOLD:AAM8919
Agonopterix thapsiella|INV07657|Portugal|BOLD:AAM8919
Agonopterix thapsiella|INV06304|Portugal|BOLD:AAM8919
Agonopterix thapsiella|INV08514|Portugal|BOLD:AAM8919
Agonopterix thapsiella|INV09136|Portugal|BOLD:AAM8919
Agonopterix cnicella|INV09807|Portugal|BOLD:AAJ1481
Agonopterix rigidella|INV07707|Portugal|BOLD:ADZ6552
Agonopterix rigidella|INV07617|Portugal|BOLD:ABW9154
Depressaria adustatella|INV03059|Portugal|BOLD:ABA1416
Depressaria douglasella|INV09884|Portugal|BOLD:ABZ6022
Depressaria douglasella|INV09886|Portugal|BOLD:ABZ6022
Depressaria douglasella|INV08961|Portugal|BOLD:ABZ6022
Depressaria douglasella|INV05454|Portugal|BOLD:ABZ6022
Depressaria douglasella|INV09885|Portugal|BOLD:ABZ6022
Depressaria douglasella|INV09887|Portugal|BOLD:ABZ6022
Depressaria douglasella|INV09888|Portugal|BOLD:ABZ6022
Depressaria badiella|INV01884|Portugal|BOLD:AAF8243
Depressaria depressana|INV00954|Portugal|BOLD:AAE7397
Depressaria daucella|INV00896|Portugal|BOLD:AAD4893
Depressaria daucella|INV05897|Portugal|BOLD:AAD4893
Brachmia blandella|INV08672|Portugal|BOLD:AAD2457
Rivula sericealis|INV01319|Portugal|BOLD:ABX5628
Rivula sericealis|INV08997|Portugal|BOLD:AAB4777
Rivula sericealis|INV06203|Portugal|BOLD:AAB4777
Stauropus fagi|INV00756|Portugal|BOLD:AAD0646
Stauropus fagi|INV02010|Portugal|BOLD:AAD0646
Stauropus fagi|INV03318|Portugal|BOLD:AAD0646
Bedellia somnulentella|INV03787|Portugal|BOLD:AAI7774
Eumasia parietariella|INV00840|Portugal|BOLD:ADV4874
Eumasia parietariella|INV03793|Portugal|BOLD:ADV4874
Eumasia parietariella|INV00577|Portugal|BOLD:ADV4874
Pheosia tremula|INV02495|Portugal|BOLD:AAB5913
Pheosia tremula|INV00694|Portugal|BOLD:AAB5913
Pheosia tremula|INV03347|Portugal|BOLD:AAB5913
Pheosia tremula|INV10380|Portugal|BOLD:AAB5913
Pseudozarba bipartita|INV03094|Portugal|BOLD:AAE4331
Pseudozarba bipartita|INV07025|Portugal|BOLD:AAE4331
Pseudozarba bipartita|INV07054|Portugal|BOLD:AAE4331
Eteobalea isabellella|INV05777|Portugal|BOLD:AEC8312
Eteobalea intermediella|INV01940|Portugal|BOLD:AAJ1064
Eteobalea intermediella|INV00947|Portugal|BOLD:AAJ1064
Eteobalea intermediella|INV03076|Portugal|BOLD:AAJ1064
Schrankia costaestrigalis|INV04494|Portugal|BOLD:AAD1543
Schrankia costaestrigalis|INV03145|Portugal|BOLD:AAD1543
Schrankia costaestrigalis|INV03255|Portugal|BOLD:AAD5885
Orgyia antiqua|INV01339|Portugal|BOLD:AAA6432
Orgyia trigotephras|INV02648|Portugal|BOLD:ABW9167
Orgyia trigotephras|INV09029|Portugal|BOLD:ABW9167
Odice jucunda|INV07582|Portugal|BOLD:ADS2361
Odice jucunda|INV03699|Portugal|BOLD:ADS2361
Odice jucunda|INV08862|Portugal|BOLD:ADS2361
Eublemma pura|INV01042|Portugal|BOLD:AAP3973
Eublemma pura|INV01233|Portugal|BOLD:AAP3973
Eublemma pura|INV03706|Portugal|BOLD:AAP3973
Odice pergrata|INV06934|Portugal|BOLD:ACD0937
Odice pergrata|INV01849|Portugal|BOLD:ACD0937
Odice pergrata|INV06961|Portugal|BOLD:ACD0937
Paracolax tristalis|INV01144|Portugal|BOLD:AAE6800
Moma alpium|INV00655|Portugal|BOLD:AAD7323

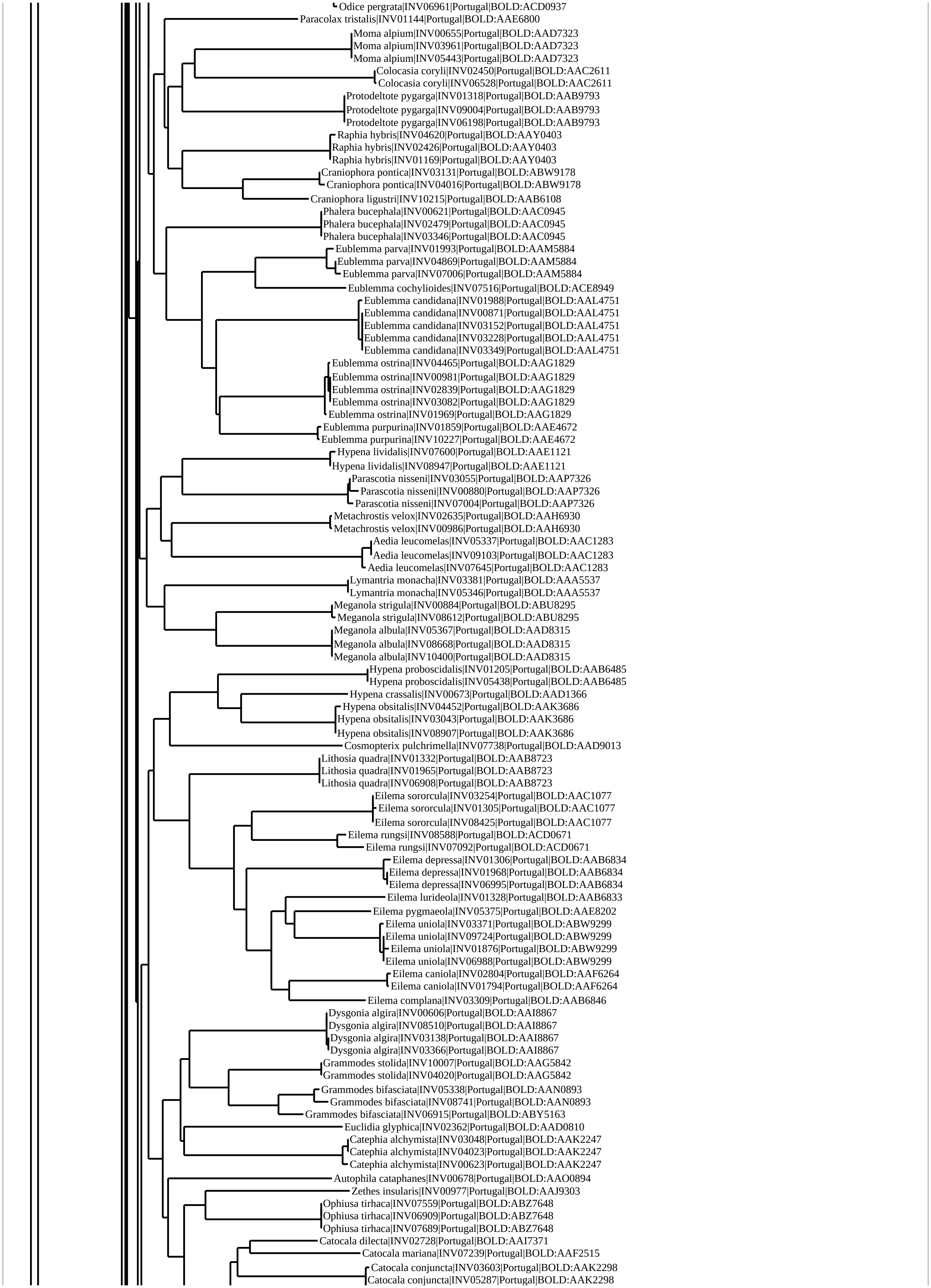
Odice pergrata|INV06961|Portugal|BOLD:ACD0937
Paracolax tristalis|INV01144|Portugal|BOLD:AAE6800
Moma alpium|INV00655|Portugal|BOLD:AAD7323
Moma alpium|INV03961|Portugal|BOLD:AAD7323
Moma alpium|INV05443|Portugal|BOLD:AAD7323
Colocasia coryli|INV02450|Portugal|BOLD:AAC2611
Colocasia coryli|INV06528|Portugal|BOLD:AAC2611
Protodeltote pygarga|INV01318|Portugal|BOLD:AAB9793
Protodeltote pygarga|INV09004|Portugal|BOLD:AAB9793
Protodeltote pygarga|INV06198|Portugal|BOLD:AAB9793
Raphia hybris|INV04620|Portugal|BOLD:AAY0403
Raphia hybris|INV02426|Portugal|BOLD:AAY0403
Raphia hybris|INV01169|Portugal|BOLD:AAY0403
Craniophora pontica|INV03131|Portugal|BOLD:ABW9178
Craniophora pontica|INV04016|Portugal|BOLD:ABW9178
Craniophora ligustri|INV10215|Portugal|BOLD:AAB6108
Phalera bucephala|INV00621|Portugal|BOLD:AAC0945
Phalera bucephala|INV02479|Portugal|BOLD:AAC0945
Phalera bucephala|INV03346|Portugal|BOLD:AAC0945
Eublemma parva|INV01993|Portugal|BOLD:AAM5884
Eublemma parva|INV04869|Portugal|BOLD:AAM5884
Eublemma parva|INV07006|Portugal|BOLD:AAM5884
Eublemma cochylioides|INV07516|Portugal|BOLD:ACE8949
Eublemma candidana|INV01988|Portugal|BOLD:AAL4751
Eublemma candidana|INV00871|Portugal|BOLD:AAL4751
Eublemma candidana|INV03152|Portugal|BOLD:AAL4751
Eublemma candidana|INV03228|Portugal|BOLD:AAL4751
Eublemma candidana|INV03349|Portugal|BOLD:AAL4751
Eublemma ostrina|INV04465|Portugal|BOLD:AAG1829
Eublemma ostrina|INV00981|Portugal|BOLD:AAG1829
Eublemma ostrina|INV02839|Portugal|BOLD:AAG1829
Eublemma ostrina|INV03082|Portugal|BOLD:AAG1829
Eublemma ostrina|INV01969|Portugal|BOLD:AAG1829
Eublemma purpurina|INV01859|Portugal|BOLD:AAE4672
Eublemma purpurina|INV10227|Portugal|BOLD:AAE4672
Hypena lividalis|INV07600|Portugal|BOLD:AAE1121
Hypena lividalis|INV08947|Portugal|BOLD:AAE1121
Parascotia nisseni|INV03055|Portugal|BOLD:AAP7326
Parascotia nisseni|INV00880|Portugal|BOLD:AAP7326
Parascotia nisseni|INV07004|Portugal|BOLD:AAP7326
Metachrostis velox|INV02635|Portugal|BOLD:AAH6930
Metachrostis velox|INV00986|Portugal|BOLD:AAH6930
Aedia leucomelas|INV05337|Portugal|BOLD:AAC1283
Aedia leucomelas|INV09103|Portugal|BOLD:AAC1283
Aedia leucomelas|INV07645|Portugal|BOLD:AAC1283
Lymantria monacha|INV03381|Portugal|BOLD:AAA5537
Lymantria monacha|INV05346|Portugal|BOLD:AAA5537
Meganola strigula|INV00884|Portugal|BOLD:ABU8295
Meganola strigula|INV08612|Portugal|BOLD:ABU8295
Meganola albula|INV05367|Portugal|BOLD:AAD8315
Meganola albula|INV08668|Portugal|BOLD:AAD8315
Meganola albula|INV10400|Portugal|BOLD:AAD8315
Hypena proboscidalis|INV01205|Portugal|BOLD:AAB6485
Hypena proboscidalis|INV05438|Portugal|BOLD:AAB6485
Hypena crassalis|INV00673|Portugal|BOLD:AAD1366
Hypena obsitalis|INV04452|Portugal|BOLD:AAK3686
Hypena obsitalis|INV03043|Portugal|BOLD:AAK3686
Hypena obsitalis|INV08907|Portugal|BOLD:AAK3686
Cosmopterix pulchrimella|INV07738|Portugal|BOLD:AAD9013
Lithosia quadra|INV01332|Portugal|BOLD:AAB8723
Lithosia quadra|INV01965|Portugal|BOLD:AAB8723
Lithosia quadra|INV06908|Portugal|BOLD:AAB8723
Eilema sororcula|INV03254|Portugal|BOLD:AAC1077
Eilema sororcula|INV01305|Portugal|BOLD:AAC1077
Eilema sororcula|INV08425|Portugal|BOLD:AAC1077
Eilema rungsi|INV08588|Portugal|BOLD:ACD0671
Eilema rungsi|INV07092|Portugal|BOLD:ACD0671
Eilema depressa|INV01306|Portugal|BOLD:AAB6834
Eilema depressa|INV01968|Portugal|BOLD:AAB6834
Eilema depressa|INV06995|Portugal|BOLD:AAB6834
Eilema lurideola|INV01328|Portugal|BOLD:AAB6833
Eilema pygmaeola|INV05375|Portugal|BOLD:AAE8202
Eilema uniola|INV03371|Portugal|BOLD:ABW9299
Eilema uniola|INV09724|Portugal|BOLD:ABW9299
Eilema uniola|INV01876|Portugal|BOLD:ABW9299
Eilema uniola|INV06988|Portugal|BOLD:ABW9299
Eilema caniola|INV02804|Portugal|BOLD:AAF6264
Eilema caniola|INV01794|Portugal|BOLD:AAF6264
Eilema complana|INV03309|Portugal|BOLD:AAB6846
Dysgonia algira|INV00606|Portugal|BOLD:AAI8867
Dysgonia algira|INV08510|Portugal|BOLD:AAI8867
Dysgonia algira|INV03138|Portugal|BOLD:AAI8867
Dysgonia algira|INV03366|Portugal|BOLD:AAI8867
Grammodes stolida|INV10007|Portugal|BOLD:AAG5842
Grammodes stolida|INV04020|Portugal|BOLD:AAG5842
Grammodes bifasciata|INV05338|Portugal|BOLD:AAN0893
Grammodes bifasciata|INV08741|Portugal|BOLD:AAN0893
Grammodes bifasciata|INV06915|Portugal|BOLD:ABY5163
Euclidia glyphica|INV02362|Portugal|BOLD:AAD0810
Catephia alchymista|INV03048|Portugal|BOLD:AAK2247
Catephia alchymista|INV04023|Portugal|BOLD:AAK2247
Catephia alchymista|INV00623|Portugal|BOLD:AAK2247
Autophila cataphanes|INV00678|Portugal|BOLD:AAO0894
Zethes insularis|INV00977|Portugal|BOLD:AAJ9303
Ophiusa tirhaca|INV07559|Portugal|BOLD:ABZ7648
Ophiusa tirhaca|INV06909|Portugal|BOLD:ABZ7648
Ophiusa tirhaca|INV07689|Portugal|BOLD:ABZ7648
Catocala dilecta|INV02728|Portugal|BOLD:AAI7371
Catocala mariana|INV07239|Portugal|BOLD:AAF2515
Catocala conjuncta|INV03603|Portugal|BOLD:AAK2298
Catocala conjuncta|INV05287|Portugal|BOLD:AAK2298

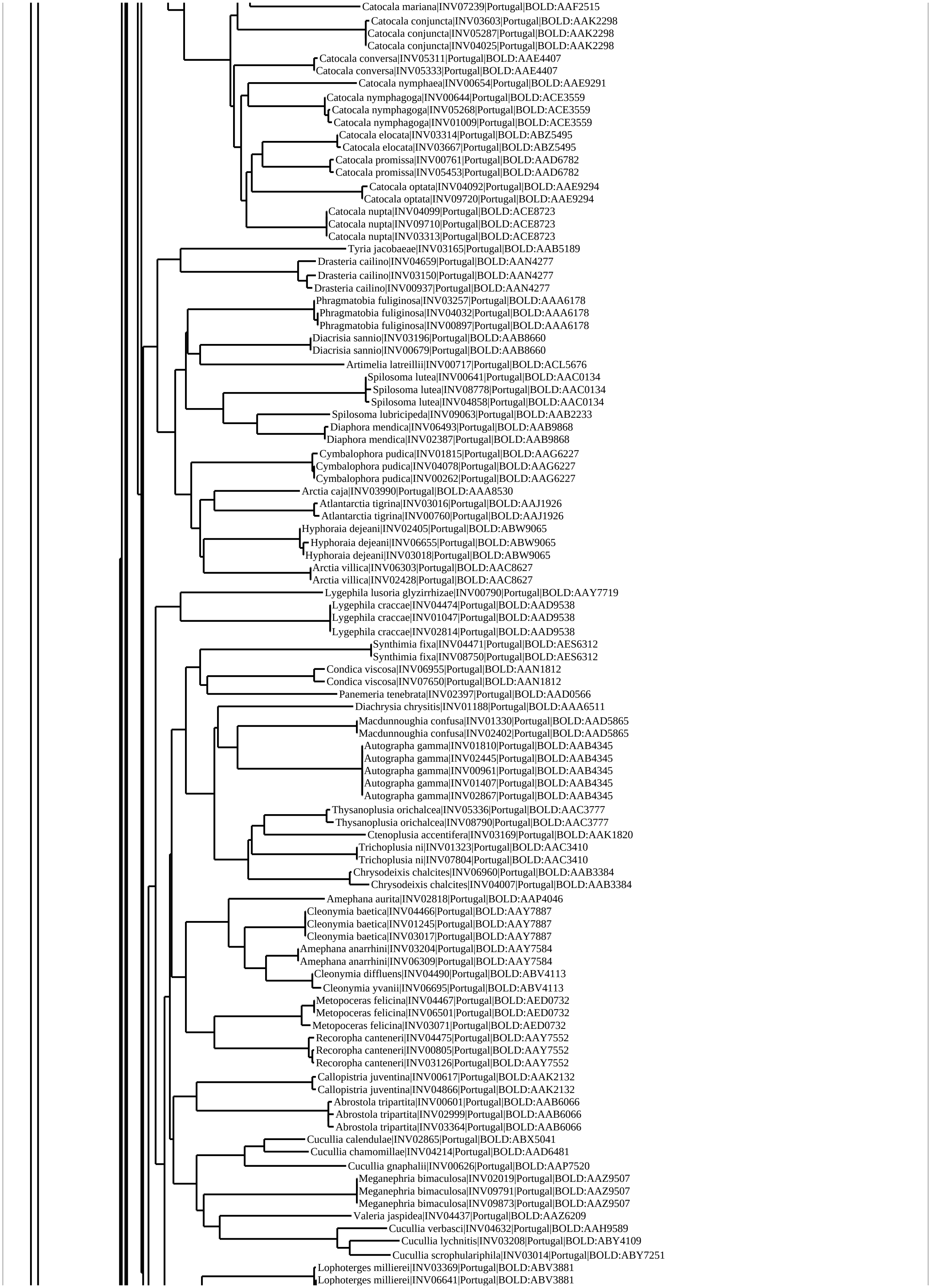
Catocala mariana|INV07239|Portugal|BOLD:AAF2515
Catocala conjuncta|INV03603|Portugal|BOLD:AAK2298
Catocala conjuncta|INV05287|Portugal|BOLD:AAK2298
Catocala conjuncta|INV04025|Portugal|BOLD:AAK2298
Catocala conversa|INV05311|Portugal|BOLD:AAE4407
Catocala conversa|INV05333|Portugal|BOLD:AAE4407
Catocala nymphaea|INV00654|Portugal|BOLD:AAE9291
Catocala nymphagoga|INV00644|Portugal|BOLD:ACE3559
Catocala nymphagoga|INV05268|Portugal|BOLD:ACE3559
Catocala nymphagoga|INV01009|Portugal|BOLD:ACE3559
Catocala elocata|INV03314|Portugal|BOLD:ABZ5495
Catocala elocata|INV03667|Portugal|BOLD:ABZ5495
Catocala promissa|INV00761|Portugal|BOLD:AAD6782
Catocala promissa|INV05453|Portugal|BOLD:AAD6782
Catocala optata|INV04092|Portugal|BOLD:AAE9294
Catocala optata|INV09720|Portugal|BOLD:AAE9294
Catocala nupta|INV04099|Portugal|BOLD:ACE8723
Catocala nupta|INV09710|Portugal|BOLD:ACE8723
Catocala nupta|INV03313|Portugal|BOLD:ACE8723
Tyria jacobaeae|INV03165|Portugal|BOLD:AAB5189
Drasteria cailino|INV04659|Portugal|BOLD:AAN4277
Drasteria cailino|INV03150|Portugal|BOLD:AAN4277
Drasteria cailino|INV00937|Portugal|BOLD:AAN4277
Phragmatobia fuliginosa|INV03257|Portugal|BOLD:AAA6178
Phragmatobia fuliginosa|INV04032|Portugal|BOLD:AAA6178
Phragmatobia fuliginosa|INV00897|Portugal|BOLD:AAA6178
Diacrisia sannio|INV03196|Portugal|BOLD:AAB8660
Diacrisia sannio|INV00679|Portugal|BOLD:AAB8660
Artimelia latreillii|INV00717|Portugal|BOLD:ACL5676
Spilosoma lutea|INV00641|Portugal|BOLD:AAC0134
Spilosoma lutea|INV08778|Portugal|BOLD:AAC0134
Spilosoma lutea|INV04858|Portugal|BOLD:AAC0134
Spilosoma lubricipeda|INV09063|Portugal|BOLD:AAB2233
Diaphora mendica|INV06493|Portugal|BOLD:AAB9868
Diaphora mendica|INV02387|Portugal|BOLD:AAB9868
Cymbalophora pudica|INV01815|Portugal|BOLD:AAG6227
Cymbalophora pudica|INV04078|Portugal|BOLD:AAG6227
Cymbalophora pudica|INV00262|Portugal|BOLD:AAG6227
Arctia caja|INV03990|Portugal|BOLD:AAA8530
Atlantarctia tigrina|INV03016|Portugal|BOLD:AAJ1926
Atlantarctia tigrina|INV00760|Portugal|BOLD:AAJ1926
Hyphoraia dejeani|INV02405|Portugal|BOLD:ABW9065
Hyphoraia dejeani|INV06655|Portugal|BOLD:ABW9065
Hyphoraia dejeani|INV03018|Portugal|BOLD:ABW9065
Arctia villica|INV06303|Portugal|BOLD:AAC8627
Arctia villica|INV02428|Portugal|BOLD:AAC8627
Lygephila lusoria glyzirrhizae|INV00790|Portugal|BOLD:AAY7719
Lygephila craccae|INV04474|Portugal|BOLD:AAD9538
Lygephila craccae|INV01047|Portugal|BOLD:AAD9538
Lygephila craccae|INV02814|Portugal|BOLD:AAD9538
Synthimia fixa|INV04471|Portugal|BOLD:AES6312
Synthimia fixa|INV08750|Portugal|BOLD:AES6312
Condica viscosa|INV06955|Portugal|BOLD:AAN1812
Condica viscosa|INV07650|Portugal|BOLD:AAN1812
Panemeria tenebrata|INV02397|Portugal|BOLD:AAD0566
Diachrysia chrysitis|INV01188|Portugal|BOLD:AAA6511
Macdunnoughia confusa|INV01330|Portugal|BOLD:AAD5865
Macdunnoughia confusa|INV02402|Portugal|BOLD:AAD5865
Autographa gamma|INV01810|Portugal|BOLD:AAB4345
Autographa gamma|INV02445|Portugal|BOLD:AAB4345
Autographa gamma|INV00961|Portugal|BOLD:AAB4345
Autographa gamma|INV01407|Portugal|BOLD:AAB4345
Autographa gamma|INV02867|Portugal|BOLD:AAB4345
Thysanoplusia orichalcea|INV05336|Portugal|BOLD:AAC3777
Thysanoplusia orichalcea|INV08790|Portugal|BOLD:AAC3777
Ctenoplusia accentifera|INV03169|Portugal|BOLD:AAK1820
Trichoplusia ni|INV01323|Portugal|BOLD:AAC3410
Trichoplusia ni|INV07804|Portugal|BOLD:AAC3410
Chrysodeixis chalcites|INV06960|Portugal|BOLD:AAB3384
Chrysodeixis chalcites|INV04007|Portugal|BOLD:AAB3384
Amephana aurita|INV02818|Portugal|BOLD:AAP4046
Cleonymia baetica|INV04466|Portugal|BOLD:AAY7887
Cleonymia baetica|INV01245|Portugal|BOLD:AAY7887
Cleonymia baetica|INV03017|Portugal|BOLD:AAY7887
Amephana anarrhini|INV03204|Portugal|BOLD:AAY7584
Amephana anarrhini|INV06309|Portugal|BOLD:AAY7584
Cleonymia diffluens|INV04490|Portugal|BOLD:ABV4113
Cleonymia yvanii|INV06695|Portugal|BOLD:ABV4113
Metopoceras felicina|INV04467|Portugal|BOLD:AED0732
Metopoceras felicina|INV06501|Portugal|BOLD:AED0732
Metopoceras felicina|INV03071|Portugal|BOLD:AED0732
Recoropha canteneri|INV04475|Portugal|BOLD:AAY7552
Recoropha canteneri|INV00805|Portugal|BOLD:AAY7552
Recoropha canteneri|INV03126|Portugal|BOLD:AAY7552
Callopistria juventina|INV00617|Portugal|BOLD:AAK2132
Callopistria juventina|INV04866|Portugal|BOLD:AAK2132
Abrostola tripartita|INV00601|Portugal|BOLD:AAB6066
Abrostola tripartita|INV02999|Portugal|BOLD:AAB6066
Abrostola tripartita|INV03364|Portugal|BOLD:AAB6066
Cucullia calendulae|INV02865|Portugal|BOLD:ABX5041
Cucullia chamomillae|INV04214|Portugal|BOLD:AAD6481
Cucullia gnaphalii|INV00626|Portugal|BOLD:AAP7520
Meganephria bimaculosa|INV02019|Portugal|BOLD:AAZ9507
Meganephria bimaculosa|INV09791|Portugal|BOLD:AAZ9507
Meganephria bimaculosa|INV09873|Portugal|BOLD:AAZ9507
Valeria jaspidea|INV04437|Portugal|BOLD:AAZ6209
Cucullia verbasci|INV04632|Portugal|BOLD:AAH9589
Cucullia lychnitis|INV03208|Portugal|BOLD:ABY4109
Cucullia scrophulariphila|INV03014|Portugal|BOLD:ABY7251
Lophoterges millierei|INV03369|Portugal|BOLD:ABV3881
Lophoterges millierei|INV06641|Portugal|BOLD:ABV3881

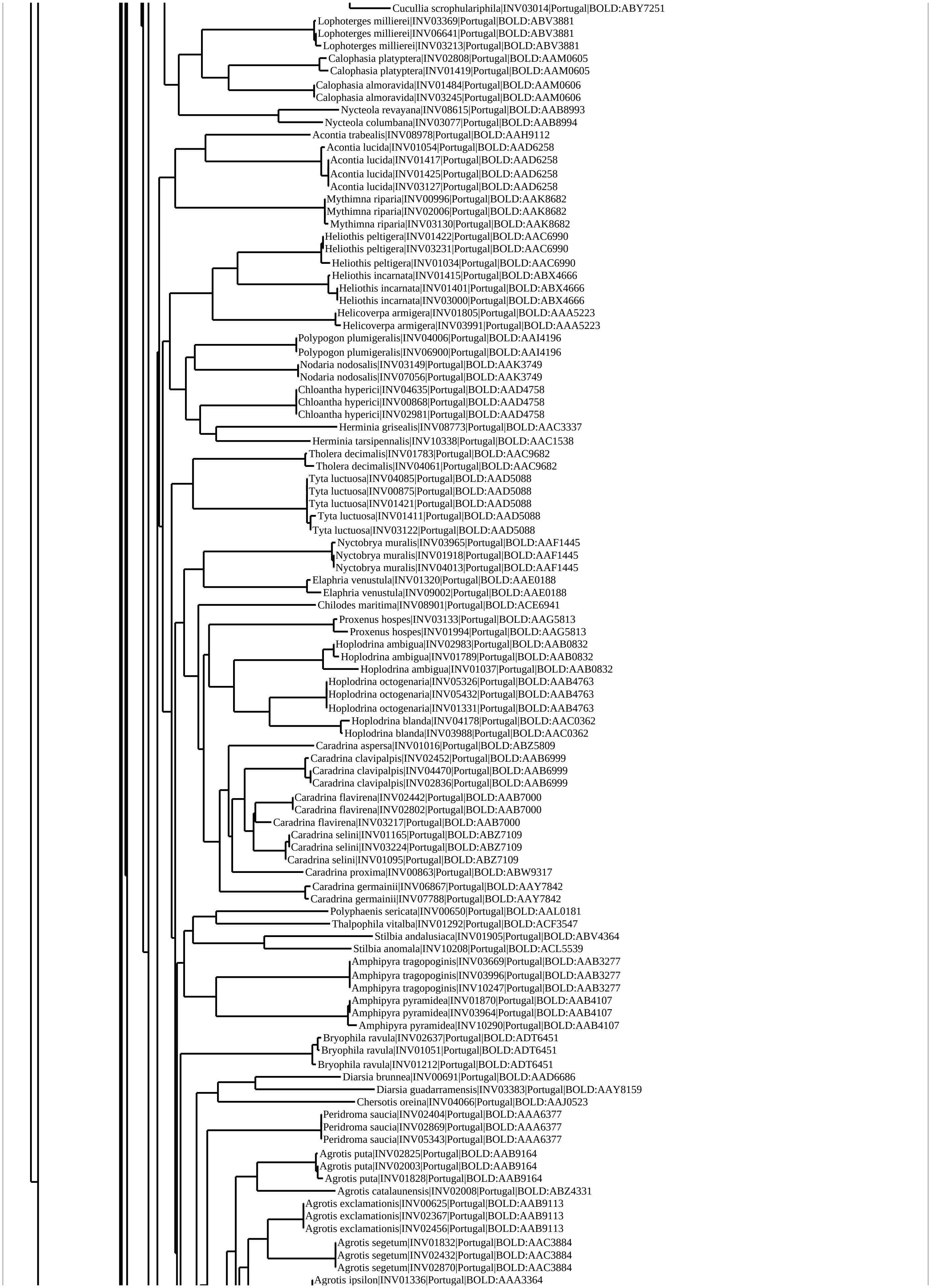
Cucullia scrophulariphila|INV03014|Portugal|BOLD:ABY7251
Lophoterges millierei|INV03369|Portugal|BOLD:ABV3881
Lophoterges millierei|INV06641|Portugal|BOLD:ABV3881
Lophoterges millierei|INV03213|Portugal|BOLD:ABV3881
Calophasia platyptera|INV02808|Portugal|BOLD:AAM0605
Calophasia platyptera|INV01419|Portugal|BOLD:AAM0605
Calophasia almoravida|INV01484|Portugal|BOLD:AAM0606
Calophasia almoravida|INV03245|Portugal|BOLD:AAM0606
Nycteola revayana|INV08615|Portugal|BOLD:AAB8993
Nycteola columbana|INV03077|Portugal|BOLD:AAB8994
Acontia trabealis|INV08978|Portugal|BOLD:AAH9112
Acontia lucida|INV01054|Portugal|BOLD:AAD6258
Acontia lucida|INV01417|Portugal|BOLD:AAD6258
Acontia lucida|INV01425|Portugal|BOLD:AAD6258
Acontia lucida|INV03127|Portugal|BOLD:AAD6258
Mythimna riparia|INV00996|Portugal|BOLD:AAK8682
Mythimna riparia|INV02006|Portugal|BOLD:AAK8682
Mythimna riparia|INV03130|Portugal|BOLD:AAK8682
Heliothis peltigera|INV01422|Portugal|BOLD:AAC6990
Heliothis peltigera|INV03231|Portugal|BOLD:AAC6990
Heliothis peltigera|INV01034|Portugal|BOLD:AAC6990
Heliothis incarnata|INV01415|Portugal|BOLD:ABX4666
Heliothis incarnata|INV01401|Portugal|BOLD:ABX4666
Heliothis incarnata|INV03000|Portugal|BOLD:ABX4666
Helicoverpa armigera|INV01805|Portugal|BOLD:AAA5223
Helicoverpa armigera|INV03991|Portugal|BOLD:AAA5223
Polypogon plumigeralis|INV04006|Portugal|BOLD:AAI4196
Polypogon plumigeralis|INV06900|Portugal|BOLD:AAI4196
Nodaria nodosalis|INV03149|Portugal|BOLD:AAK3749
Nodaria nodosalis|INV07056|Portugal|BOLD:AAK3749
Chloantha hyperici|INV04635|Portugal|BOLD:AAD4758
Chloantha hyperici|INV00868|Portugal|BOLD:AAD4758
Chloantha hyperici|INV02981|Portugal|BOLD:AAD4758
Herminia grisealis|INV08773|Portugal|BOLD:AAC3337
Herminia tarsipennalis|INV10338|Portugal|BOLD:AAC1538
Tholera decimalis|INV01783|Portugal|BOLD:AAC9682
Tholera decimalis|INV04061|Portugal|BOLD:AAC9682
Tyta luctuosa|INV04085|Portugal|BOLD:AAD5088
Tyta luctuosa|INV00875|Portugal|BOLD:AAD5088
Tyta luctuosa|INV01421|Portugal|BOLD:AAD5088
Tyta luctuosa|INV01411|Portugal|BOLD:AAD5088
Tyta luctuosa|INV03122|Portugal|BOLD:AAD5088
Nyctobrya muralis|INV03965|Portugal|BOLD:AAF1445
Nyctobrya muralis|INV01918|Portugal|BOLD:AAF1445
Nyctobrya muralis|INV04013|Portugal|BOLD:AAF1445
Elaphria venustula|INV01320|Portugal|BOLD:AAE0188
Elaphria venustula|INV09002|Portugal|BOLD:AAE0188
Chilodes maritima|INV08901|Portugal|BOLD:ACE6941
Proxenus hospes|INV03133|Portugal|BOLD:AAG5813
Proxenus hospes|INV01994|Portugal|BOLD:AAG5813
Hoplodrina ambigua|INV02983|Portugal|BOLD:AAB0832
Hoplodrina ambigua|INV01789|Portugal|BOLD:AAB0832
Hoplodrina ambigua|INV01037|Portugal|BOLD:AAB0832
Hoplodrina octogenaria|INV05326|Portugal|BOLD:AAB4763
Hoplodrina octogenaria|INV05432|Portugal|BOLD:AAB4763
Hoplodrina octogenaria|INV01331|Portugal|BOLD:AAB4763
Hoplodrina blanda|INV04178|Portugal|BOLD:AAC0362
Hoplodrina blanda|INV03988|Portugal|BOLD:AAC0362
Caradrina aspersa|INV01016|Portugal|BOLD:ABZ5809
Caradrina clavipalpis|INV02452|Portugal|BOLD:AAB6999
Caradrina clavipalpis|INV04470|Portugal|BOLD:AAB6999
Caradrina clavipalpis|INV02836|Portugal|BOLD:AAB6999
Caradrina flavirena|INV02442|Portugal|BOLD:AAB7000
Caradrina flavirena|INV02802|Portugal|BOLD:AAB7000
Caradrina flavirena|INV03217|Portugal|BOLD:AAB7000
Caradrina selini|INV01165|Portugal|BOLD:ABZ7109
Caradrina selini|INV03224|Portugal|BOLD:ABZ7109
Caradrina selini|INV01095|Portugal|BOLD:ABZ7109
Caradrina proxima|INV00863|Portugal|BOLD:ABW9317
Caradrina germainii|INV06867|Portugal|BOLD:AAY7842
Caradrina germainii|INV07788|Portugal|BOLD:AAY7842
Polyphaenis sericata|INV00650|Portugal|BOLD:AAL0181
Thalpophila vitalba|INV01292|Portugal|BOLD:ACF3547
Stilbia andalusiaca|INV01905|Portugal|BOLD:ABV4364
Stilbia anomala|INV10208|Portugal|BOLD:ACL5539
Amphipyra tragopoginis|INV03669|Portugal|BOLD:AAB3277
Amphipyra tragopoginis|INV03996|Portugal|BOLD:AAB3277
Amphipyra tragopoginis|INV10247|Portugal|BOLD:AAB3277
Amphipyra pyramidea|INV01870|Portugal|BOLD:AAB4107
Amphipyra pyramidea|INV03964|Portugal|BOLD:AAB4107
Amphipyra pyramidea|INV10290|Portugal|BOLD:AAB4107
Bryophila ravula|INV02637|Portugal|BOLD:ADT6451
Bryophila ravula|INV01051|Portugal|BOLD:ADT6451
Bryophila ravula|INV01212|Portugal|BOLD:ADT6451
Diarsia brunnea|INV00691|Portugal|BOLD:AAD6686
Diarsia guadarramensis|INV03383|Portugal|BOLD:AAY8159
Chersotis oreina|INV04066|Portugal|BOLD:AAJ0523
Peridroma saucia|INV02404|Portugal|BOLD:AAA6377
Peridroma saucia|INV02869|Portugal|BOLD:AAA6377
Peridroma saucia|INV05343|Portugal|BOLD:AAA6377
Agrotis puta|INV02825|Portugal|BOLD:AAB9164
Agrotis puta|INV02003|Portugal|BOLD:AAB9164
Agrotis puta|INV01828|Portugal|BOLD:AAB9164
Agrotis catalaunensis|INV02008|Portugal|BOLD:ABZ4331
Agrotis exclamationis|INV00625|Portugal|BOLD:AAB9113
Agrotis exclamationis|INV02367|Portugal|BOLD:AAB9113
Agrotis exclamationis|INV02456|Portugal|BOLD:AAB9113
Agrotis segetum|INV01832|Portugal|BOLD:AAC3884
Agrotis segetum|INV02432|Portugal|BOLD:AAC3884
Agrotis segetum|INV02870|Portugal|BOLD:AAC3884
Agrotis ipsilon|INV01336|Portugal|BOLD:AAA3364

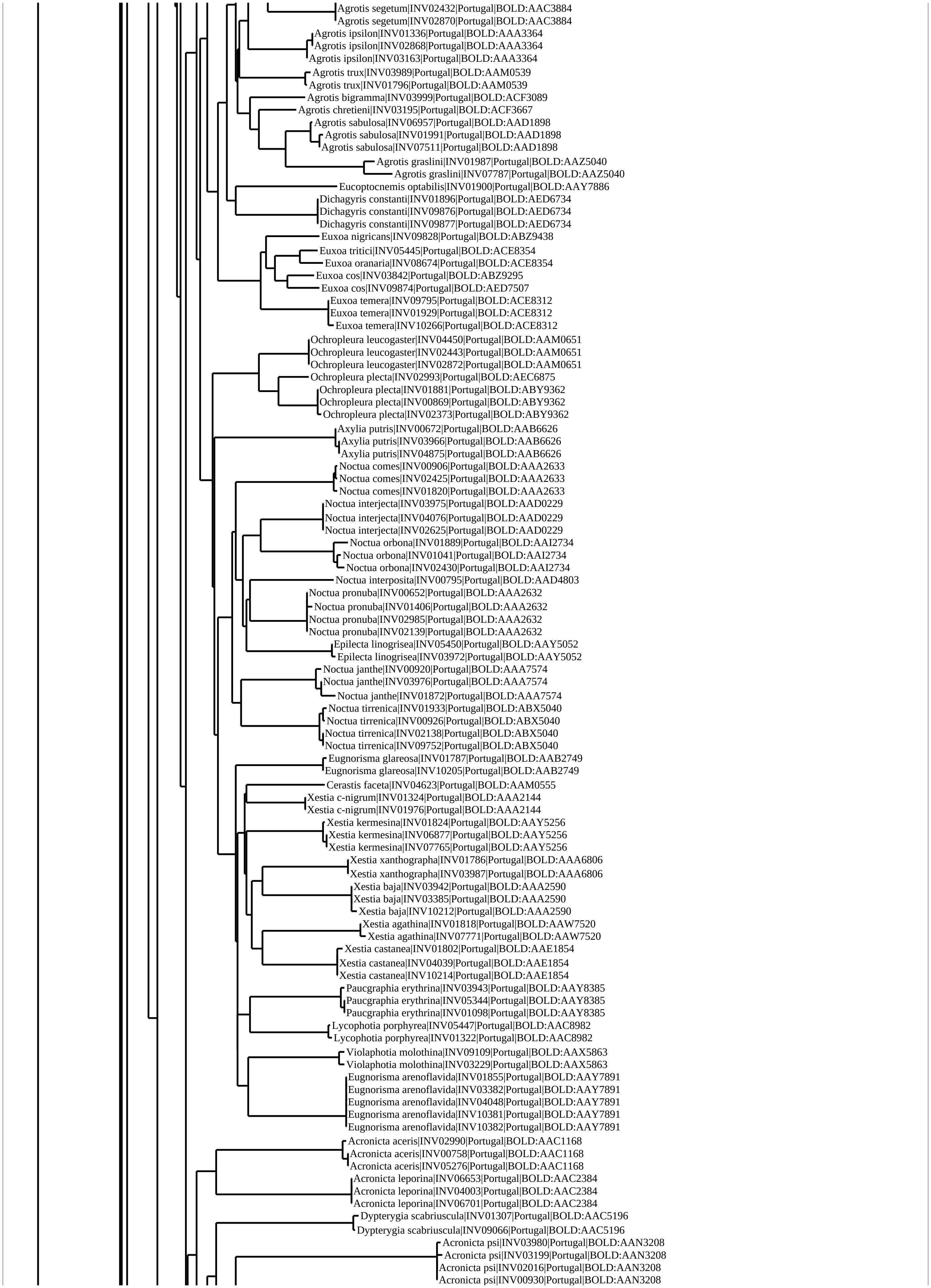

Agrotis segetum|INV02432|Portugal|BOLD:AAC3884
Agrotis segetum|INV02870|Portugal|BOLD:AAC3884
Agrotis ipsilon|INV01336|Portugal|BOLD:AAA3364
Agrotis ipsilon|INV02868|Portugal|BOLD:AAA3364
Agrotis ipsilon|INV03163|Portugal|BOLD:AAA3364
Agrotis trux|INV03989|Portugal|BOLD:AAM0539
Agrotis trux|INV01796|Portugal|BOLD:AAM0539
Agrotis bigramma|INV03999|Portugal|BOLD:ACF3089
Agrotis chretieni|INV03195|Portugal|BOLD:ACF3667
Agrotis sabulosa|INV06957|Portugal|BOLD:AAD1898
Agrotis sabulosa|INV01991|Portugal|BOLD:AAD1898
Agrotis sabulosa|INV07511|Portugal|BOLD:AAD1898
Agrotis graslini|INV01987|Portugal|BOLD:AAZ5040
Agrotis graslini|INV07787|Portugal|BOLD:AAZ5040
Eucoptocnemis optabilis|INV01900|Portugal|BOLD:AAY7886
Dichagyris constanti|INV01896|Portugal|BOLD:AED6734
Dichagyris constanti|INV09876|Portugal|BOLD:AED6734
Dichagyris constanti|INV09877|Portugal|BOLD:AED6734
Euxoa nigricans|INV09828|Portugal|BOLD:ABZ9438
Euxoa tritici|INV05445|Portugal|BOLD:ACE8354
Euxoa oranaria|INV08674|Portugal|BOLD:ACE8354
Euxoa cos|INV03842|Portugal|BOLD:ABZ9295
Euxoa cos|INV09874|Portugal|BOLD:AED7507
Euxoa temera|INV09795|Portugal|BOLD:ACE8312
Euxoa temera|INV01929|Portugal|BOLD:ACE8312
Euxoa temera|INV10266|Portugal|BOLD:ACE8312
Ochropleura leucogaster|INV04450|Portugal|BOLD:AAM0651
Ochropleura leucogaster|INV02443|Portugal|BOLD:AAM0651
Ochropleura leucogaster|INV02872|Portugal|BOLD:AAM0651
Ochropleura plecta|INV02993|Portugal|BOLD:AEC6875
Ochropleura plecta|INV01881|Portugal|BOLD:ABY9362
Ochropleura plecta|INV00869|Portugal|BOLD:ABY9362
Ochropleura plecta|INV02373|Portugal|BOLD:ABY9362
Axylia putris|INV00672|Portugal|BOLD:AAB6626
Axylia putris|INV03966|Portugal|BOLD:AAB6626
Axylia putris|INV04875|Portugal|BOLD:AAB6626
Noctua comes|INV00906|Portugal|BOLD:AAA2633
Noctua comes|INV02425|Portugal|BOLD:AAA2633
Noctua comes|INV01820|Portugal|BOLD:AAA2633
Noctua interjecta|INV03975|Portugal|BOLD:AAD0229
Noctua interjecta|INV04076|Portugal|BOLD:AAD0229
Noctua interjecta|INV02625|Portugal|BOLD:AAD0229
Noctua orbona|INV01889|Portugal|BOLD:AAI2734
Noctua orbona|INV01041|Portugal|BOLD:AAI2734
Noctua orbona|INV02430|Portugal|BOLD:AAI2734
Noctua interposita|INV00795|Portugal|BOLD:AAD4803
Noctua pronuba|INV00652|Portugal|BOLD:AAA2632
Noctua pronuba|INV01406|Portugal|BOLD:AAA2632
Noctua pronuba|INV02985|Portugal|BOLD:AAA2632
Noctua pronuba|INV02139|Portugal|BOLD:AAA2632
Epilecta linogrisea|INV05450|Portugal|BOLD:AAY5052
Epilecta linogrisea|INV03972|Portugal|BOLD:AAY5052
Noctua janthe|INV00920|Portugal|BOLD:AAA7574
Noctua janthe|INV03976|Portugal|BOLD:AAA7574
Noctua janthe|INV01872|Portugal|BOLD:AAA7574
Noctua tirrenica|INV01933|Portugal|BOLD:ABX5040
Noctua tirrenica|INV00926|Portugal|BOLD:ABX5040
Noctua tirrenica|INV02138|Portugal|BOLD:ABX5040
Noctua tirrenica|INV09752|Portugal|BOLD:ABX5040
Eugnorisma glareosa|INV01787|Portugal|BOLD:AAB2749
Eugnorisma glareosa|INV10205|Portugal|BOLD:AAB2749
Cerastis faceta|INV04623|Portugal|BOLD:AAM0555
Xestia c-nigrum|INV01324|Portugal|BOLD:AAA2144
Xestia c-nigrum|INV01976|Portugal|BOLD:AAA2144
Xestia kermesina|INV01824|Portugal|BOLD:AAY5256
Xestia kermesina|INV06877|Portugal|BOLD:AAY5256
Xestia kermesina|INV07765|Portugal|BOLD:AAY5256
Xestia xanthographa|INV01786|Portugal|BOLD:AAA6806
Xestia xanthographa|INV03987|Portugal|BOLD:AAA6806
Xestia baja|INV03942|Portugal|BOLD:AAA2590
Xestia baja|INV03385|Portugal|BOLD:AAA2590
Xestia baja|INV10212|Portugal|BOLD:AAA2590
Xestia agathina|INV01818|Portugal|BOLD:AAW7520
Xestia agathina|INV07771|Portugal|BOLD:AAW7520
Xestia castanea|INV01802|Portugal|BOLD:AAE1854
Xestia castanea|INV04039|Portugal|BOLD:AAE1854
Xestia castanea|INV10214|Portugal|BOLD:AAE1854
Paucgraphia erythrina|INV03943|Portugal|BOLD:AAY8385
Paucgraphia erythrina|INV05344|Portugal|BOLD:AAY8385
Paucgraphia erythrina|INV01098|Portugal|BOLD:AAY8385
Lycophotia porphyrea|INV05447|Portugal|BOLD:AAC8982
Lycophotia porphyrea|INV01322|Portugal|BOLD:AAC8982
Violaphotia molothina|INV09109|Portugal|BOLD:AAX5863
Violaphotia molothina|INV03229|Portugal|BOLD:AAX5863
Eugnorisma arenoflavida|INV01855|Portugal|BOLD:AAY7891
Eugnorisma arenoflavida|INV03382|Portugal|BOLD:AAY7891
Eugnorisma arenoflavida|INV04048|Portugal|BOLD:AAY7891
Eugnorisma arenoflavida|INV10381|Portugal|BOLD:AAY7891
Eugnorisma arenoflavida|INV10382|Portugal|BOLD:AAY7891
Acronicta aceris|INV02990|Portugal|BOLD:AAC1168
Acronicta aceris|INV00758|Portugal|BOLD:AAC1168
Acronicta aceris|INV05276|Portugal|BOLD:AAC1168
Acronicta leporina|INV06653|Portugal|BOLD:AAC2384
Acronicta leporina|INV04003|Portugal|BOLD:AAC2384
Acronicta leporina|INV06701|Portugal|BOLD:AAC2384
Dypterygia scabriuscula|INV01307|Portugal|BOLD:AAC5196
Dypterygia scabriuscula|INV09066|Portugal|BOLD:AAC5196
Acronicta psi|INV03980|Portugal|BOLD:AAN3208
Acronicta psi|INV03199|Portugal|BOLD:AAN3208
Acronicta psi|INV02016|Portugal|BOLD:AAN3208
Acronicta psi|INV00930|Portugal|BOLD:AAN3208

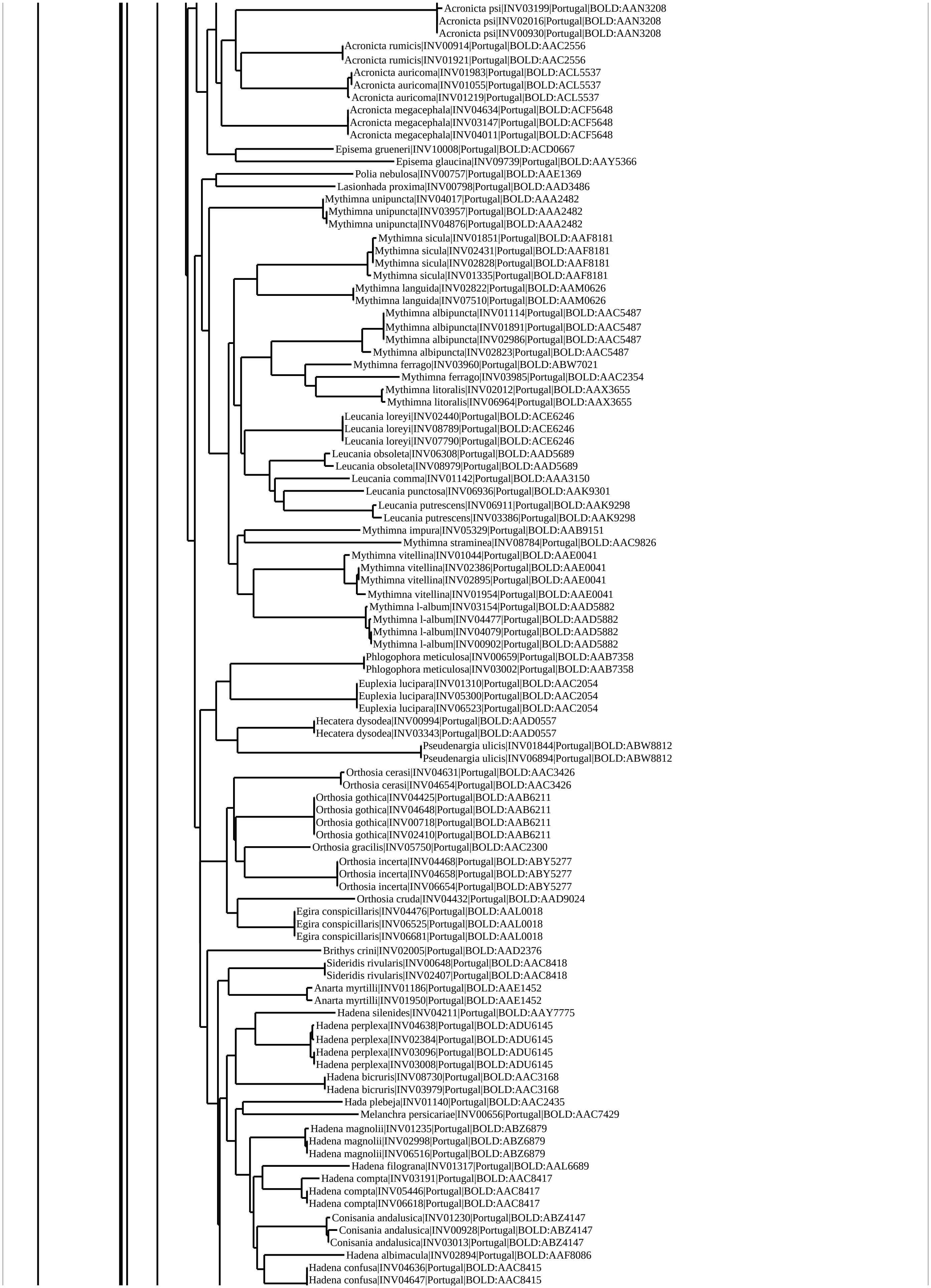
Acronicta psi|INV03199|Portugal|BOLD:AAN3208
Acronicta psi|INV02016|Portugal|BOLD:AAN3208
Acronicta psi|INV00930|Portugal|BOLD:AAN3208
Acronicta rumicis|INV00914|Portugal|BOLD:AAC2556
Acronicta rumicis|INV01921|Portugal|BOLD:AAC2556
Acronicta auricoma|INV01983|Portugal|BOLD:ACL5537
Acronicta auricoma|INV01055|Portugal|BOLD:ACL5537
Acronicta auricoma|INV01219|Portugal|BOLD:ACL5537
Acronicta megacephala|INV04634|Portugal|BOLD:ACF5648
Acronicta megacephala|INV03147|Portugal|BOLD:ACF5648
Acronicta megacephala|INV04011|Portugal|BOLD:ACF5648
Episema grueneri|INV10008|Portugal|BOLD:ACD0667
Episema glaucina|INV09739|Portugal|BOLD:AAY5366
Polia nebulosa|INV00757|Portugal|BOLD:AAE1369
Lasionhada proxima|INV00798|Portugal|BOLD:AAD3486
Mythimna unipuncta|INV04017|Portugal|BOLD:AAA2482
Mythimna unipuncta|INV03957|Portugal|BOLD:AAA2482
Mythimna unipuncta|INV04876|Portugal|BOLD:AAA2482
Mythimna sicula|INV01851|Portugal|BOLD:AAF8181
Mythimna sicula|INV02431|Portugal|BOLD:AAF8181
Mythimna sicula|INV02828|Portugal|BOLD:AAF8181
Mythimna sicula|INV01335|Portugal|BOLD:AAF8181
Mythimna languida|INV02822|Portugal|BOLD:AAM0626
Mythimna languida|INV07510|Portugal|BOLD:AAM0626
Mythimna albipuncta|INV01114|Portugal|BOLD:AAC5487
Mythimna albipuncta|INV01891|Portugal|BOLD:AAC5487
Mythimna albipuncta|INV02986|Portugal|BOLD:AAC5487
Mythimna albipuncta|INV02823|Portugal|BOLD:AAC5487
Mythimna ferrago|INV03960|Portugal|BOLD:ABW7021
Mythimna ferrago|INV03985|Portugal|BOLD:AAC2354
Mythimna litoralis|INV02012|Portugal|BOLD:AAX3655
Mythimna litoralis|INV06964|Portugal|BOLD:AAX3655
Leucania loreyi|INV02440|Portugal|BOLD:ACE6246
Leucania loreyi|INV08789|Portugal|BOLD:ACE6246
Leucania loreyi|INV07790|Portugal|BOLD:ACE6246
Leucania obsoleta|INV06308|Portugal|BOLD:AAD5689
Leucania obsoleta|INV08979|Portugal|BOLD:AAD5689
Leucania comma|INV01142|Portugal|BOLD:AAA3150
Leucania punctosa|INV06936|Portugal|BOLD:AAK9301
Leucania putrescens|INV06911|Portugal|BOLD:AAK9298
Leucania putrescens|INV03386|Portugal|BOLD:AAK9298
Mythimna impura|INV05329|Portugal|BOLD:AAB9151
Mythimna straminea|INV08784|Portugal|BOLD:AAC9826
Mythimna vitellina|INV01044|Portugal|BOLD:AAE0041
Mythimna vitellina|INV02386|Portugal|BOLD:AAE0041
Mythimna vitellina|INV02895|Portugal|BOLD:AAE0041
Mythimna vitellina|INV01954|Portugal|BOLD:AAE0041
Mythimna l-album|INV03154|Portugal|BOLD:AAD5882
Mythimna l-album|INV04477|Portugal|BOLD:AAD5882
Mythimna l-album|INV04079|Portugal|BOLD:AAD5882
Mythimna l-album|INV00902|Portugal|BOLD:AAD5882
Phlogophora meticulosa|INV00659|Portugal|BOLD:AAB7358
Phlogophora meticulosa|INV03002|Portugal|BOLD:AAB7358
Euplexia lucipara|INV01310|Portugal|BOLD:AAC2054
Euplexia lucipara|INV05300|Portugal|BOLD:AAC2054
Euplexia lucipara|INV06523|Portugal|BOLD:AAC2054
Hecatera dysodea|INV00994|Portugal|BOLD:AAD0557
Hecatera dysodea|INV03343|Portugal|BOLD:AAD0557
Pseudenargia ulicis|INV01844|Portugal|BOLD:ABW8812
Pseudenargia ulicis|INV06894|Portugal|BOLD:ABW8812
Orthosia cerasi|INV04631|Portugal|BOLD:AAC3426
Orthosia cerasi|INV04654|Portugal|BOLD:AAC3426
Orthosia gothica|INV04425|Portugal|BOLD:AAB6211
Orthosia gothica|INV04648|Portugal|BOLD:AAB6211
Orthosia gothica|INV00718|Portugal|BOLD:AAB6211
Orthosia gothica|INV02410|Portugal|BOLD:AAB6211
Orthosia gracilis|INV05750|Portugal|BOLD:AAC2300
Orthosia incerta|INV04468|Portugal|BOLD:ABY5277
Orthosia incerta|INV04658|Portugal|BOLD:ABY5277
Orthosia incerta|INV06654|Portugal|BOLD:ABY5277
Orthosia cruda|INV04432|Portugal|BOLD:AAD9024
Egira conspicillaris|INV04476|Portugal|BOLD:AAL0018
Egira conspicillaris|INV06525|Portugal|BOLD:AAL0018
Egira conspicillaris|INV06681|Portugal|BOLD:AAL0018
Brithys crini|INV02005|Portugal|BOLD:AAD2376
Sideridis rivularis|INV00648|Portugal|BOLD:AAC8418
Sideridis rivularis|INV02407|Portugal|BOLD:AAC8418
Anarta myrtilli|INV01186|Portugal|BOLD:AAE1452
Anarta myrtilli|INV01950|Portugal|BOLD:AAE1452
Hadena silenides|INV04211|Portugal|BOLD:AAY7775
Hadena perplexa|INV04638|Portugal|BOLD:ADU6145
Hadena perplexa|INV02384|Portugal|BOLD:ADU6145
Hadena perplexa|INV03096|Portugal|BOLD:ADU6145
Hadena perplexa|INV03008|Portugal|BOLD:ADU6145
Hadena bicruris|INV08730|Portugal|BOLD:AAC3168
Hadena bicruris|INV03979|Portugal|BOLD:AAC3168
Hada plebeja|INV01140|Portugal|BOLD:AAC2435
Melanchra persicariae|INV00656|Portugal|BOLD:AAC7429
Hadena magnolii|INV01235|Portugal|BOLD:ABZ6879
Hadena magnolii|INV02998|Portugal|BOLD:ABZ6879
Hadena magnolii|INV06516|Portugal|BOLD:ABZ6879
Hadena filograna|INV01317|Portugal|BOLD:AAL6689
Hadena compta|INV03191|Portugal|BOLD:AAC8417
Hadena compta|INV05446|Portugal|BOLD:AAC8417
Hadena compta|INV06618|Portugal|BOLD:AAC8417
Conisania andalusica|INV01230|Portugal|BOLD:ABZ4147
Conisania andalusica|INV00928|Portugal|BOLD:ABZ4147
Conisania andalusica|INV03013|Portugal|BOLD:ABZ4147
Hadena albimacula|INV02894|Portugal|BOLD:AAF8086
Hadena confusa|INV04636|Portugal|BOLD:AAC8415
Hadena confusa|INV04647|Portugal|BOLD:AAC8415

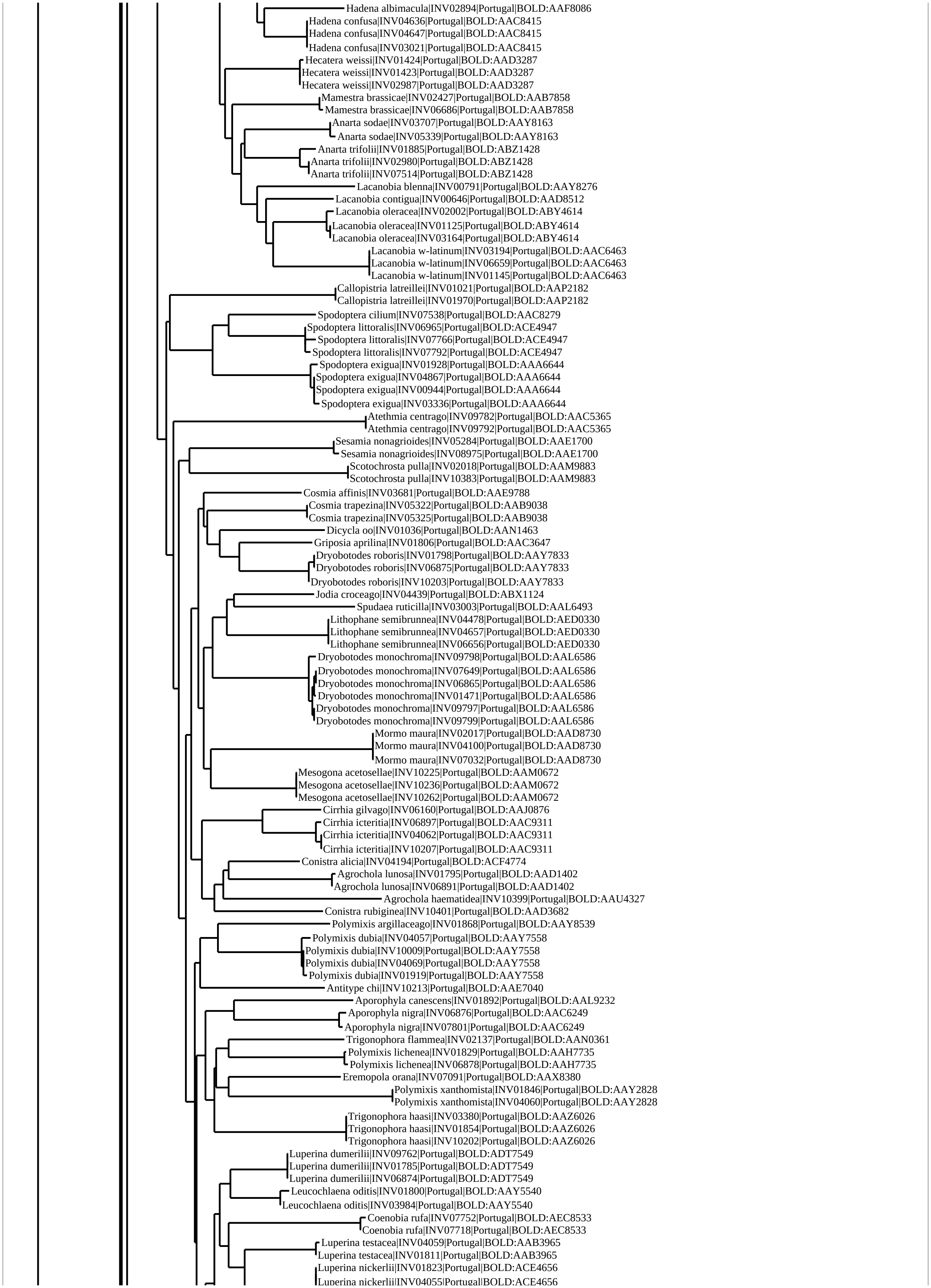
Hadena albimacula|INV02894|Portugal|BOLD:AAF8086
Hadena confusa|INV04636|Portugal|BOLD:AAC8415
Hadena confusa|INV04647|Portugal|BOLD:AAC8415
Hadena confusa|INV03021|Portugal|BOLD:AAC8415
Hecatera weissi|INV01424|Portugal|BOLD:AAD3287
Hecatera weissi|INV01423|Portugal|BOLD:AAD3287
Hecatera weissi|INV02987|Portugal|BOLD:AAD3287
Mamestra brassicae|INV02427|Portugal|BOLD:AAB7858
Mamestra brassicae|INV06686|Portugal|BOLD:AAB7858
Anarta sodae|INV03707|Portugal|BOLD:AAY8163
Anarta sodae|INV05339|Portugal|BOLD:AAY8163
Anarta trifolii|INV01885|Portugal|BOLD:ABZ1428
Anarta trifolii|INV02980|Portugal|BOLD:ABZ1428
Anarta trifolii|INV07514|Portugal|BOLD:ABZ1428
Lacanobia blenna|INV00791|Portugal|BOLD:AAY8276
Lacanobia contigua|INV00646|Portugal|BOLD:AAD8512
Lacanobia oleracea|INV02002|Portugal|BOLD:ABY4614
Lacanobia oleracea|INV01125|Portugal|BOLD:ABY4614
Lacanobia oleracea|INV03164|Portugal|BOLD:ABY4614
Lacanobia w-latinum|INV03194|Portugal|BOLD:AAC6463
Lacanobia w-latinum|INV06659|Portugal|BOLD:AAC6463
Lacanobia w-latinum|INV01145|Portugal|BOLD:AAC6463
Callopistria latreillei|INV01021|Portugal|BOLD:AAP2182
Callopistria latreillei|INV01970|Portugal|BOLD:AAP2182
Spodoptera cilium|INV07538|Portugal|BOLD:AAC8279
Spodoptera littoralis|INV06965|Portugal|BOLD:ACE4947
Spodoptera littoralis|INV07766|Portugal|BOLD:ACE4947
Spodoptera littoralis|INV07792|Portugal|BOLD:ACE4947
Spodoptera exigua|INV01928|Portugal|BOLD:AAA6644
Spodoptera exigua|INV04867|Portugal|BOLD:AAA6644
Spodoptera exigua|INV00944|Portugal|BOLD:AAA6644
Spodoptera exigua|INV03336|Portugal|BOLD:AAA6644
Atethmia centrago|INV09782|Portugal|BOLD:AAC5365
Atethmia centrago|INV09792|Portugal|BOLD:AAC5365
Sesamia nonagrioides|INV05284|Portugal|BOLD:AAE1700
Sesamia nonagrioides|INV08975|Portugal|BOLD:AAE1700
Scotochrosta pulla|INV02018|Portugal|BOLD:AAM9883
Scotochrosta pulla|INV10383|Portugal|BOLD:AAM9883
Cosmia affinis|INV03681|Portugal|BOLD:AAE9788
Cosmia trapezina|INV05322|Portugal|BOLD:AAB9038
Cosmia trapezina|INV05325|Portugal|BOLD:AAB9038
Dicycla oo|INV01036|Portugal|BOLD:AAN1463
Griposia aprilina|INV01806|Portugal|BOLD:AAC3647
Dryobotodes roboris|INV01798|Portugal|BOLD:AAY7833
Dryobotodes roboris|INV06875|Portugal|BOLD:AAY7833
Dryobotodes roboris|INV10203|Portugal|BOLD:AAY7833
Jodia croceago|INV04439|Portugal|BOLD:ABX1124
Spudaea ruticilla|INV03003|Portugal|BOLD:AAL6493
Lithophane semibrunnea|INV04478|Portugal|BOLD:AED0330
Lithophane semibrunnea|INV04657|Portugal|BOLD:AED0330
Lithophane semibrunnea|INV06656|Portugal|BOLD:AED0330
Dryobotodes monochroma|INV09798|Portugal|BOLD:AAL6586
Dryobotodes monochroma|INV07649|Portugal|BOLD:AAL6586
Dryobotodes monochroma|INV06865|Portugal|BOLD:AAL6586
Dryobotodes monochroma|INV01471|Portugal|BOLD:AAL6586
Dryobotodes monochroma|INV09797|Portugal|BOLD:AAL6586
Dryobotodes monochroma|INV09799|Portugal|BOLD:AAL6586
Mormo maura|INV02017|Portugal|BOLD:AAD8730
Mormo maura|INV04100|Portugal|BOLD:AAD8730
Mormo maura|INV07032|Portugal|BOLD:AAD8730
Mesogona acetosellae|INV10225|Portugal|BOLD:AAM0672
Mesogona acetosellae|INV10236|Portugal|BOLD:AAM0672
Mesogona acetosellae|INV10262|Portugal|BOLD:AAM0672
Cirrhia gilvago|INV06160|Portugal|BOLD:AAJ0876
Cirrhia icteritia|INV06897|Portugal|BOLD:AAC9311
Cirrhia icteritia|INV04062|Portugal|BOLD:AAC9311
Cirrhia icteritia|INV10207|Portugal|BOLD:AAC9311
Conistra alicia|INV04194|Portugal|BOLD:ACF4774
Agrochola lunosa|INV01795|Portugal|BOLD:AAD1402
Agrochola lunosa|INV06891|Portugal|BOLD:AAD1402
Agrochola haematidea|INV10399|Portugal|BOLD:AAU4327
Conistra rubiginea|INV10401|Portugal|BOLD:AAD3682
Polymixis argillaceago|INV01868|Portugal|BOLD:AAY8539
Polymixis dubia|INV04057|Portugal|BOLD:AAY7558
Polymixis dubia|INV10009|Portugal|BOLD:AAY7558
Polymixis dubia|INV04069|Portugal|BOLD:AAY7558
Polymixis dubia|INV01919|Portugal|BOLD:AAY7558
Antitype chi|INV10213|Portugal|BOLD:AAE7040
Aporophyla canescens|INV01892|Portugal|BOLD:AAL9232
Aporophyla nigra|INV06876|Portugal|BOLD:AAC6249
Aporophyla nigra|INV07801|Portugal|BOLD:AAC6249
Trigonophora flammea|INV02137|Portugal|BOLD:AAN0361
Polymixis lichenea|INV01829|Portugal|BOLD:AAH7735
Polymixis lichenea|INV06878|Portugal|BOLD:AAH7735
Eremopola orana|INV07091|Portugal|BOLD:AAX8380
Polymixis xanthomista|INV01846|Portugal|BOLD:AAY2828
Polymixis xanthomista|INV04060|Portugal|BOLD:AAY2828
Trigonophora haasi|INV03380|Portugal|BOLD:AAZ6026
Trigonophora haasi|INV01854|Portugal|BOLD:AAZ6026
Trigonophora haasi|INV10202|Portugal|BOLD:AAZ6026
Luperina dumerilii|INV09762|Portugal|BOLD:ADT7549
Luperina dumerilii|INV01785|Portugal|BOLD:ADT7549
Luperina dumerilii|INV06874|Portugal|BOLD:ADT7549
Leucochlaena oditis|INV01800|Portugal|BOLD:AAY5540
Leucochlaena oditis|INV03984|Portugal|BOLD:AAY5540
Coenobia rufa|INV07752|Portugal|BOLD:AEC8533
Coenobia rufa|INV07718|Portugal|BOLD:AEC8533
Luperina testacea|INV04059|Portugal|BOLD:AAB3965
Luperina testacea|INV01811|Portugal|BOLD:AAB3965
Luperina nickerlii|INV01823|Portugal|BOLD:ACE4656
Luperina nickerlii|INV04055|Portugal|BOLD:ACE4656

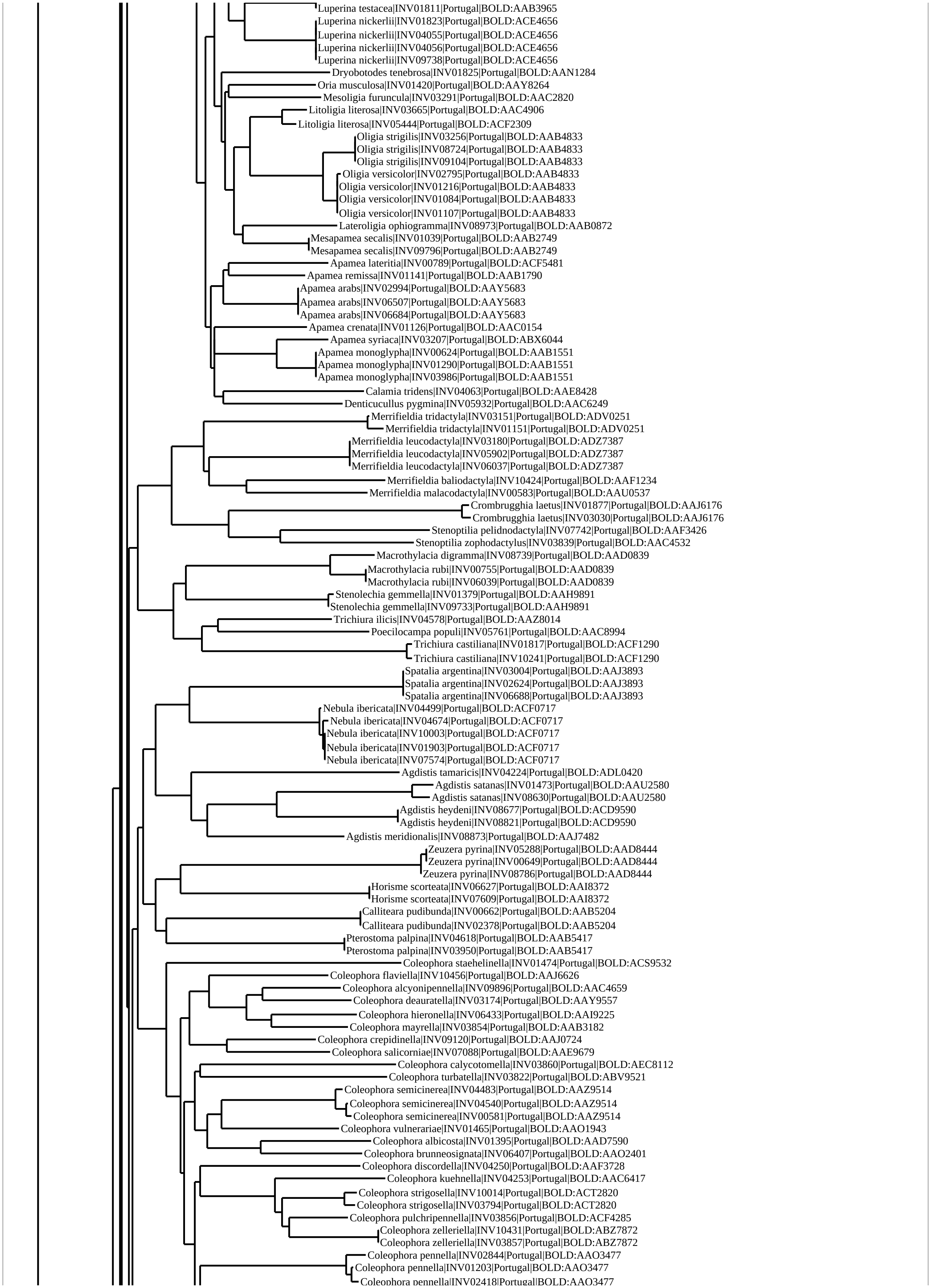
Luperina testacea|INV01811|Portugal|BOLD:AAB3965
Luperina nickerlii|INV01823|Portugal|BOLD:ACE4656
Luperina nickerlii|INV04055|Portugal|BOLD:ACE4656
Luperina nickerlii|INV04056|Portugal|BOLD:ACE4656
Luperina nickerlii|INV09738|Portugal|BOLD:ACE4656
Dryobotodes tenebrosa|INV01825|Portugal|BOLD:AAN1284
Oria musculosa|INV01420|Portugal|BOLD:AAY8264
Mesoligia furuncula|INV03291|Portugal|BOLD:AAC2820
Litoligia literosa|INV03665|Portugal|BOLD:AAC4906
Litoligia literosa|INV05444|Portugal|BOLD:ACF2309
Oligia strigilis|INV03256|Portugal|BOLD:AAB4833
Oligia strigilis|INV08724|Portugal|BOLD:AAB4833
Oligia strigilis|INV09104|Portugal|BOLD:AAB4833
Oligia versicolor|INV02795|Portugal|BOLD:AAB4833
Oligia versicolor|INV01216|Portugal|BOLD:AAB4833
Oligia versicolor|INV01084|Portugal|BOLD:AAB4833
Oligia versicolor|INV01107|Portugal|BOLD:AAB4833
Lateroligia ophiogramma|INV08973|Portugal|BOLD:AAB0872
Mesapamea secalis|INV01039|Portugal|BOLD:AAB2749
Mesapamea secalis|INV09796|Portugal|BOLD:AAB2749
Apamea lateritia|INV00789|Portugal|BOLD:ACF5481
Apamea remissa|INV01141|Portugal|BOLD:AAB1790
Apamea arabs|INV02994|Portugal|BOLD:AAY5683
Apamea arabs|INV06507|Portugal|BOLD:AAY5683
Apamea arabs|INV06684|Portugal|BOLD:AAY5683
Apamea crenata|INV01126|Portugal|BOLD:AAC0154
Apamea syriaca|INV03207|Portugal|BOLD:ABX6044
Apamea monoglypha|INV00624|Portugal|BOLD:AAB1551
Apamea monoglypha|INV01290|Portugal|BOLD:AAB1551
Apamea monoglypha|INV03986|Portugal|BOLD:AAB1551
Calamia tridens|INV04063|Portugal|BOLD:AAE8428
Denticucullus pygmina|INV05932|Portugal|BOLD:AAC6249
Merrifieldia tridactyla|INV03151|Portugal|BOLD:ADV0251
Merrifieldia tridactyla|INV01151|Portugal|BOLD:ADV0251
Merrifieldia leucodactyla|INV03180|Portugal|BOLD:ADZ7387
Merrifieldia leucodactyla|INV05902|Portugal|BOLD:ADZ7387
Merrifieldia leucodactyla|INV06037|Portugal|BOLD:ADZ7387
Merrifieldia baliodactyla|INV10424|Portugal|BOLD:AAF1234
Merrifieldia malacodactyla|INV00583|Portugal|BOLD:AAU0537
Crombrugghia laetus|INV01877|Portugal|BOLD:AAJ6176
Crombrugghia laetus|INV03030|Portugal|BOLD:AAJ6176
Stenoptilia pelidnodactyla|INV07742|Portugal|BOLD:AAF3426
Stenoptilia zophodactylus|INV03839|Portugal|BOLD:AAC4532
Macrothylacia digramma|INV08739|Portugal|BOLD:AAD0839
Macrothylacia rubi|INV00755|Portugal|BOLD:AAD0839
Macrothylacia rubi|INV06039|Portugal|BOLD:AAD0839
Stenolechia gemmella|INV01379|Portugal|BOLD:AAH9891
Stenolechia gemmella|INV09733|Portugal|BOLD:AAH9891
Trichiura ilicis|INV04578|Portugal|BOLD:AAZ8014
Poecilocampa populi|INV05761|Portugal|BOLD:AAC8994
Trichiura castiliana|INV01817|Portugal|BOLD:ACF1290
Trichiura castiliana|INV10241|Portugal|BOLD:ACF1290
Spatalia argentina|INV03004|Portugal|BOLD:AAJ3893
Spatalia argentina|INV02624|Portugal|BOLD:AAJ3893
Spatalia argentina|INV06688|Portugal|BOLD:AAJ3893
Nebula ibericata|INV04499|Portugal|BOLD:ACF0717
Nebula ibericata|INV04674|Portugal|BOLD:ACF0717
Nebula ibericata|INV10003|Portugal|BOLD:ACF0717
Nebula ibericata|INV01903|Portugal|BOLD:ACF0717
Nebula ibericata|INV07574|Portugal|BOLD:ACF0717
Agdistis tamaricis|INV04224|Portugal|BOLD:ADL0420
Agdistis satanas|INV01473|Portugal|BOLD:AAU2580
Agdistis satanas|INV08630|Portugal|BOLD:AAU2580
Agdistis heydeni|INV08677|Portugal|BOLD:ACD9590
Agdistis heydeni|INV08821|Portugal|BOLD:ACD9590
Agdistis meridionalis|INV08873|Portugal|BOLD:AAJ7482
Zeuzera pyrina|INV05288|Portugal|BOLD:AAD8444
Zeuzera pyrina|INV00649|Portugal|BOLD:AAD8444
Zeuzera pyrina|INV08786|Portugal|BOLD:AAD8444
Horisme scorteata|INV06627|Portugal|BOLD:AAI8372
Horisme scorteata|INV07609|Portugal|BOLD:AAI8372
Calliteara pudibunda|INV00662|Portugal|BOLD:AAB5204
Calliteara pudibunda|INV02378|Portugal|BOLD:AAB5204
Pterostoma palpina|INV04618|Portugal|BOLD:AAB5417
Pterostoma palpina|INV03950|Portugal|BOLD:AAB5417
Coleophora staehelinella|INV01474|Portugal|BOLD:ACS9532
Coleophora flaviella|INV10456|Portugal|BOLD:AAJ6626
Coleophora alcyonipennella|INV09896|Portugal|BOLD:AAC4659
Coleophora deauratella|INV03174|Portugal|BOLD:AAY9557
Coleophora hieronella|INV06433|Portugal|BOLD:AAI9225
Coleophora mayrella|INV03854|Portugal|BOLD:AAB3182
Coleophora crepidinella|INV09120|Portugal|BOLD:AAJ0724
Coleophora salicorniae|INV07088|Portugal|BOLD:AAE9679
Coleophora calycotomella|INV03860|Portugal|BOLD:AEC8112
Coleophora turbatella|INV03822|Portugal|BOLD:ABV9521
Coleophora semicinerea|INV04483|Portugal|BOLD:AAZ9514
Coleophora semicinerea|INV04540|Portugal|BOLD:AAZ9514
Coleophora semicinerea|INV00581|Portugal|BOLD:AAZ9514
Coleophora vulnerariae|INV01465|Portugal|BOLD:AAO1943
Coleophora albicosta|INV01395|Portugal|BOLD:AAD7590
Coleophora brunneosignata|INV06407|Portugal|BOLD:AAO2401
Coleophora discordella|INV04250|Portugal|BOLD:AAF3728
Coleophora kuehnella|INV04253|Portugal|BOLD:AAC6417
Coleophora strigosella|INV10014|Portugal|BOLD:ACT2820
Coleophora strigosella|INV03794|Portugal|BOLD:ACT2820
Coleophora pulchripennella|INV03856|Portugal|BOLD:ACF4285
Coleophora zelleriella|INV10431|Portugal|BOLD:ABZ7872
Coleophora zelleriella|INV03857|Portugal|BOLD:ABZ7872
Coleophora pennella|INV02844|Portugal|BOLD:AAO3477
Coleophora pennella|INV01203|Portugal|BOLD:AAO3477
Coleophora pennella|INV02418|Portugal|BOLD:AAO3477

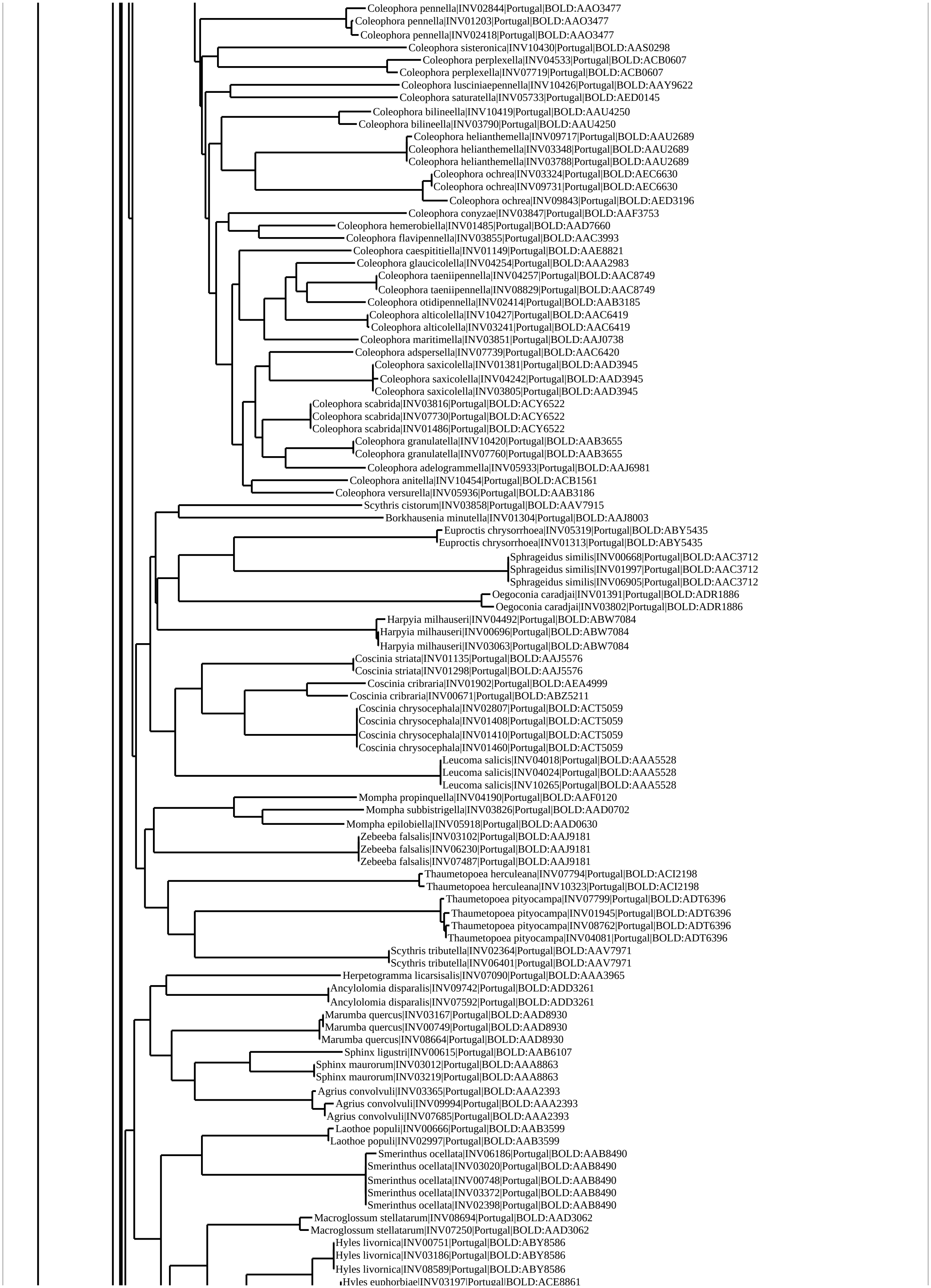
Coleophora pennella|INV02844|Portugal|BOLD:AAO3477
Coleophora pennella|INV01203|Portugal|BOLD:AAO3477
Coleophora pennella|INV02418|Portugal|BOLD:AAO3477
Coleophora sisteronica|INV10430|Portugal|BOLD:AAS0298
Coleophora perplexella|INV04533|Portugal|BOLD:ACB0607
Coleophora perplexella|INV07719|Portugal|BOLD:ACB0607
Coleophora lusciniaepennella|INV10426|Portugal|BOLD:AAY9622
Coleophora saturatella|INV05733|Portugal|BOLD:AED0145
Coleophora bilineella|INV10419|Portugal|BOLD:AAU4250
Coleophora bilineella|INV03790|Portugal|BOLD:AAU4250
Coleophora helianthemella|INV09717|Portugal|BOLD:AAU2689
Coleophora helianthemella|INV03348|Portugal|BOLD:AAU2689
Coleophora helianthemella|INV03788|Portugal|BOLD:AAU2689
Coleophora ochrea|INV03324|Portugal|BOLD:AEC6630
Coleophora ochrea|INV09731|Portugal|BOLD:AEC6630
Coleophora ochrea|INV09843|Portugal|BOLD:AED3196
Coleophora conyzae|INV03847|Portugal|BOLD:AAF3753
Coleophora hemerobiella|INV01485|Portugal|BOLD:AAD7660
Coleophora flavipennella|INV03855|Portugal|BOLD:AAC3993
Coleophora caespititiella|INV01149|Portugal|BOLD:AAE8821
Coleophora glaucicolella|INV04254|Portugal|BOLD:AAA2983
Coleophora taeniipennella|INV04257|Portugal|BOLD:AAC8749
Coleophora taeniipennella|INV08829|Portugal|BOLD:AAC8749
Coleophora otidipennella|INV02414|Portugal|BOLD:AAB3185
Coleophora alticolella|INV10427|Portugal|BOLD:AAC6419
Coleophora alticolella|INV03241|Portugal|BOLD:AAC6419
Coleophora maritimella|INV03851|Portugal|BOLD:AAJ0738
Coleophora adspersella|INV07739|Portugal|BOLD:AAC6420
Coleophora saxicolella|INV01381|Portugal|BOLD:AAD3945
Coleophora saxicolella|INV04242|Portugal|BOLD:AAD3945
Coleophora saxicolella|INV03805|Portugal|BOLD:AAD3945
Coleophora scabrida|INV03816|Portugal|BOLD:ACY6522
Coleophora scabrida|INV07730|Portugal|BOLD:ACY6522
Coleophora scabrida|INV01486|Portugal|BOLD:ACY6522
Coleophora granulatella|INV10420|Portugal|BOLD:AAB3655
Coleophora granulatella|INV07760|Portugal|BOLD:AAB3655
Coleophora adelogrammella|INV05933|Portugal|BOLD:AAJ6981
Coleophora anitella|INV10454|Portugal|BOLD:ACB1561
Coleophora versurella|INV05936|Portugal|BOLD:AAB3186
Scythris cistorum|INV03858|Portugal|BOLD:AAV7915
Borkhausenia minutella|INV01304|Portugal|BOLD:AAJ8003
Euproctis chrysorrhoea|INV05319|Portugal|BOLD:ABY5435
Euproctis chrysorrhoea|INV01313|Portugal|BOLD:ABY5435
Sphrageidus similis|INV00668|Portugal|BOLD:AAC3712
Sphrageidus similis|INV01997|Portugal|BOLD:AAC3712
Sphrageidus similis|INV06905|Portugal|BOLD:AAC3712
Oegoconia caradjai|INV01391|Portugal|BOLD:ADR1886
Oegoconia caradjai|INV03802|Portugal|BOLD:ADR1886
Harpyia milhauseri|INV04492|Portugal|BOLD:ABW7084
Harpyia milhauseri|INV00696|Portugal|BOLD:ABW7084
Harpyia milhauseri|INV03063|Portugal|BOLD:ABW7084
Coscinia striata|INV01135|Portugal|BOLD:AAJ5576
Coscinia striata|INV01298|Portugal|BOLD:AAJ5576
Coscinia cribraria|INV01902|Portugal|BOLD:AEA4999
Coscinia cribraria|INV00671|Portugal|BOLD:ABZ5211
Coscinia chrysocephala|INV02807|Portugal|BOLD:ACT5059
Coscinia chrysocephala|INV01408|Portugal|BOLD:ACT5059
Coscinia chrysocephala|INV01410|Portugal|BOLD:ACT5059
Coscinia chrysocephala|INV01460|Portugal|BOLD:ACT5059
Leucoma salicis|INV04018|Portugal|BOLD:AAA5528
Leucoma salicis|INV04024|Portugal|BOLD:AAA5528
Leucoma salicis|INV10265|Portugal|BOLD:AAA5528
Mompha propinquella|INV04190|Portugal|BOLD:AAF0120
Mompha subbistrigella|INV03826|Portugal|BOLD:AAD0702
Mompha epilobiella|INV05918|Portugal|BOLD:AAD0630
Zebeeba falsalis|INV03102|Portugal|BOLD:AAJ9181
Zebeeba falsalis|INV06230|Portugal|BOLD:AAJ9181
Zebeeba falsalis|INV07487|Portugal|BOLD:AAJ9181
Thaumetopoea herculeana|INV07794|Portugal|BOLD:ACI2198
Thaumetopoea herculeana|INV10323|Portugal|BOLD:ACI2198
Thaumetopoea pityocampa|INV07799|Portugal|BOLD:ADT6396
Thaumetopoea pityocampa|INV01945|Portugal|BOLD:ADT6396
Thaumetopoea pityocampa|INV08762|Portugal|BOLD:ADT6396
Thaumetopoea pityocampa|INV04081|Portugal|BOLD:ADT6396
Scythris tributella|INV02364|Portugal|BOLD:AAV7971
Scythris tributella|INV06401|Portugal|BOLD:AAV7971
Herpetogramma licarsisalis|INV07090|Portugal|BOLD:AAA3965
Ancylolomia disparalis|INV09742|Portugal|BOLD:ADD3261
Ancylolomia disparalis|INV07592|Portugal|BOLD:ADD3261
Marumba quercus|INV03167|Portugal|BOLD:AAD8930
Marumba quercus|INV00749|Portugal|BOLD:AAD8930
Marumba quercus|INV08664|Portugal|BOLD:AAD8930
Sphinx ligustri|INV00615|Portugal|BOLD:AAB6107
Sphinx maurorum|INV03012|Portugal|BOLD:AAA8863
Sphinx maurorum|INV03219|Portugal|BOLD:AAA8863
Agrius convolvuli|INV03365|Portugal|BOLD:AAA2393
Agrius convolvuli|INV09994|Portugal|BOLD:AAA2393
Agrius convolvuli|INV07685|Portugal|BOLD:AAA2393
Laothoe populi|INV00666|Portugal|BOLD:AAB3599
Laothoe populi|INV02997|Portugal|BOLD:AAB3599
Smerinthus ocellata|INV06186|Portugal|BOLD:AAB8490
Smerinthus ocellata|INV03020|Portugal|BOLD:AAB8490
Smerinthus ocellata|INV00748|Portugal|BOLD:AAB8490
Smerinthus ocellata|INV03372|Portugal|BOLD:AAB8490
Smerinthus ocellata|INV02398|Portugal|BOLD:AAB8490
Macroglossum stellatarum|INV08694|Portugal|BOLD:AAD3062
Macroglossum stellatarum|INV07250|Portugal|BOLD:AAD3062
Hyles livornica|INV00751|Portugal|BOLD:ABY8586
Hyles livornica|INV03186|Portugal|BOLD:ABY8586
Hyles livornica|INV08589|Portugal|BOLD:ABY8586
Hyles euphorbiae|INV03197|Portugal|BOLD:ACE8861

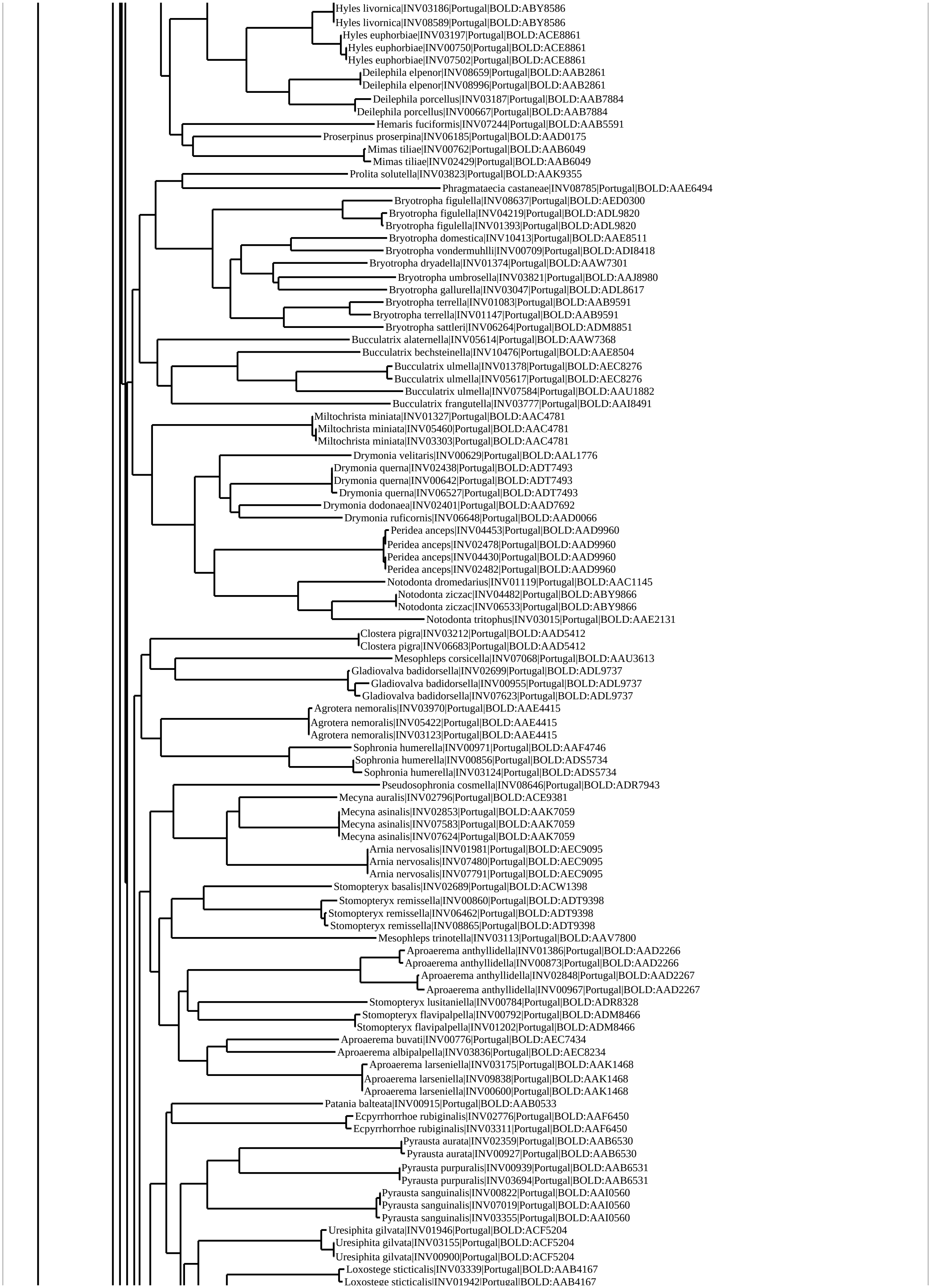
Hyles livornica|INV03186|Portugal|BOLD:ABY8586
Hyles livornica|INV08589|Portugal|BOLD:ABY8586
Hyles euphorbiae|INV03197|Portugal|BOLD:ACE8861
Hyles euphorbiae|INV00750|Portugal|BOLD:ACE8861
Hyles euphorbiae|INV07502|Portugal|BOLD:ACE8861
Deilephila elpenor|INV08659|Portugal|BOLD:AAB2861
Deilephila elpenor|INV08996|Portugal|BOLD:AAB2861
Deilephila porcellus|INV03187|Portugal|BOLD:AAB7884
Deilephila porcellus|INV00667|Portugal|BOLD:AAB7884
Hemaris fuciformis|INV07244|Portugal|BOLD:AAB5591
Proserpinus proserpina|INV06185|Portugal|BOLD:AAD0175
Mimas tiliae|INV00762|Portugal|BOLD:AAB6049
Mimas tiliae|INV02429|Portugal|BOLD:AAB6049
Prolita solutella|INV03823|Portugal|BOLD:AAK9355
Phragmataecia castaneae|INV08785|Portugal|BOLD:AAE6494
Bryotropha figulella|INV08637|Portugal|BOLD:AED0300
Bryotropha figulella|INV04219|Portugal|BOLD:ADL9820
Bryotropha figulella|INV01393|Portugal|BOLD:ADL9820
Bryotropha domestica|INV10413|Portugal|BOLD:AAE8511
Bryotropha vondermuhlli|INV00709|Portugal|BOLD:ADI8418
Bryotropha dryadella|INV01374|Portugal|BOLD:AAW7301
Bryotropha umbrosella|INV03821|Portugal|BOLD:AAJ8980
Bryotropha gallurella|INV03047|Portugal|BOLD:ADL8617
Bryotropha terrella|INV01083|Portugal|BOLD:AAB9591
Bryotropha terrella|INV01147|Portugal|BOLD:AAB9591
Bryotropha sattleri|INV06264|Portugal|BOLD:ADM8851
Bucculatrix alaternella|INV05614|Portugal|BOLD:AAW7368
Bucculatrix bechsteinella|INV10476|Portugal|BOLD:AAE8504
Bucculatrix ulmella|INV01378|Portugal|BOLD:AEC8276
Bucculatrix ulmella|INV05617|Portugal|BOLD:AEC8276
Bucculatrix ulmella|INV07584|Portugal|BOLD:AAU1882
Bucculatrix frangutella|INV03777|Portugal|BOLD:AAI8491
Miltochrista miniata|INV01327|Portugal|BOLD:AAC4781
Miltochrista miniata|INV05460|Portugal|BOLD:AAC4781
Miltochrista miniata|INV03303|Portugal|BOLD:AAC4781
Drymonia velitaris|INV00629|Portugal|BOLD:AAL1776
Drymonia querna|INV02438|Portugal|BOLD:ADT7493
Drymonia querna|INV00642|Portugal|BOLD:ADT7493
Drymonia querna|INV06527|Portugal|BOLD:ADT7493
Drymonia dodonaea|INV02401|Portugal|BOLD:AAD7692
Drymonia ruficornis|INV06648|Portugal|BOLD:AAD0066
Peridea anceps|INV04453|Portugal|BOLD:AAD9960
Peridea anceps|INV02478|Portugal|BOLD:AAD9960
Peridea anceps|INV04430|Portugal|BOLD:AAD9960
Peridea anceps|INV02482|Portugal|BOLD:AAD9960
Notodonta dromedarius|INV01119|Portugal|BOLD:AAC1145
Notodonta ziczac|INV04482|Portugal|BOLD:ABY9866
Notodonta ziczac|INV06533|Portugal|BOLD:ABY9866
Notodonta tritophus|INV03015|Portugal|BOLD:AAE2131
Clostera pigra|INV03212|Portugal|BOLD:AAD5412
Clostera pigra|INV06683|Portugal|BOLD:AAD5412
Mesophleps corsicella|INV07068|Portugal|BOLD:AAU3613
Gladiovalva badidorsella|INV02699|Portugal|BOLD:ADL9737
Gladiovalva badidorsella|INV00955|Portugal|BOLD:ADL9737
Gladiovalva badidorsella|INV07623|Portugal|BOLD:ADL9737
Agrotera nemoralis|INV03970|Portugal|BOLD:AAE4415
Agrotera nemoralis|INV05422|Portugal|BOLD:AAE4415
Agrotera nemoralis|INV03123|Portugal|BOLD:AAE4415
Sophronia humerella|INV00971|Portugal|BOLD:AAF4746
Sophronia humerella|INV00856|Portugal|BOLD:ADS5734
Sophronia humerella|INV03124|Portugal|BOLD:ADS5734
Pseudosophronia cosmella|INV08646|Portugal|BOLD:ADR7943
Mecyna auralis|INV02796|Portugal|BOLD:ACE9381
Mecyna asinalis|INV02853|Portugal|BOLD:AAK7059
Mecyna asinalis|INV07583|Portugal|BOLD:AAK7059
Mecyna asinalis|INV07624|Portugal|BOLD:AAK7059
Arnia nervosalis|INV01981|Portugal|BOLD:AEC9095
Arnia nervosalis|INV07480|Portugal|BOLD:AEC9095
Arnia nervosalis|INV07791|Portugal|BOLD:AEC9095
Stomopteryx basalis|INV02689|Portugal|BOLD:ACW1398
Stomopteryx remissella|INV00860|Portugal|BOLD:ADT9398
Stomopteryx remissella|INV06462|Portugal|BOLD:ADT9398
Stomopteryx remissella|INV08865|Portugal|BOLD:ADT9398
Mesophleps trinotella|INV03113|Portugal|BOLD:AAV7800
Aproaerema anthyllidella|INV01386|Portugal|BOLD:AAD2266
Aproaerema anthyllidella|INV00873|Portugal|BOLD:AAD2266
Aproaerema anthyllidella|INV02848|Portugal|BOLD:AAD2267
Aproaerema anthyllidella|INV00967|Portugal|BOLD:AAD2267
Stomopteryx lusitaniella|INV00784|Portugal|BOLD:ADR8328
Stomopteryx flavipalpella|INV00792|Portugal|BOLD:ADM8466
Stomopteryx flavipalpella|INV01202|Portugal|BOLD:ADM8466
Aproaerema buvati|INV00776|Portugal|BOLD:AEC7434
Aproaerema albipalpella|INV03836|Portugal|BOLD:AEC8234
Aproaerema larseniella|INV03175|Portugal|BOLD:AAK1468
Aproaerema larseniella|INV09838|Portugal|BOLD:AAK1468
Aproaerema larseniella|INV00600|Portugal|BOLD:AAK1468
Patania balteata|INV00915|Portugal|BOLD:AAB0533
Ecpyrrhorrhoe rubiginalis|INV02776|Portugal|BOLD:AAF6450
Ecpyrrhorrhoe rubiginalis|INV03311|Portugal|BOLD:AAF6450
Pyrausta aurata|INV02359|Portugal|BOLD:AAB6530
Pyrausta aurata|INV00927|Portugal|BOLD:AAB6530
Pyrausta purpuralis|INV00939|Portugal|BOLD:AAB6531
Pyrausta purpuralis|INV03694|Portugal|BOLD:AAB6531
Pyrausta sanguinalis|INV00822|Portugal|BOLD:AAI0560
Pyrausta sanguinalis|INV07019|Portugal|BOLD:AAI0560
Pyrausta sanguinalis|INV03355|Portugal|BOLD:AAI0560
Uresiphita gilvata|INV01946|Portugal|BOLD:ACF5204
Uresiphita gilvata|INV03155|Portugal|BOLD:ACF5204
Uresiphita gilvata|INV00900|Portugal|BOLD:ACF5204
Loxostege sticticalis|INV03339|Portugal|BOLD:AAB4167
Loxostege sticticalis|INV01942|Portugal|BOLD:AAB4167

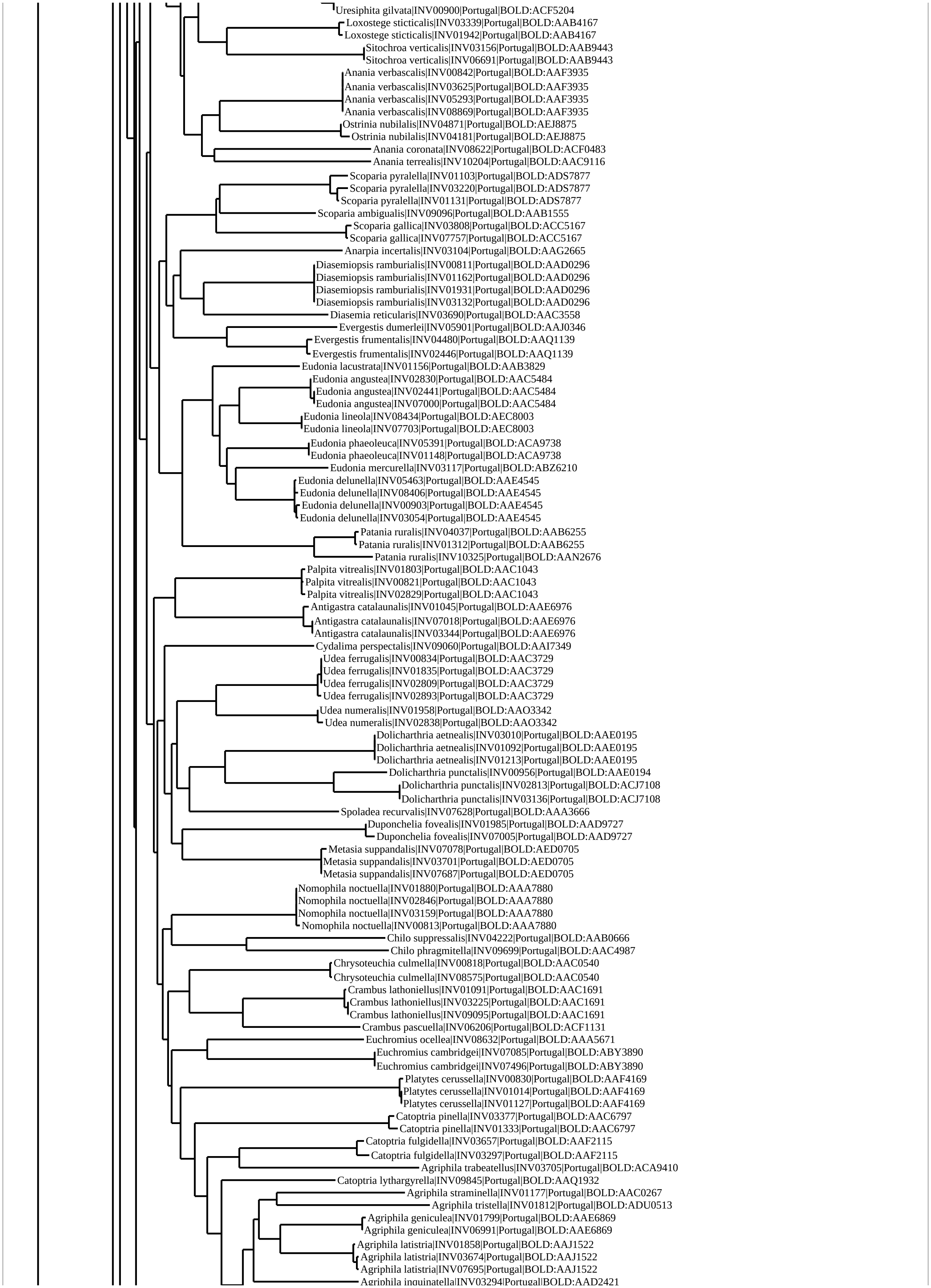
Uresiphita gilvata|INV00900|Portugal|BOLD:ACF5204
Loxostege sticticalis|INV03339|Portugal|BOLD:AAB4167
Loxostege sticticalis|INV01942|Portugal|BOLD:AAB4167
Sitochroa verticalis|INV03156|Portugal|BOLD:AAB9443
Sitochroa verticalis|INV06691|Portugal|BOLD:AAB9443
Anania verbascalis|INV00842|Portugal|BOLD:AAF3935
Anania verbascalis|INV03625|Portugal|BOLD:AAF3935
Anania verbascalis|INV05293|Portugal|BOLD:AAF3935
Anania verbascalis|INV08869|Portugal|BOLD:AAF3935
Ostrinia nubilalis|INV04871|Portugal|BOLD:AEJ8875
Ostrinia nubilalis|INV04181|Portugal|BOLD:AEJ8875
Anania coronata|INV08622|Portugal|BOLD:ACF0483
Anania terrealis|INV10204|Portugal|BOLD:AAC9116
Scoparia pyralella|INV01103|Portugal|BOLD:ADS7877
Scoparia pyralella|INV03220|Portugal|BOLD:ADS7877
Scoparia pyralella|INV01131|Portugal|BOLD:ADS7877
Scoparia ambigualis|INV09096|Portugal|BOLD:AAB1555
Scoparia gallica|INV03808|Portugal|BOLD:ACC5167
Scoparia gallica|INV07757|Portugal|BOLD:ACC5167
Anarpia incertalis|INV03104|Portugal|BOLD:AAG2665
Diasemiopsis ramburialis|INV00811|Portugal|BOLD:AAD0296
Diasemiopsis ramburialis|INV01162|Portugal|BOLD:AAD0296
Diasemiopsis ramburialis|INV01931|Portugal|BOLD:AAD0296
Diasemiopsis ramburialis|INV03132|Portugal|BOLD:AAD0296
Diasemia reticularis|INV03690|Portugal|BOLD:AAC3558
Evergestis dumerlei|INV05901|Portugal|BOLD:AAJ0346
Evergestis frumentalis|INV04480|Portugal|BOLD:AAQ1139
Evergestis frumentalis|INV02446|Portugal|BOLD:AAQ1139
Eudonia lacustrata|INV01156|Portugal|BOLD:AAB3829
Eudonia angustea|INV02830|Portugal|BOLD:AAC5484
Eudonia angustea|INV02441|Portugal|BOLD:AAC5484
Eudonia angustea|INV07000|Portugal|BOLD:AAC5484
Eudonia lineola|INV08434|Portugal|BOLD:AEC8003
Eudonia lineola|INV07703|Portugal|BOLD:AEC8003
Eudonia phaeoleuca|INV05391|Portugal|BOLD:ACA9738
Eudonia phaeoleuca|INV01148|Portugal|BOLD:ACA9738
Eudonia mercurella|INV03117|Portugal|BOLD:ABZ6210
Eudonia delunella|INV05463|Portugal|BOLD:AAE4545
Eudonia delunella|INV08406|Portugal|BOLD:AAE4545
Eudonia delunella|INV00903|Portugal|BOLD:AAE4545
Eudonia delunella|INV03054|Portugal|BOLD:AAE4545
Patania ruralis|INV04037|Portugal|BOLD:AAB6255
Patania ruralis|INV01312|Portugal|BOLD:AAB6255
Patania ruralis|INV10325|Portugal|BOLD:AAN2676
Palpita vitrealis|INV01803|Portugal|BOLD:AAC1043
Palpita vitrealis|INV00821|Portugal|BOLD:AAC1043
Palpita vitrealis|INV02829|Portugal|BOLD:AAC1043
Antigastra catalaunalis|INV01045|Portugal|BOLD:AAE6976
Antigastra catalaunalis|INV07018|Portugal|BOLD:AAE6976
Antigastra catalaunalis|INV03344|Portugal|BOLD:AAE6976
Cydalima perspectalis|INV09060|Portugal|BOLD:AAI7349
Udea ferrugalis|INV00834|Portugal|BOLD:AAC3729
Udea ferrugalis|INV01835|Portugal|BOLD:AAC3729
Udea ferrugalis|INV02809|Portugal|BOLD:AAC3729
Udea ferrugalis|INV02893|Portugal|BOLD:AAC3729
Udea numeralis|INV01958|Portugal|BOLD:AAO3342
Udea numeralis|INV02838|Portugal|BOLD:AAO3342
Dolicharthria aetnealis|INV03010|Portugal|BOLD:AAE0195
Dolicharthria aetnealis|INV01092|Portugal|BOLD:AAE0195
Dolicharthria aetnealis|INV01213|Portugal|BOLD:AAE0195
Dolicharthria punctalis|INV00956|Portugal|BOLD:AAE0194
Dolicharthria punctalis|INV02813|Portugal|BOLD:ACJ7108
Dolicharthria punctalis|INV03136|Portugal|BOLD:ACJ7108
Spoladea recurvalis|INV07628|Portugal|BOLD:AAA3666
Duponchelia fovealis|INV01985|Portugal|BOLD:AAD9727
Duponchelia fovealis|INV07005|Portugal|BOLD:AAD9727
Metasia suppandalis|INV07078|Portugal|BOLD:AED0705
Metasia suppandalis|INV03701|Portugal|BOLD:AED0705
Metasia suppandalis|INV07687|Portugal|BOLD:AED0705
Nomophila noctuella|INV01880|Portugal|BOLD:AAA7880
Nomophila noctuella|INV02846|Portugal|BOLD:AAA7880
Nomophila noctuella|INV03159|Portugal|BOLD:AAA7880
Nomophila noctuella|INV00813|Portugal|BOLD:AAA7880
Chilo suppressalis|INV04222|Portugal|BOLD:AAB0666
Chilo phragmitella|INV09699|Portugal|BOLD:AAC4987
Chrysoteuchia culmella|INV00818|Portugal|BOLD:AAC0540
Chrysoteuchia culmella|INV08575|Portugal|BOLD:AAC0540
Crambus lathoniellus|INV01091|Portugal|BOLD:AAC1691
Crambus lathoniellus|INV03225|Portugal|BOLD:AAC1691
Crambus lathoniellus|INV09095|Portugal|BOLD:AAC1691
Crambus pascuella|INV06206|Portugal|BOLD:ACF1131
Euchromius ocellea|INV08632|Portugal|BOLD:AAA5671
Euchromius cambridgei|INV07085|Portugal|BOLD:ABY3890
Euchromius cambridgei|INV07496|Portugal|BOLD:ABY3890
Platytes cerussella|INV00830|Portugal|BOLD:AAF4169
Platytes cerussella|INV01014|Portugal|BOLD:AAF4169
Platytes cerussella|INV01127|Portugal|BOLD:AAF4169
Catoptria pinella|INV03377|Portugal|BOLD:AAC6797
Catoptria pinella|INV01333|Portugal|BOLD:AAC6797
Catoptria fulgidella|INV03657|Portugal|BOLD:AAF2115
Catoptria fulgidella|INV03297|Portugal|BOLD:AAF2115
Agriphila trabeatellus|INV03705|Portugal|BOLD:ACA9410
Catoptria lythargyrella|INV09845|Portugal|BOLD:AAQ1932
Agriphila straminella|INV01177|Portugal|BOLD:AAC0267
Agriphila tristella|INV01812|Portugal|BOLD:ADU0513
Agriphila geniculea|INV01799|Portugal|BOLD:AAE6869
Agriphila geniculea|INV06991|Portugal|BOLD:AAE6869
Agriphila latistria|INV01858|Portugal|BOLD:AAJ1522
Agriphila latistria|INV03674|Portugal|BOLD:AAJ1522
Agriphila latistria|INV07695|Portugal|BOLD:AAJ1522
Agriphila inquinatella|INV03294|Portugal|BOLD:AAD2421

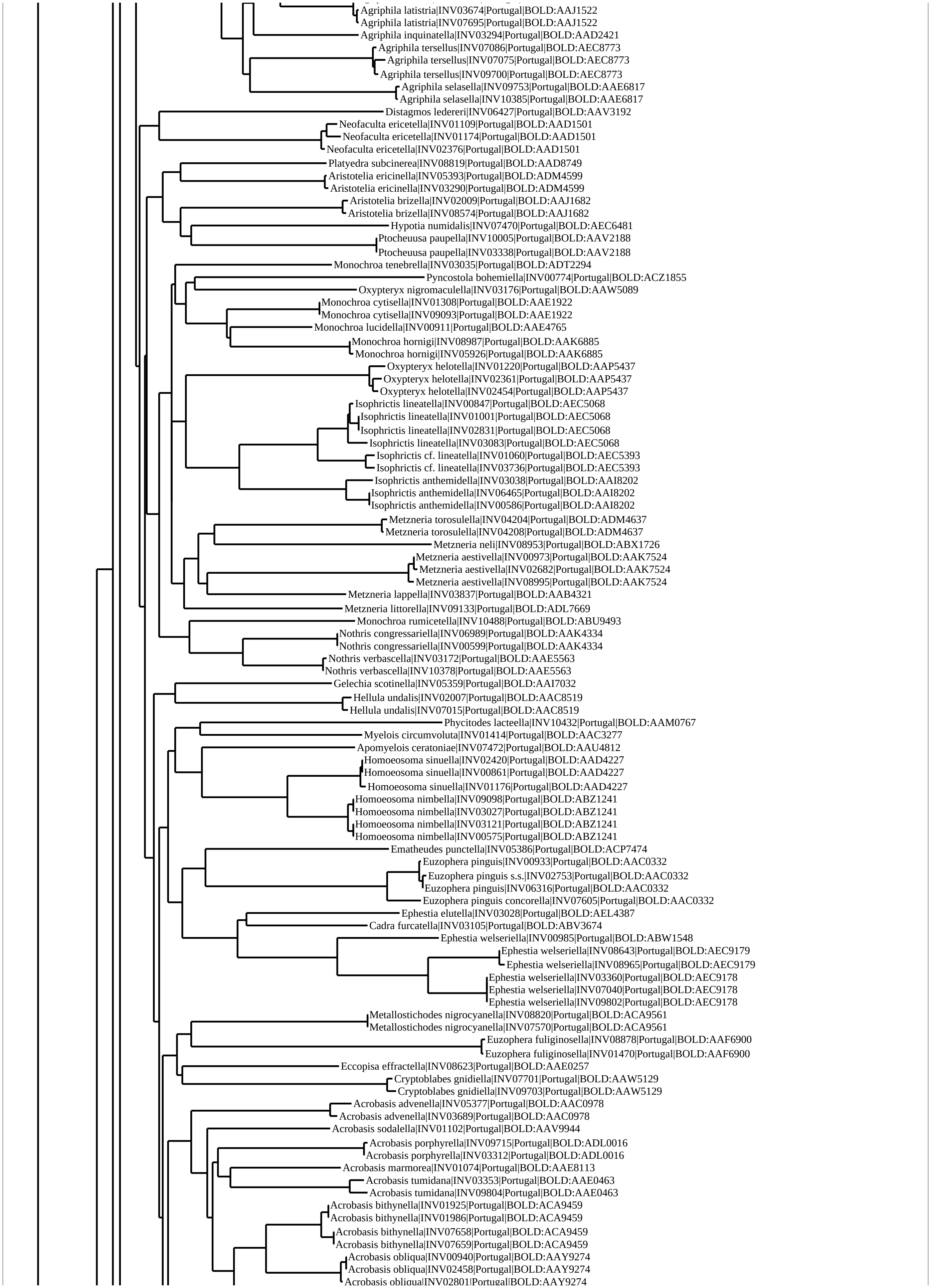
Agriphila latistria|INV03674|Portugal|BOLD:AAJ1522
Agriphila latistria|INV07695|Portugal|BOLD:AAJ1522
Agriphila inquinatella|INV03294|Portugal|BOLD:AAD2421
Agriphila tersellus|INV07086|Portugal|BOLD:AEC8773
Agriphila tersellus|INV07075|Portugal|BOLD:AEC8773
Agriphila tersellus|INV09700|Portugal|BOLD:AEC8773
Agriphila selasella|INV09753|Portugal|BOLD:AAE6817
Agriphila selasella|INV10385|Portugal|BOLD:AAE6817
Distagmos ledereri|INV06427|Portugal|BOLD:AAV3192
Neofaculta ericetella|INV01109|Portugal|BOLD:AAD1501
Neofaculta ericetella|INV01174|Portugal|BOLD:AAD1501
Neofaculta ericetella|INV02376|Portugal|BOLD:AAD1501
Platyedra subcinerea|INV08819|Portugal|BOLD:AAD8749
Aristotelia ericinella|INV05393|Portugal|BOLD:ADM4599
Aristotelia ericinella|INV03290|Portugal|BOLD:ADM4599
Aristotelia brizella|INV02009|Portugal|BOLD:AAJ1682
Aristotelia brizella|INV08574|Portugal|BOLD:AAJ1682
Hypotia numidalis|INV07470|Portugal|BOLD:AEC6481
Ptocheuusa paupella|INV10005|Portugal|BOLD:AAV2188
Ptocheuusa paupella|INV03338|Portugal|BOLD:AAV2188
Monochroa tenebrella|INV03035|Portugal|BOLD:ADT2294
Pyncostola bohemiella|INV00774|Portugal|BOLD:ACZ1855
Oxypteryx nigromaculella|INV03176|Portugal|BOLD:AAW5089
Monochroa cytisella|INV01308|Portugal|BOLD:AAE1922
Monochroa cytisella|INV09093|Portugal|BOLD:AAE1922
Monochroa lucidella|INV00911|Portugal|BOLD:AAE4765
Monochroa hornigi|INV08987|Portugal|BOLD:AAK6885
Monochroa hornigi|INV05926|Portugal|BOLD:AAK6885
Oxypteryx helotella|INV01220|Portugal|BOLD:AAP5437
Oxypteryx helotella|INV02361|Portugal|BOLD:AAP5437
Oxypteryx helotella|INV02454|Portugal|BOLD:AAP5437
Isophrictis lineatella|INV00847|Portugal|BOLD:AEC5068
Isophrictis lineatella|INV01001|Portugal|BOLD:AEC5068
Isophrictis lineatella|INV02831|Portugal|BOLD:AEC5068
Isophrictis lineatella|INV03083|Portugal|BOLD:AEC5068
Isophrictis cf. lineatella|INV01060|Portugal|BOLD:AEC5393
Isophrictis cf. lineatella|INV03736|Portugal|BOLD:AEC5393
Isophrictis anthemidella|INV03038|Portugal|BOLD:AAI8202
Isophrictis anthemidella|INV06465|Portugal|BOLD:AAI8202
Isophrictis anthemidella|INV00586|Portugal|BOLD:AAI8202
Metzneria torosulella|INV04204|Portugal|BOLD:ADM4637
Metzneria torosulella|INV04208|Portugal|BOLD:ADM4637
Metzneria neli|INV08953|Portugal|BOLD:ABX1726
Metzneria aestivella|INV00973|Portugal|BOLD:AAK7524
Metzneria aestivella|INV02682|Portugal|BOLD:AAK7524
Metzneria aestivella|INV08995|Portugal|BOLD:AAK7524
Metzneria lappella|INV03837|Portugal|BOLD:AAB4321
Metzneria littorella|INV09133|Portugal|BOLD:ADL7669
Monochroa rumicetella|INV10488|Portugal|BOLD:ABU9493
Nothris congressariella|INV06989|Portugal|BOLD:AAK4334
Nothris congressariella|INV00599|Portugal|BOLD:AAK4334
Nothris verbascella|INV03172|Portugal|BOLD:AAE5563
Nothris verbascella|INV10378|Portugal|BOLD:AAE5563
Gelechia scotinella|INV05359|Portugal|BOLD:AAI7032
Hellula undalis|INV02007|Portugal|BOLD:AAC8519
Hellula undalis|INV07015|Portugal|BOLD:AAC8519
Phycitodes lacteella|INV10432|Portugal|BOLD:AAM0767
Myelois circumvoluta|INV01414|Portugal|BOLD:AAC3277
Apomyelois ceratoniae|INV07472|Portugal|BOLD:AAU4812
Homoeosoma sinuella|INV02420|Portugal|BOLD:AAD4227
Homoeosoma sinuella|INV00861|Portugal|BOLD:AAD4227
Homoeosoma sinuella|INV01176|Portugal|BOLD:AAD4227
Homoeosoma nimbella|INV09098|Portugal|BOLD:ABZ1241
Homoeosoma nimbella|INV03027|Portugal|BOLD:ABZ1241
Homoeosoma nimbella|INV03121|Portugal|BOLD:ABZ1241
Homoeosoma nimbella|INV00575|Portugal|BOLD:ABZ1241
Ematheudes punctella|INV05386|Portugal|BOLD:ACP7474
Euzophera pinguis|INV00933|Portugal|BOLD:AAC0332
Euzophera pinguis s.s.|INV02753|Portugal|BOLD:AAC0332
Euzophera pinguis|INV06316|Portugal|BOLD:AAC0332
Euzophera pinguis concorella|INV07605|Portugal|BOLD:AAC0332
Ephestia elutella|INV03028|Portugal|BOLD:AEL4387
Cadra furcatella|INV03105|Portugal|BOLD:ABV3674
Ephestia welseriella|INV00985|Portugal|BOLD:ABW1548
Ephestia welseriella|INV08643|Portugal|BOLD:AEC9179
Ephestia welseriella|INV08965|Portugal|BOLD:AEC9179
Ephestia welseriella|INV03360|Portugal|BOLD:AEC9178
Ephestia welseriella|INV07040|Portugal|BOLD:AEC9178
Ephestia welseriella|INV09802|Portugal|BOLD:AEC9178
Metallostichodes nigrocyanella|INV08820|Portugal|BOLD:ACA9561
Metallostichodes nigrocyanella|INV07570|Portugal|BOLD:ACA9561
Euzophera fuliginosella|INV08878|Portugal|BOLD:AAF6900
Euzophera fuliginosella|INV01470|Portugal|BOLD:AAF6900
Eccopisa effractella|INV08623|Portugal|BOLD:AAE0257
Cryptoblabes gnidiella|INV07701|Portugal|BOLD:AAW5129
Cryptoblabes gnidiella|INV09703|Portugal|BOLD:AAW5129
Acrobasis advenella|INV05377|Portugal|BOLD:AAC0978
Acrobasis advenella|INV03689|Portugal|BOLD:AAC0978
Acrobasis sodalella|INV01102|Portugal|BOLD:AAV9944
Acrobasis porphyrella|INV09715|Portugal|BOLD:ADL0016
Acrobasis porphyrella|INV03312|Portugal|BOLD:ADL0016
Acrobasis marmorea|INV01074|Portugal|BOLD:AAE8113
Acrobasis tumidana|INV03353|Portugal|BOLD:AAE0463
Acrobasis tumidana|INV09804|Portugal|BOLD:AAE0463
Acrobasis bithynella|INV01925|Portugal|BOLD:ACA9459
Acrobasis bithynella|INV01986|Portugal|BOLD:ACA9459
Acrobasis bithynella|INV07658|Portugal|BOLD:ACA9459
Acrobasis bithynella|INV07659|Portugal|BOLD:ACA9459
Acrobasis obliqua|INV00940|Portugal|BOLD:AAY9274
Acrobasis obliqua|INV02458|Portugal|BOLD:AAY9274
Acrobasis obliqua|INV02801|Portugal|BOLD:AAY9274

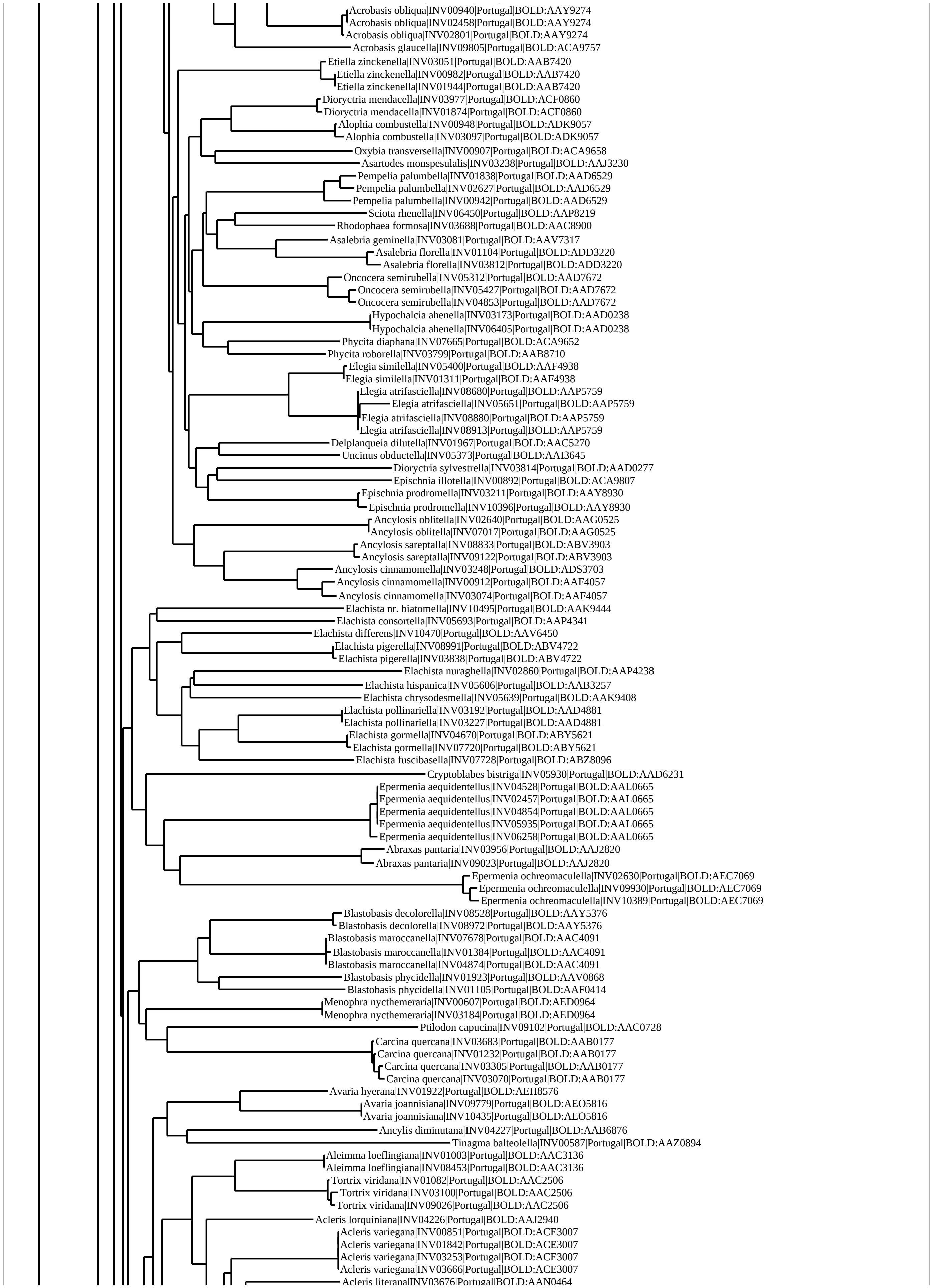
Acrobasis obliqua|INV00940|Portugal|BOLD:AAY9274
Acrobasis obliqua|INV02458|Portugal|BOLD:AAY9274
Acrobasis obliqua|INV02801|Portugal|BOLD:AAY9274
Acrobasis glaucella|INV09805|Portugal|BOLD:ACA9757
Etiella zinckenella|INV03051|Portugal|BOLD:AAB7420
Etiella zinckenella|INV00982|Portugal|BOLD:AAB7420
Etiella zinckenella|INV01944|Portugal|BOLD:AAB7420
Dioryctria mendacella|INV03977|Portugal|BOLD:ACF0860
Dioryctria mendacella|INV01874|Portugal|BOLD:ACF0860
Alophia combustella|INV00948|Portugal|BOLD:ADK9057
Alophia combustella|INV03097|Portugal|BOLD:ADK9057
Oxybia transversella|INV00907|Portugal|BOLD:ACA9658
Asartodes monspesulalis|INV03238|Portugal|BOLD:AAJ3230
Pempelia palumbella|INV01838|Portugal|BOLD:AAD6529
Pempelia palumbella|INV02627|Portugal|BOLD:AAD6529
Pempelia palumbella|INV00942|Portugal|BOLD:AAD6529
Sciota rhenella|INV06450|Portugal|BOLD:AAP8219
Rhodophaea formosa|INV03688|Portugal|BOLD:AAC8900
Asalebria geminella|INV03081|Portugal|BOLD:AAV7317
Asalebria florella|INV01104|Portugal|BOLD:ADD3220
Asalebria florella|INV03812|Portugal|BOLD:ADD3220
Oncocera semirubella|INV05312|Portugal|BOLD:AAD7672
Oncocera semirubella|INV05427|Portugal|BOLD:AAD7672
Oncocera semirubella|INV04853|Portugal|BOLD:AAD7672
Hypochalcia ahenella|INV03173|Portugal|BOLD:AAD0238
Hypochalcia ahenella|INV06405|Portugal|BOLD:AAD0238
Phycita diaphana|INV07665|Portugal|BOLD:ACA9652
Phycita roborella|INV03799|Portugal|BOLD:AAB8710
Elegia similella|INV05400|Portugal|BOLD:AAF4938
Elegia similella|INV01311|Portugal|BOLD:AAF4938
Elegia atrifasciella|INV08680|Portugal|BOLD:AAP5759
Elegia atrifasciella|INV05651|Portugal|BOLD:AAP5759
Elegia atrifasciella|INV08880|Portugal|BOLD:AAP5759
Elegia atrifasciella|INV08913|Portugal|BOLD:AAP5759
Delplanqueia dilutella|INV01967|Portugal|BOLD:AAC5270
Uncinus obductella|INV05373|Portugal|BOLD:AAI3645
Dioryctria sylvestrella|INV03814|Portugal|BOLD:AAD0277
Epischnia illotella|INV00892|Portugal|BOLD:ACA9807
Epischnia prodromella|INV03211|Portugal|BOLD:AAY8930
Epischnia prodromella|INV10396|Portugal|BOLD:AAY8930
Ancylosis oblitella|INV02640|Portugal|BOLD:AAG0525
Ancylosis oblitella|INV07017|Portugal|BOLD:AAG0525
Ancylosis sareptalla|INV08833|Portugal|BOLD:ABV3903
Ancylosis sareptalla|INV09122|Portugal|BOLD:ABV3903
Ancylosis cinnamomella|INV03248|Portugal|BOLD:ADS3703
Ancylosis cinnamomella|INV00912|Portugal|BOLD:AAF4057
Ancylosis cinnamomella|INV03074|Portugal|BOLD:AAF4057
Elachista nr. biatomella|INV10495|Portugal|BOLD:AAK9444
Elachista consortella|INV05693|Portugal|BOLD:AAP4341
Elachista differens|INV10470|Portugal|BOLD:AAV6450
Elachista pigerella|INV08991|Portugal|BOLD:ABV4722
Elachista pigerella|INV03838|Portugal|BOLD:ABV4722
Elachista nuraghella|INV02860|Portugal|BOLD:AAP4238
Elachista hispanica|INV05606|Portugal|BOLD:AAB3257
Elachista chrysodesmella|INV05639|Portugal|BOLD:AAK9408
Elachista pollinariella|INV03192|Portugal|BOLD:AAD4881
Elachista pollinariella|INV03227|Portugal|BOLD:AAD4881
Elachista gormella|INV04670|Portugal|BOLD:ABY5621
Elachista gormella|INV07720|Portugal|BOLD:ABY5621
Elachista fuscibasella|INV07728|Portugal|BOLD:ABZ8096
Cryptoblabes bistriga|INV05930|Portugal|BOLD:AAD6231
Epermenia aequidentellus|INV04528|Portugal|BOLD:AAL0665
Epermenia aequidentellus|INV02457|Portugal|BOLD:AAL0665
Epermenia aequidentellus|INV04854|Portugal|BOLD:AAL0665
Epermenia aequidentellus|INV05935|Portugal|BOLD:AAL0665
Epermenia aequidentellus|INV06258|Portugal|BOLD:AAL0665
Abraxas pantaria|INV03956|Portugal|BOLD:AAJ2820
Abraxas pantaria|INV09023|Portugal|BOLD:AAJ2820
Epermenia ochreomaculella|INV02630|Portugal|BOLD:AEC7069
Epermenia ochreomaculella|INV09930|Portugal|BOLD:AEC7069
Epermenia ochreomaculella|INV10389|Portugal|BOLD:AEC7069
Blastobasis decolorella|INV08528|Portugal|BOLD:AAY5376
Blastobasis decolorella|INV08972|Portugal|BOLD:AAY5376
Blastobasis maroccanella|INV07678|Portugal|BOLD:AAC4091
Blastobasis maroccanella|INV01384|Portugal|BOLD:AAC4091
Blastobasis maroccanella|INV04874|Portugal|BOLD:AAC4091
Blastobasis phycidella|INV01923|Portugal|BOLD:AAV0868
Blastobasis phycidella|INV01105|Portugal|BOLD:AAF0414
Menophra nycthemeraria|INV00607|Portugal|BOLD:AED0964
Menophra nycthemeraria|INV03184|Portugal|BOLD:AED0964
Ptilodon capucina|INV09102|Portugal|BOLD:AAC0728
Carcina quercana|INV03683|Portugal|BOLD:AAB0177
Carcina quercana|INV01232|Portugal|BOLD:AAB0177
Carcina quercana|INV03305|Portugal|BOLD:AAB0177
Carcina quercana|INV03070|Portugal|BOLD:AAB0177
Avaria hyerana|INV01922|Portugal|BOLD:AEH8576
Avaria joannisiana|INV09779|Portugal|BOLD:AEO5816
Avaria joannisiana|INV10435|Portugal|BOLD:AEO5816
Ancylis diminutana|INV04227|Portugal|BOLD:AAB6876
Tinagma balteolella|INV00587|Portugal|BOLD:AAZ0894
Aleimma loeflingiana|INV01003|Portugal|BOLD:AAC3136
Aleimma loeflingiana|INV08453|Portugal|BOLD:AAC3136
Tortrix viridana|INV01082|Portugal|BOLD:AAC2506
Tortrix viridana|INV03100|Portugal|BOLD:AAC2506
Tortrix viridana|INV09026|Portugal|BOLD:AAC2506
Acleris lorquiniana|INV04226|Portugal|BOLD:AAJ2940
Acleris variegana|INV00851|Portugal|BOLD:ACE3007
Acleris variegana|INV01842|Portugal|BOLD:ACE3007
Acleris variegana|INV03253|Portugal|BOLD:ACE3007
Acleris variegana|INV03666|Portugal|BOLD:ACE3007
Acleris literana|INV03676|Portugal|BOLD:AAN0464

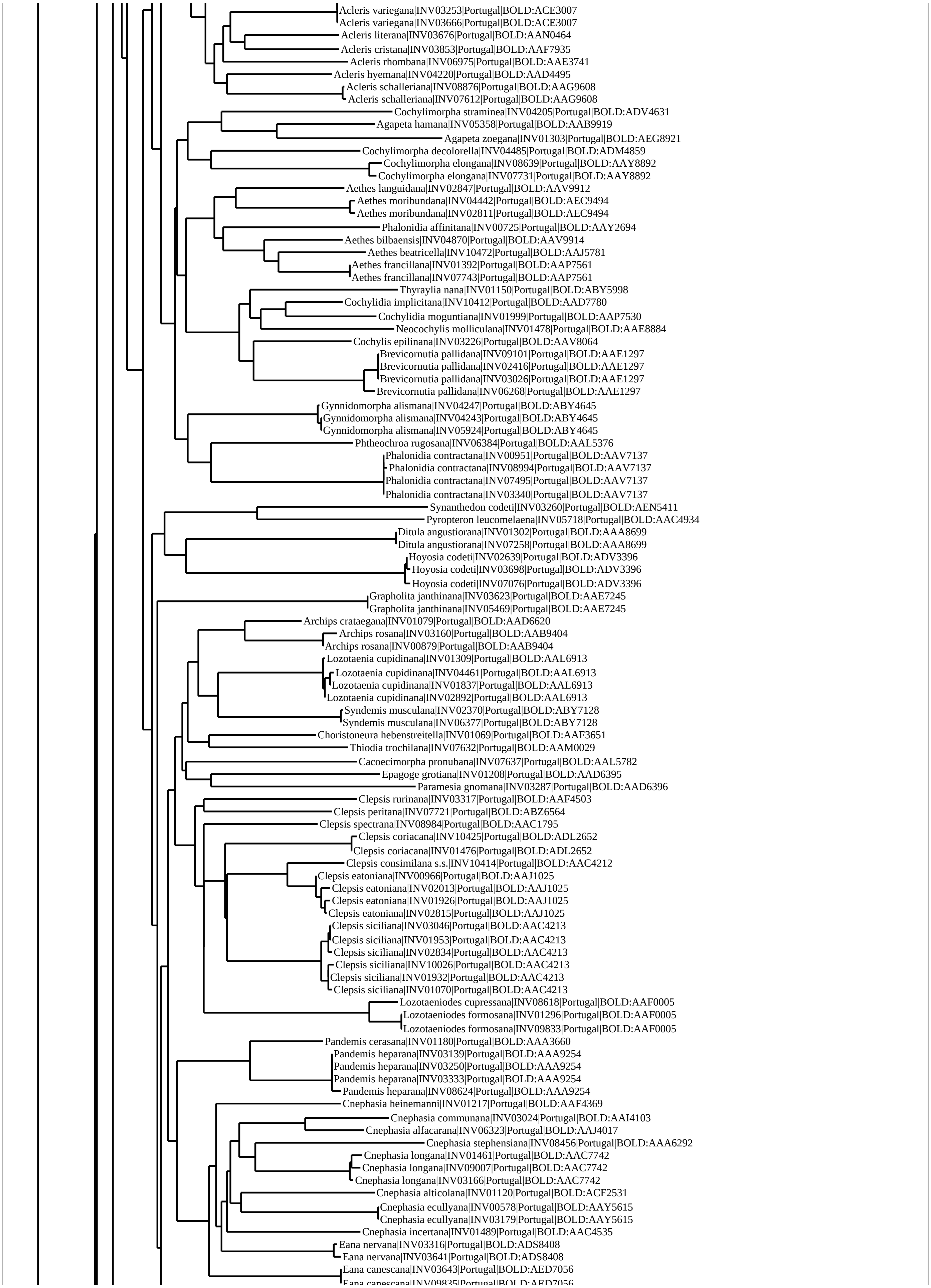
Acleris variegana|INV03253|Portugal|BOLD:ACE3007
Acleris variegana|INV03666|Portugal|BOLD:ACE3007
Acleris literana|INV03676|Portugal|BOLD:AAN0464
Acleris cristana|INV03853|Portugal|BOLD:AAF7935
Acleris rhombana|INV06975|Portugal|BOLD:AAE3741
Acleris hyemana|INV04220|Portugal|BOLD:AAD4495
Acleris schalleriana|INV08876|Portugal|BOLD:AAG9608
Acleris schalleriana|INV07612|Portugal|BOLD:AAG9608
Cochylimorpha straminea|INV04205|Portugal|BOLD:ADV4631
Agapeta hamana|INV05358|Portugal|BOLD:AAB9919
Agapeta zoegana|INV01303|Portugal|BOLD:AEG8921
Cochylimorpha decolorella|INV04485|Portugal|BOLD:ADM4859
Cochylimorpha elongana|INV08639|Portugal|BOLD:AAY8892
Cochylimorpha elongana|INV07731|Portugal|BOLD:AAY8892
Aethes languidana|INV02847|Portugal|BOLD:AAV9912
Aethes moribundana|INV04442|Portugal|BOLD:AEC9494
Aethes moribundana|INV02811|Portugal|BOLD:AEC9494
Phalonidia affinitana|INV00725|Portugal|BOLD:AAY2694
Aethes bilbaensis|INV04870|Portugal|BOLD:AAV9914
Aethes beatricella|INV10472|Portugal|BOLD:AAJ5781
Aethes francillana|INV01392|Portugal|BOLD:AAP7561
Aethes francillana|INV07743|Portugal|BOLD:AAP7561
Thyraylia nana|INV01150|Portugal|BOLD:ABY5998
Cochylidia implicitana|INV10412|Portugal|BOLD:AAD7780
Cochylidia moguntiana|INV01999|Portugal|BOLD:AAP7530
Neocochylis molliculana|INV01478|Portugal|BOLD:AAE8884
Cochylis epilinana|INV03226|Portugal|BOLD:AAV8064
Brevicornutia pallidana|INV09101|Portugal|BOLD:AAE1297
Brevicornutia pallidana|INV02416|Portugal|BOLD:AAE1297
Brevicornutia pallidana|INV03026|Portugal|BOLD:AAE1297
Brevicornutia pallidana|INV06268|Portugal|BOLD:AAE1297
Gynnidomorpha alismana|INV04247|Portugal|BOLD:ABY4645
Gynnidomorpha alismana|INV04243|Portugal|BOLD:ABY4645
Gynnidomorpha alismana|INV05924|Portugal|BOLD:ABY4645
Phtheochroa rugosana|INV06384|Portugal|BOLD:AAL5376
Phalonidia contractana|INV00951|Portugal|BOLD:AAV7137
Phalonidia contractana|INV08994|Portugal|BOLD:AAV7137
Phalonidia contractana|INV07495|Portugal|BOLD:AAV7137
Phalonidia contractana|INV03340|Portugal|BOLD:AAV7137
Synanthedon codeti|INV03260|Portugal|BOLD:AEN5411
Pyropteron leucomelaena|INV05718|Portugal|BOLD:AAC4934
Ditula angustiorana|INV01302|Portugal|BOLD:AAA8699
Ditula angustiorana|INV07258|Portugal|BOLD:AAA8699
Hoyosia codeti|INV02639|Portugal|BOLD:ADV3396
Hoyosia codeti|INV03698|Portugal|BOLD:ADV3396
Hoyosia codeti|INV07076|Portugal|BOLD:ADV3396
Grapholita janthinana|INV03623|Portugal|BOLD:AAE7245
Grapholita janthinana|INV05469|Portugal|BOLD:AAE7245
Archips crataegana|INV01079|Portugal|BOLD:AAD6620
Archips rosana|INV03160|Portugal|BOLD:AAB9404
Archips rosana|INV00879|Portugal|BOLD:AAB9404
Lozotaenia cupidinana|INV01309|Portugal|BOLD:AAL6913
Lozotaenia cupidinana|INV04461|Portugal|BOLD:AAL6913
Lozotaenia cupidinana|INV01837|Portugal|BOLD:AAL6913
Lozotaenia cupidinana|INV02892|Portugal|BOLD:AAL6913
Syndemis musculana|INV02370|Portugal|BOLD:ABY7128
Syndemis musculana|INV06377|Portugal|BOLD:ABY7128
Choristoneura hebenstreitella|INV01069|Portugal|BOLD:AAF3651
Thiodia trochilana|INV07632|Portugal|BOLD:AAM0029
Cacoecimorpha pronubana|INV07637|Portugal|BOLD:AAL5782
Epagoge grotiana|INV01208|Portugal|BOLD:AAD6395
Paramesia gnomana|INV03287|Portugal|BOLD:AAD6396
Clepsis rurinana|INV03317|Portugal|BOLD:AAF4503
Clepsis peritana|INV07721|Portugal|BOLD:ABZ6564
Clepsis spectrana|INV08984|Portugal|BOLD:AAC1795
Clepsis coriacana|INV10425|Portugal|BOLD:ADL2652
Clepsis coriacana|INV01476|Portugal|BOLD:ADL2652
Clepsis consimilana s.s.|INV10414|Portugal|BOLD:AAC4212
Clepsis eatoniana|INV00966|Portugal|BOLD:AAJ1025
Clepsis eatoniana|INV02013|Portugal|BOLD:AAJ1025
Clepsis eatoniana|INV01926|Portugal|BOLD:AAJ1025
Clepsis eatoniana|INV02815|Portugal|BOLD:AAJ1025
Clepsis siciliana|INV03046|Portugal|BOLD:AAC4213
Clepsis siciliana|INV01953|Portugal|BOLD:AAC4213
Clepsis siciliana|INV02834|Portugal|BOLD:AAC4213
Clepsis siciliana|INV10026|Portugal|BOLD:AAC4213
Clepsis siciliana|INV01932|Portugal|BOLD:AAC4213
Clepsis siciliana|INV01070|Portugal|BOLD:AAC4213
Lozotaeniodes cupressana|INV08618|Portugal|BOLD:AAF0005
Lozotaeniodes formosana|INV01296|Portugal|BOLD:AAF0005
Lozotaeniodes formosana|INV09833|Portugal|BOLD:AAF0005
Pandemis cerasana|INV01180|Portugal|BOLD:AAA3660
Pandemis heparana|INV03139|Portugal|BOLD:AAA9254
Pandemis heparana|INV03250|Portugal|BOLD:AAA9254
Pandemis heparana|INV03333|Portugal|BOLD:AAA9254
Pandemis heparana|INV08624|Portugal|BOLD:AAA9254
Cnephasia heinemanni|INV01217|Portugal|BOLD:AAF4369
Cnephasia communana|INV03024|Portugal|BOLD:AAI4103
Cnephasia alfacarana|INV06323|Portugal|BOLD:AAJ4017
Cnephasia stephensiana|INV08456|Portugal|BOLD:AAA6292
Cnephasia longana|INV01461|Portugal|BOLD:AAC7742
Cnephasia longana|INV09007|Portugal|BOLD:AAC7742
Cnephasia longana|INV03166|Portugal|BOLD:AAC7742
Cnephasia alticolana|INV01120|Portugal|BOLD:ACF2531
Cnephasia ecullyana|INV00578|Portugal|BOLD:AAY5615
Cnephasia ecullyana|INV03179|Portugal|BOLD:AAY5615
Cnephasia incertana|INV01489|Portugal|BOLD:AAC4535
Eana nervana|INV03316|Portugal|BOLD:ADS8408
Eana nervana|INV03641|Portugal|BOLD:ADS8408
Eana canescana|INV03643|Portugal|BOLD:AED7056
Eana canescana|INV09835|Portugal|BOLD:AED7056

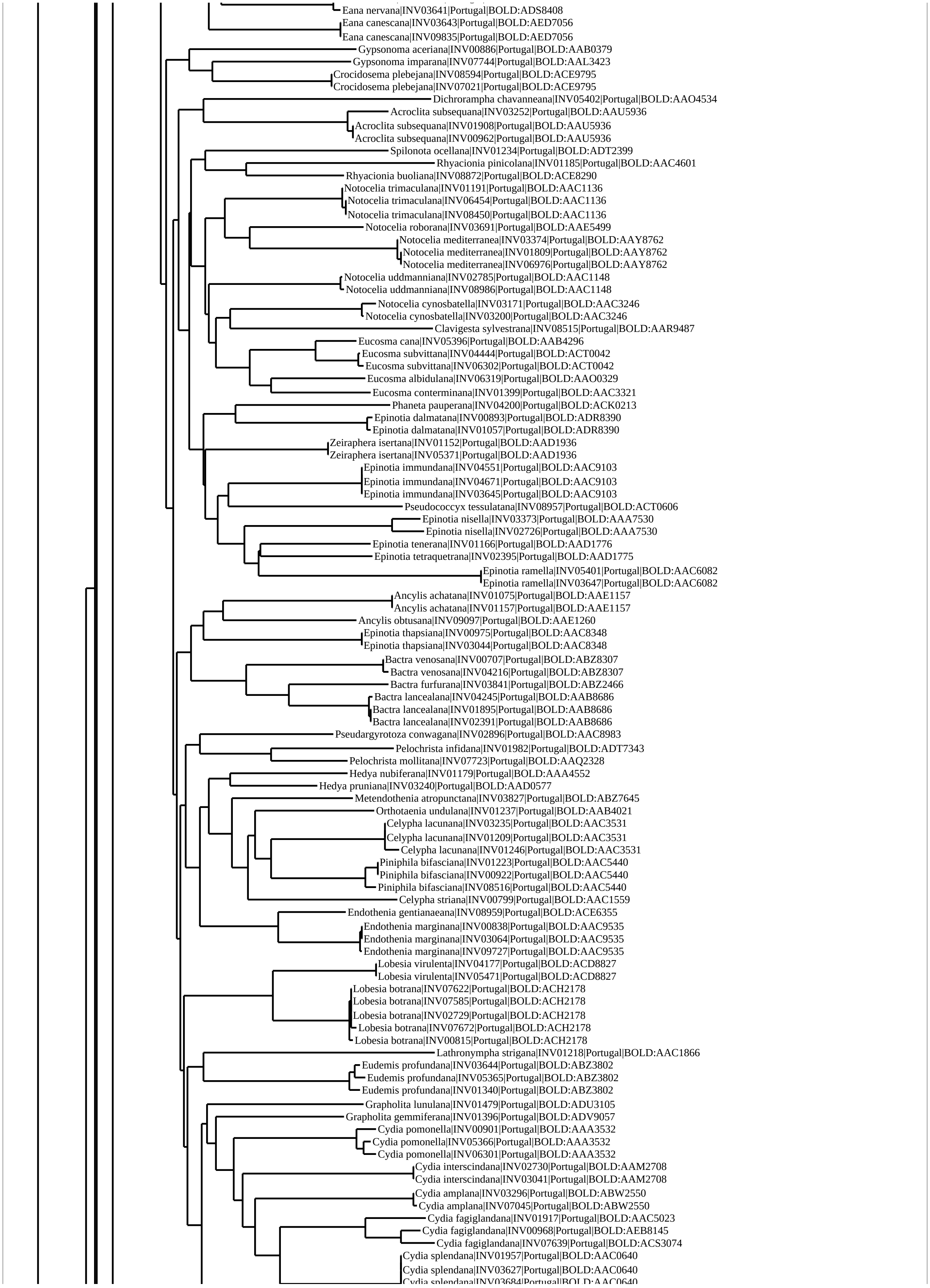
Eana nervana|INV03641|Portugal|BOLD:ADS8408
Eana canescana|INV03643|Portugal|BOLD:AED7056
Eana canescana|INV09835|Portugal|BOLD:AED7056
Gypsonoma aceriana|INV00886|Portugal|BOLD:AAB0379
Gypsonoma imparana|INV07744|Portugal|BOLD:AAL3423
Crocidosema plebejana|INV08594|Portugal|BOLD:ACE9795
Crocidosema plebejana|INV07021|Portugal|BOLD:ACE9795
Dichrorampha chavanneana|INV05402|Portugal|BOLD:AAO4534
Acroclita subsequana|INV03252|Portugal|BOLD:AAU5936
Acroclita subsequana|INV01908|Portugal|BOLD:AAU5936
Acroclita subsequana|INV00962|Portugal|BOLD:AAU5936
Spilonota ocellana|INV01234|Portugal|BOLD:ADT2399
Rhyacionia pinicolana|INV01185|Portugal|BOLD:AAC4601
Rhyacionia buoliana|INV08872|Portugal|BOLD:ACE8290
Notocelia trimaculana|INV01191|Portugal|BOLD:AAC1136
Notocelia trimaculana|INV06454|Portugal|BOLD:AAC1136
Notocelia trimaculana|INV08450|Portugal|BOLD:AAC1136
Notocelia roborana|INV03691|Portugal|BOLD:AAE5499
Notocelia mediterranea|INV03374|Portugal|BOLD:AAY8762
Notocelia mediterranea|INV01809|Portugal|BOLD:AAY8762
Notocelia mediterranea|INV06976|Portugal|BOLD:AAY8762
Notocelia uddmanniana|INV02785|Portugal|BOLD:AAC1148
Notocelia uddmanniana|INV08986|Portugal|BOLD:AAC1148
Notocelia cynosbatella|INV03171|Portugal|BOLD:AAC3246
Notocelia cynosbatella|INV03200|Portugal|BOLD:AAC3246
Clavigesta sylvestrana|INV08515|Portugal|BOLD:AAR9487
Eucosma cana|INV05396|Portugal|BOLD:AAB4296
Eucosma subvittana|INV04444|Portugal|BOLD:ACT0042
Eucosma subvittana|INV06302|Portugal|BOLD:ACT0042
Eucosma albidulana|INV06319|Portugal|BOLD:AAO0329
Eucosma conterminana|INV01399|Portugal|BOLD:AAC3321
Phaneta pauperana|INV04200|Portugal|BOLD:ACK0213
Epinotia dalmatana|INV00893|Portugal|BOLD:ADR8390
Epinotia dalmatana|INV01057|Portugal|BOLD:ADR8390
Zeiraphera isertana|INV01152|Portugal|BOLD:AAD1936
Zeiraphera isertana|INV05371|Portugal|BOLD:AAD1936
Epinotia immundana|INV04551|Portugal|BOLD:AAC9103
Epinotia immundana|INV04671|Portugal|BOLD:AAC9103
Epinotia immundana|INV03645|Portugal|BOLD:AAC9103
Pseudococcyx tessulatana|INV08957|Portugal|BOLD:ACT0606
Epinotia nisella|INV03373|Portugal|BOLD:AAA7530
Epinotia nisella|INV02726|Portugal|BOLD:AAA7530
Epinotia tenerana|INV01166|Portugal|BOLD:AAD1776
Epinotia tetraquetrana|INV02395|Portugal|BOLD:AAD1775
Epinotia ramella|INV05401|Portugal|BOLD:AAC6082
Epinotia ramella|INV03647|Portugal|BOLD:AAC6082
Ancylis achatana|INV01075|Portugal|BOLD:AAE1157
Ancylis achatana|INV01157|Portugal|BOLD:AAE1157
Ancylis obtusana|INV09097|Portugal|BOLD:AAE1260
Epinotia thapsiana|INV00975|Portugal|BOLD:AAC8348
Epinotia thapsiana|INV03044|Portugal|BOLD:AAC8348
Bactra venosana|INV00707|Portugal|BOLD:ABZ8307
Bactra venosana|INV04216|Portugal|BOLD:ABZ8307
Bactra furfurana|INV03841|Portugal|BOLD:ABZ2466
Bactra lancealana|INV04245|Portugal|BOLD:AAB8686
Bactra lancealana|INV01895|Portugal|BOLD:AAB8686
Bactra lancealana|INV02391|Portugal|BOLD:AAB8686
Pseudargyrotoza conwagana|INV02896|Portugal|BOLD:AAC8983
Pelochrista infidana|INV01982|Portugal|BOLD:ADT7343
Pelochrista mollitana|INV07723|Portugal|BOLD:AAQ2328
Hedya nubiferana|INV01179|Portugal|BOLD:AAA4552
Hedya pruniana|INV03240|Portugal|BOLD:AAD0577
Metendothenia atropunctana|INV03827|Portugal|BOLD:ABZ7645
Orthotaenia undulana|INV01237|Portugal|BOLD:AAB4021
Celypha lacunana|INV03235|Portugal|BOLD:AAC3531
Celypha lacunana|INV01209|Portugal|BOLD:AAC3531
Celypha lacunana|INV01246|Portugal|BOLD:AAC3531
Piniphila bifasciana|INV01223|Portugal|BOLD:AAC5440
Piniphila bifasciana|INV00922|Portugal|BOLD:AAC5440
Piniphila bifasciana|INV08516|Portugal|BOLD:AAC5440
Celypha striana|INV00799|Portugal|BOLD:AAC1559
Endothenia gentianaeana|INV08959|Portugal|BOLD:ACE6355
Endothenia marginana|INV00838|Portugal|BOLD:AAC9535
Endothenia marginana|INV03064|Portugal|BOLD:AAC9535
Endothenia marginana|INV09727|Portugal|BOLD:AAC9535
Lobesia virulenta|INV04177|Portugal|BOLD:ACD8827
Lobesia virulenta|INV05471|Portugal|BOLD:ACD8827
Lobesia botrana|INV07622|Portugal|BOLD:ACH2178
Lobesia botrana|INV07585|Portugal|BOLD:ACH2178
Lobesia botrana|INV02729|Portugal|BOLD:ACH2178
Lobesia botrana|INV07672|Portugal|BOLD:ACH2178
Lobesia botrana|INV00815|Portugal|BOLD:ACH2178
Lathronympha strigana|INV01218|Portugal|BOLD:AAC1866
Eudemis profundana|INV03644|Portugal|BOLD:ABZ3802
Eudemis profundana|INV05365|Portugal|BOLD:ABZ3802
Eudemis profundana|INV01340|Portugal|BOLD:ABZ3802
Grapholita lunulana|INV01479|Portugal|BOLD:ADU3105
Grapholita gemmiferana|INV01396|Portugal|BOLD:ADV9057
Cydia pomonella|INV00901|Portugal|BOLD:AAA3532
Cydia pomonella|INV05366|Portugal|BOLD:AAA3532
Cydia pomonella|INV06301|Portugal|BOLD:AAA3532
Cydia interscindana|INV02730|Portugal|BOLD:AAM2708
Cydia interscindana|INV03041|Portugal|BOLD:AAM2708
Cydia amplana|INV03296|Portugal|BOLD:ABW2550
Cydia amplana|INV07045|Portugal|BOLD:ABW2550
Cydia fagiglandana|INV01917|Portugal|BOLD:AAC5023
Cydia fagiglandana|INV00968|Portugal|BOLD:AEB8145
Cydia fagiglandana|INV07639|Portugal|BOLD:ACS3074
Cydia splendana|INV01957|Portugal|BOLD:AAC0640
Cydia splendana|INV03627|Portugal|BOLD:AAC0640
Cydia splendana|INV03684|Portugal|BOLD:AAC0640

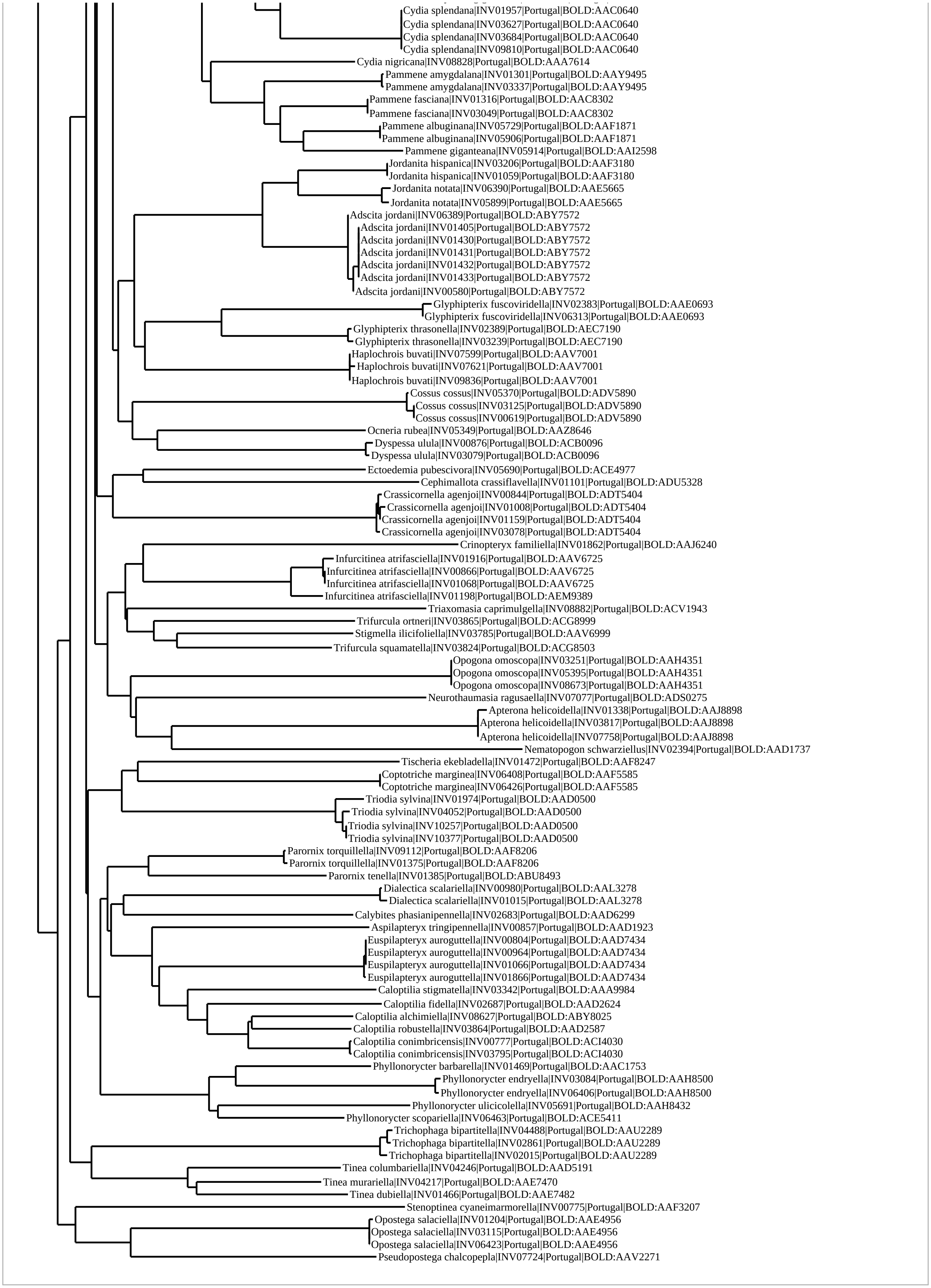
Cydia splendana|INV01957|Portugal|BOLD:AAC0640
Cydia splendana|INV03627|Portugal|BOLD:AAC0640
Cydia splendana|INV03684|Portugal|BOLD:AAC0640
Cydia splendana|INV09810|Portugal|BOLD:AAC0640
Cydia nigricana|INV08828|Portugal|BOLD:AAA7614
Pammene amygdalana|INV01301|Portugal|BOLD:AAY9495
Pammene amygdalana|INV03337|Portugal|BOLD:AAY9495
Pammene fasciana|INV01316|Portugal|BOLD:AAC8302
Pammene fasciana|INV03049|Portugal|BOLD:AAC8302
Pammene albuginana|INV05729|Portugal|BOLD:AAF1871
Pammene albuginana|INV05906|Portugal|BOLD:AAF1871
Pammene giganteana|INV05914|Portugal|BOLD:AAI2598
Jordanita hispanica|INV03206|Portugal|BOLD:AAF3180
Jordanita hispanica|INV01059|Portugal|BOLD:AAF3180
Jordanita notata|INV06390|Portugal|BOLD:AAE5665
Jordanita notata|INV05899|Portugal|BOLD:AAE5665
Adscita jordani|INV06389|Portugal|BOLD:ABY7572
Adscita jordani|INV01405|Portugal|BOLD:ABY7572
Adscita jordani|INV01430|Portugal|BOLD:ABY7572
Adscita jordani|INV01431|Portugal|BOLD:ABY7572
Adscita jordani|INV01432|Portugal|BOLD:ABY7572
Adscita jordani|INV01433|Portugal|BOLD:ABY7572
Adscita jordani|INV00580|Portugal|BOLD:ABY7572
Glyphipterix fuscoviridella|INV02383|Portugal|BOLD:AAE0693
Glyphipterix fuscoviridella|INV06313|Portugal|BOLD:AAE0693
Glyphipterix thrasonella|INV02389|Portugal|BOLD:AEC7190
Glyphipterix thrasonella|INV03239|Portugal|BOLD:AEC7190
Haplochrois buvati|INV07599|Portugal|BOLD:AAV7001
Haplochrois buvati|INV07621|Portugal|BOLD:AAV7001
Haplochrois buvati|INV09836|Portugal|BOLD:AAV7001
Cossus cossus|INV05370|Portugal|BOLD:ADV5890
Cossus cossus|INV03125|Portugal|BOLD:ADV5890
Cossus cossus|INV00619|Portugal|BOLD:ADV5890
Ocneria rubea|INV05349|Portugal|BOLD:AAZ8646
Dyspessa ulula|INV00876|Portugal|BOLD:ACB0096
Dyspessa ulula|INV03079|Portugal|BOLD:ACB0096
Ectoedemia pubescivora|INV05690|Portugal|BOLD:ACE4977
Cephimallota crassiflavella|INV01101|Portugal|BOLD:ADU5328
Crassicornella agenjoi|INV00844|Portugal|BOLD:ADT5404
Crassicornella agenjoi|INV01008|Portugal|BOLD:ADT5404
Crassicornella agenjoi|INV01159|Portugal|BOLD:ADT5404
Crassicornella agenjoi|INV03078|Portugal|BOLD:ADT5404
Crinopteryx familiella|INV01862|Portugal|BOLD:AAJ6240
Infurcitinea atrifasciella|INV01916|Portugal|BOLD:AAV6725
Infurcitinea atrifasciella|INV00866|Portugal|BOLD:AAV6725
Infurcitinea atrifasciella|INV01068|Portugal|BOLD:AAV6725
Infurcitinea atrifasciella|INV01198|Portugal|BOLD:AEM9389
Triaxomasia caprimulgella|INV08882|Portugal|BOLD:ACV1943
Trifurcula ortneri|INV03865|Portugal|BOLD:ACG8999
Stigmella ilicifoliella|INV03785|Portugal|BOLD:AAV6999
Trifurcula squamatella|INV03824|Portugal|BOLD:ACG8503
Opogona omoscopa|INV03251|Portugal|BOLD:AAH4351
Opogona omoscopa|INV05395|Portugal|BOLD:AAH4351
Opogona omoscopa|INV08673|Portugal|BOLD:AAH4351
Neurothaumasia ragusaella|INV07077|Portugal|BOLD:ADS0275
Apterona helicoidella|INV01338|Portugal|BOLD:AAJ8898
Apterona helicoidella|INV03817|Portugal|BOLD:AAJ8898
Apterona helicoidella|INV07758|Portugal|BOLD:AAJ8898
Nematopogon schwarziellus|INV02394|Portugal|BOLD:AAD1737
Tischeria ekebladella|INV01472|Portugal|BOLD:AAF8247
Coptotriche marginea|INV06408|Portugal|BOLD:AAF5585
Coptotriche marginea|INV06426|Portugal|BOLD:AAF5585
Triodia sylvina|INV01974|Portugal|BOLD:AAD0500
Triodia sylvina|INV04052|Portugal|BOLD:AAD0500
Triodia sylvina|INV10257|Portugal|BOLD:AAD0500
Triodia sylvina|INV10377|Portugal|BOLD:AAD0500
Parornix torquillella|INV09112|Portugal|BOLD:AAF8206
Parornix torquillella|INV01375|Portugal|BOLD:AAF8206
Parornix tenella|INV01385|Portugal|BOLD:ABU8493
Dialectica scalariella|INV00980|Portugal|BOLD:AAL3278
Dialectica scalariella|INV01015|Portugal|BOLD:AAL3278
Calybites phasianipennella|INV02683|Portugal|BOLD:AAD6299
Aspilapteryx tringipennella|INV00857|Portugal|BOLD:AAD1923
Euspilapteryx auroguttella|INV00804|Portugal|BOLD:AAD7434
Euspilapteryx auroguttella|INV00964|Portugal|BOLD:AAD7434
Euspilapteryx auroguttella|INV01066|Portugal|BOLD:AAD7434
Euspilapteryx auroguttella|INV01866|Portugal|BOLD:AAD7434
Caloptilia stigmatella|INV03342|Portugal|BOLD:AAA9984
Caloptilia fidella|INV02687|Portugal|BOLD:AAD2624
Caloptilia alchimiella|INV08627|Portugal|BOLD:ABY8025
Caloptilia robustella|INV03864|Portugal|BOLD:AAD2587
Caloptilia conimbricensis|INV00777|Portugal|BOLD:ACI4030
Caloptilia conimbricensis|INV03795|Portugal|BOLD:ACI4030
Phyllonorycter barbarella|INV01469|Portugal|BOLD:AAC1753
Phyllonorycter endryella|INV03084|Portugal|BOLD:AAH8500
Phyllonorycter endryella|INV06406|Portugal|BOLD:AAH8500
Phyllonorycter ulicicolella|INV05691|Portugal|BOLD:AAH8432
Phyllonorycter scopariella|INV06463|Portugal|BOLD:ACE5411
Trichophaga bipartitella|INV04488|Portugal|BOLD:AAU2289
Trichophaga bipartitella|INV02861|Portugal|BOLD:AAU2289
Trichophaga bipartitella|INV02015|Portugal|BOLD:AAU2289
Tinea columbariella|INV04246|Portugal|BOLD:AAD5191
Tinea murariella|INV04217|Portugal|BOLD:AAE7470
Tinea dubiella|INV01466|Portugal|BOLD:AAE7482
Stenoptinea cyaneimarmorella|INV00775|Portugal|BOLD:AAF3207
Opostega salaciella|INV01204|Portugal|BOLD:AAE4956
Opostega salaciella|INV03115|Portugal|BOLD:AAE4956
Opostega salaciella|INV06423|Portugal|BOLD:AAE4956
Pseudopostega chalcopepla|INV07724|Portugal|BOLD:AAV2271
